# Quantitative Compensation of Edge Effects for Particle Sizing in Stochastic Electrochemical Collision Experiments

**DOI:** 10.1021/acsmeasuresciau.6c00290

**Published:** 2026-09-11

**Authors:** Alfredo Eduardo Chávez-Ramos, Jake Saddler, Drake Katt, Mónica Galicia, César Alfonso Ortiz-Ledón

**Affiliations:** † Department of Chemistry and Biochemistry, 2344 Eastern Illinois University, Charleston, Illinois 61920, United States; ‡ Departamento de Ciencias Químico-Biológicas, Instituto de Ciencias Biomédicas, Universidad Autónoma de Ciudad Juárez, Ciudad Juárez, Chihuahua 32310, México

**Keywords:** Edge effect, stochastic electrochemistry, particle sizing, compensation, ultramicroelectrode

## Abstract

Stochastic electrochemistry has emerged as an important area of fundamental electrochemistry over the past decade. Understanding electrochemical processes on the nanoscale is critical for the development of sensors and single-molecule electrochemical methods. In addition, collisions of insulating particles with ultramicroelectrodes (UMEs) can be used to estimate the particle size by analyzing the current step generated when an insulating particle blocks a portion of the electrode surface. However, accurate particle-size determination is hindered by the edge effect, whereby the diffusional flux is greater at the edge of the UME than at its center, leading to inaccuracies in blocking collision data analysis. In this study, we present an analytical treatment of edge effects for the estimation of polymeric particle size using current-step measurements obtained from stochastic electrochemical experiments at UMEs. Initially, we identify the conditions under which edge effects begin to significantly impact the particle-size estimation. By varying the UME size while using two fixed sizes of polystyrene (PS) beads, we established the conditions under which edge effects influence the current response associated with stochastic collision events. We then combine numerical simulations with experimental data to determine a semiempirical correction factor that accounts for the edge effect and improves the accuracy of particle-size determination. Finally, we evaluate the applicability and limitations of this semiempirical edge-flux correction factor across a range of UME and PS bead sizes. Our results demonstrate that the proposed correction significantly reduces the error in particle-size estimation, expanding the utility of stochastic electrochemistry for quantitative particle characterization.

## Graphical abstract

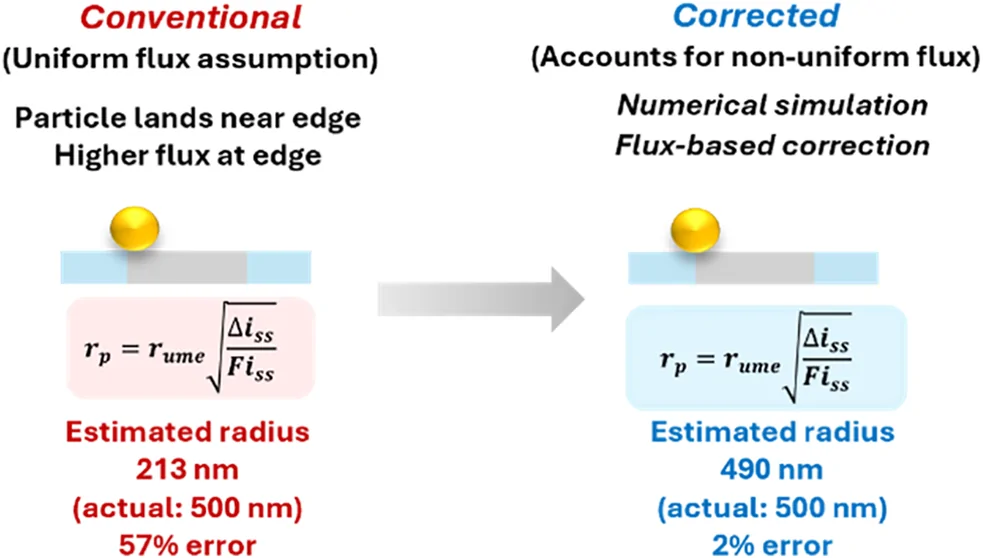

## Introduction

Ever since the invention of submicrometer and nanoelectrodes, the electrochemical community has been applying them to study heterogeneous electron transfer reactions, electrochemical sensors in biological samples, and ultimately for single-molecule detection.^1–5^ In 2004, Quin, van’t Hof, and Lemay found that insulating particles can be adsorbed onto ultramicroelectrode (UME) surfaces and block electron transfer from/to diffusing redox species.^6^ These investigations resulted in the observation of discrete events of these insulating particles at a single-molecule level. Later in 2007 and 2010, the first publications of conductive particles colliding on carbon and Pt ultramicroelectrodes via electrocatalytic amplification were published.^7,8^ Later, multiple studies were published on this topic spanning from magnetic particles, emulsion droplets, and insulating particles that were addressed via this collision strategy.^9–16^ These studies have also been extended to investigate electrocatalysis at the single-nanoparticle level.^17,18^ The principle is based on using very low nanoparticle concentrations, typically in the femtomolar (fM) range, so that individual insulating or conductive nanoparticles generate isolated current signals. These single-particle events can then be analyzed to determine physical properties such as the diffusion coefficient, particle size, and in the case for conductive nanoparticles, electron transfer rate constants.^19^ Consequently, this research topic was later denominated as stochastic electrochemistry with studies focusing at the single-particle level where individual particles are addressed and analyzed using ultramicroelectrodes. Since then, interesting results have been used to explore viruses, mammalian cells, bacteria, and droplets.^20,21^

However, even though it has been more than a decade since the demonstration of these multiple studies, there are still some limitations that prevent further application for electroanalysis, such as nanoparticle characterization.^22–24^ Some research has shown that proteins can be analyzed with this technique and achieve very good agreement between theoretical and experimental values.^25^ Nevertheless, for the investigation of polymeric particles, usually with diameters of hundreds of nanometers, these experiments failed to provide an accurate measurement of the particle size. This has been demonstrated to be due to the edge effect on UME’s.^23^ At such small electrodes, the diffusional flux does not behave homogeneously, and it has been demonstrated that the edges possess a higher flux compared to the center of the surface.^26^ This indicates the signals obtained during collision experiments depend on where they occur. Different current steps are therefore obtained depending on where a particle blocks the electrode surface because the local diffusional flux is position-dependent. Thus, the uncertainty in the measurements increases, failing to provide an accurate particle size. Until now, several studies have been published where this challenge is addressed and have exposed different strategies. For example, the use of a hemispherical UME instead of an inlaid disc demonstrated a better correlation between the experimental and expected size.^27^ The use of a Hg hemispherical UME demonstrated that edge effects do not alter the estimation of particle size in these measurements. However, a disadvantage of using a Hg UME is its toxicity and that it is easily oxidized. Nevertheless, it has been proven that utilizing a hemispherical or spherical UME eliminates any edge effect since diffusional flux is uniform at a sphere or hemisphere.^27^ Another study demonstrated that when eliminating the edge effect, nanoparticle collisions are a viable electroanalytical technique to determine the particle size when applying a potential close to the kinetically controlled region.^22^ By doing this, the electrochemical response is not governed by diffusion but by the electron transfer rate. Therefore, authors demonstrated better correlation when a potential near the kinetically controlled region produced better size determination of insulating particles.^22^ Similarly, it was demonstrated that edge effects can be mitigated by performing an electrocatalytic interruption, which translates to utilizing a homogenous chemical reaction, and this shifts the current generation from mass transport-limited to a chemical rate-limited. This electrocatalytic interruption method reducededge effects and was demonstrated to enhanced signals from smaller particles.^28^ Lastly, another interesting alternative to avoid edge effects in these estimations is by utilizing ring electrodes. A published study demonstrated that utilizing ring instead of inlaid disc UME’s resulted in a more uniform step size distribution.^29^

These edge effects are not unique to stochastic electrochemistry. In recent years, nanopore electrochemistry has emerged as an alternative technique for estimating particle sizing. This method considers the fabrication of a nanopore either through (1) nanofabrication techniques or (2) electrochemically dissolving a portion of a metal nanoelectrode to create a nanopore for electrochemical sensing.^30^ Similarly, nanopore-sized capillaries can be fabricated using a pipet puller to produce capillaries with nanometer-scale dimensions.^31^ These approaches provide alternative strategies for particle-size estimation. Previous studies have shown that when particles translocate through or interact with different locations within a nanopore, the measured signal varies, increasing the uncertainty in particle-size determination.^32,33^ These findings highlight the importance of edge effects in stochastic electrochemical sensing, where the measured response is strongly influenced by the position at which the particle interacts with the sensing interface.

Bonezzi and Boika introduced a model that helps investigators distinguish between agglomerates within a stochastic experiment. They also explained how the electrode size affects the current step generated from the collision of an insulating particle on the UME surface. This approach turns out to be very useful as it relates to how the concentration of the redox mediator, electrode, and analyte size contributes to the current step. With their model, one can approximate the current step by using the geometric factor, but it was unclear whether this geometric factor would work for different electrode sizes.^23^

Despite the tremendous efforts in this field, there are still many challenges that prevent the application of nanoparticle collisions as an electroanalytical technique to obtain quantitative information from the sample (e.g., particle size). Nanoparticle collisions are still used to extract quantitative information at the single-particle level in biological samples, electrocatalysis, etc.^34–40^ While previous studies established the influence of edge effects on blocking current magnitudes, it has been assumed that these edge effects are always present and prevent an accurate particle-size estimation.

In this study, we present several aspects that will provide insight into understanding how a more accurate size determination can be obtained by considering edge effects. We extended the understanding and described when edge effects become important in the particle-size determination. We derive a generalized correction expression that quantitatively compensates for edge-induced deviations in blocking current measurements, enabling a more accurate particle-size determination across a broad range of electrode-to-particle-size ratios. Here, we first performed experiments varying the electrode size and identified the electrode-to-particle-size regimes in which edge effects dominate the blocking response. We derived a semiempirical solution based on experimental data in combination with finite-element simulations to address the deviation when calculating the particle size. As a final determination, we applied this solution to different systems with different electrode sizes and particle sizes to test and validate the method.

## Materials and Methods

### Materials

All chemicals were of analytical grade and used as received. Potassium chloride (KCl) and calcium chloride (CaCl_2_) were purchased from Fisher Scientific. Ferrocenemethanol 98% was purchased from Ambeed, Inc. Ultrapure water (18.2 MΩ·cm) from an Integral3Milli-Q system (Millipore Co., Bedford, MA) was employed for working solution preparation. Polystyrene (PS) beads for this study were purchased from Polysciences, Inc. These PS beads were dispersed in solution and were used as received. Sizes used in this study were 4.50 μm, 3 μm, 1 μm, and 500 nm in diameter and certified by the manufacturer. Argon gas was ultrahigh purity grade. Platinum wires of 50, 25, and 10 μm were obtained from Goodfellow, Inc.

### Instrumentation

All electrochemical measurements were performed using a CHI 660C Instruments electrochemical workstation and a CHI202 Faraday cage.

### UME Fabrication

Briefly, for Pt electrodes with diameters of 50, 25, and 10 μm, a 1.5 cm long wire was cut and inserted into a borosilicate glass capillary (Sutter Instruments, 2.0 mm O.D. and 1.16 mm I.D.), with one end of the capillary closed using an oxygen-gas flame. The wire was then sealed using a heating coil, and an electrical connection was made using a copper wire and silver epoxy. The assembly was left in an oven overnight at 120 °C to cure the epoxy. Subsequently, the electrode was polished first with grit paper and then finely polished using an alumina slurry.^41^

For UMEs smaller than 10 μm, a different method was employed. A 10 μm wire was electrochemically etched in a 6 M CaCl_2_ solution by applying 5.5 V AC at 100 Hz, as described in previous studies. Once etched, the wire was inserted into a borosilicate glass capillary, and the same sealing and electrical connection procedure described above was followed. Using this approach, electrodes with diameters of 4, 3, and 1.8 μm were fabricated. The electrodes were polished to the desired diameter under an optical microscope, and their size was verified using steady-state voltammetry in 2.5 mM ferrocenemethanol with 0.1 M KCl.^30,42^ Steady-state voltammetry for each electrode is shown in Figures S1–S3.

### Nanoparticle Collision Experiments

The method for performing nanoparticle collision experiment has been well-established in the literature. A three-electrode electrochemical setup was used, with Ag/AgCl KCl saturated as references electrode, a Pt wire as counter electrode, and different Pt UMEs as working electrodes. Nanoparticle collision experiments were performed by applying a +0.4 V vs Ag/AgCl and recording for 400 s; after 20–30 s, an aliquot containing the PS beads was injected and immediately bubbled with Ar for 10–20 s. After this, the signals for every particle colliding were recorded with the Faraday cage closed. The solution used in these experiments was 2.5 mM FcMeOH +1 mM KCl. The present study focuses on the analysis of current steps and their accurate interpretation. Accordingly, the PS bead concentration for *a* = 1.50 μm and *a* = 2.25 μm was maintained below 5 fM to ensure the detection of isolated single-collision events and to prevent signal convolution that could compromise the analysis. Collision experiments at different supporting electrolyte concentrations were also performed. In this series of experiments, the KCl concentration varied between 1, 5, and 25 mM. The results are discussed in Figure S4.

### Data Analysis

The collision data were analyzed hierarchically at the collision and run levels to distinguish variability among individual collision events from variability among independent experimental runs. An independent experimental run is a fresh solution with the redox mediator and fresh particle aliquot; the electrode was polished between each run (*N*), and *n* denotes the total number of individual collision events collected across the runs. At the collision level, individual blocking collision events were identified and analyzed within each experimental run to obtain the individual current-step values. The mean current step and standard deviation (SD) calculated from the individual collision events were used to characterize the within-run variability. Histograms of the individual collision current-step distributions and cumulative-mean convergence analyses are provided in the Supporting Information.

At the run level, *N* independent collision experiments were analyzed for each UME. The mean current step from each run was used as the independent observation, and the mean current step across runs was calculated from these run-level means. The SD among the independent run means was used to characterize between-run variability. The standard error of the mean (SEM) was calculated from the between-run SD and the number of independent runs, where *N* is the number of independent experimental runs. The 95% confidence interval (CI) was calculated using the Student’s *t* distribution with *N*–1 degrees of freedom. Thus, individual collision events within the same run were not treated as independent experimental replicates when estimating run-level uncertainty.

The arithmetic mean was used as the central measure because the mean collision current step is the parameter used for subsequent particle-size calculation. The SD at the collision level describes event-to-event variability, whereas the SD, SEM, and 95% CI at the run level describe variability and uncertainty among the independent experimental runs. The individual collision-level distributions are provided in the Supporting Information to allow assessment of the distribution and dispersion of the measured current steps.

Cumulative-mean convergence analyses were performed to evaluate the stability of the collision current-step estimate as additional collision events were included. These analyses were used to assess the stability of the measurements rather than to define an arbitrary minimum number of collision events. For the 1.60 μm UME, the experimentally constrained collision frequency resulted in a limited number of collision events, and the available data was insufficient to establish robust convergence. Therefore, this condition was not used to make quantitative claims regarding model validity at this particle-to-electrode ratio but the numbers are still reported. To obtain the particle size from the electrochemical measurements, the run-level mean current step obtained for each UME was used in the particle-size calculation. The uncertainty in the derived particle size was propagated from the uncertainty in the corresponding run-level current-step measurement.

To test our correction factor, we utilized a different Pt UME with *a* = 5.90 μm, and the same data analysis was performed; first, we analyzed the variability of the current step within a collision experiment and then analyzed the variability within each run. Lastly, to test if the correction factor can be utilized with smaller UME and PS beads, we performed two collision experiments with both UMEs and PS beads. One group with a Pt UME with *a* = 2.02 μm and PS bead with *a* = 500 nm and the second group with *a* = 0.80 μm and PS bead with *a* = 250 nm. The analysis was performed the same as with the other experiments; first, we analyzed the variability of current steps within the collision experiment and then the variability between each run.

### Numerical Simulations

The description of the finite-element simulation (COMSOL 5.4) can be found in the Supporting information of this document. Briefly, we based our simulation model on previous studies, where an axisymmetric geometry was used to describe the flux toward a disk UME and estimate current steps of insulating particles.^23,43^ The model assumes that the current step can be obtained by considering a ring of PS beads and then using this relationship to estimate how many PS beads can fit into this ring. This allowed us to estimate the current steps at the edge and center for each individual PS bead. This was done for three different UME sizes, and the simulated current steps were extracted and compared to both experimental and theoretical values. Because ferrocenemethanol is a neutral species, mass transport occurs purely by diffusion, and migration effects were neglected. The convection effect was also neglected. Although the supporting electrolyte concentration is low, migration of the neutral redox species is inherently negligible and thus does not contribute to mass transport and ultimately does not impact the steady-state limiting current.

## Results and Discussion

### Validity of Current-Step Interpretation for Particle-Size Determination

The initial objective of this study was to systematically vary the electrode size and evaluate its influence on the measured current step for particle-size determination. Two PS bead radii (*a* = 1.50 μm and *a* = 2.25 μm) were investigated. The radius of an insulating particle can be estimated using eq 1

1 $$r_{{\Delta i}_{ss}}=r_{UME}\sqrt{\left(\frac{\Delta i_{ss}}{i_{ss}}\right)}$$

where *r*_Δ*i*_ss__ is the estimated particle radius (i.e., PS bead), *r*_UME_ is the radius of the UME, Δ*i*_ss_ is the current step, and *i*_ss_ is the steady-state limiting current of the UME in the presence of a redox mediator (e.g., ferrocenemethanol)..

The purpose of the experiment was to determine the conditions under which this relationship deviates, leading to discrepancies between the estimated and true particle sizes. We hypothesize the existence of a critical electrode size to particle-size threshold, governed by edge effects, below which eq 1 becomes invalid and above which accurate particle-size estimation converges with this equation. Figure 1 shows representative collision signals for PS beads on UMEs of different radii. For the smallest electrodes, current steps are readily observable (Figure 1c,d), consistent with the larger ratio of Δ*i*_ss_/*i*_ss_ when the particle size is comparable to the electrode radius. As the electrode radius increases relative to the particle size, the magnitude of the current step decreases, making detection more challenging. This effect is evident in Figure 1a,b for electrode radii of 29.40 μm and 13.70 μm, respectively, where current steps are only clearly resolved upon magnification of the current trace. An interesting observation is how the current step at electrodes with *a* = 29.40 μm and *a* = 13.70 μm had a spike before reaching steady-state current, whereas the electrode with *a* = 5.80 μm and 1.60 μm did not show this spike and was a flat current step. This is related to the reorganization of the diffusion layer around the particle impact at the UME–solution interface.^44^ Interestingly, in previous studies where blocking collisions have been performed utilizing yeast cells reported that when the UME radius approaches the particle size, the signal will not be a distinct step; instead, the signal will show a small blip response, similar to those found in droplet collisions.^45,46^ Our experiments with PS bead did not show such a response, and a distinct current step was observed as shown in Figure 1d. This contrasts to that reported by Lutkenhaus et al. with yeast cells.

**Figure 1:**
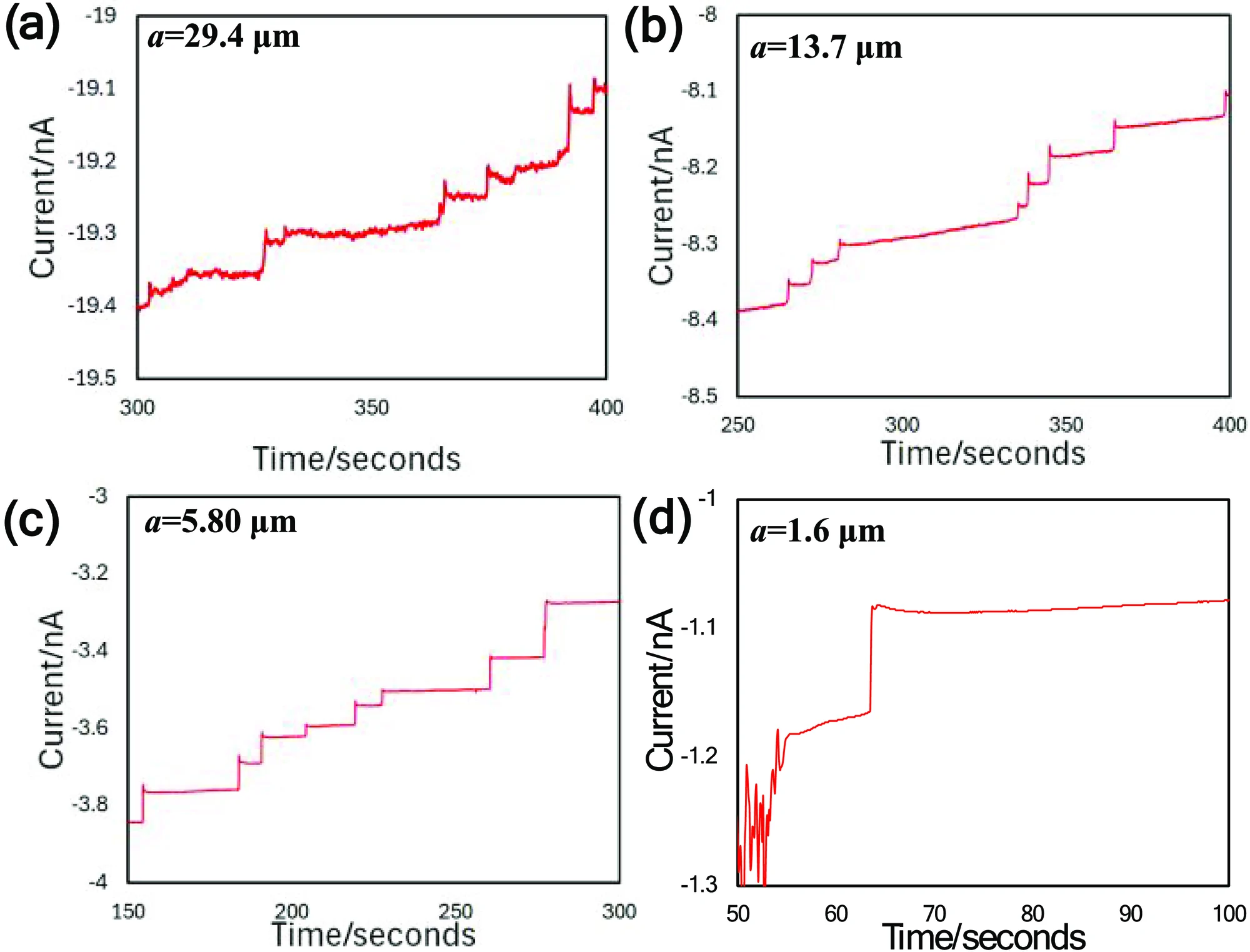
Collisions of PS bead with *a =* 1.50 μm on Pt UMEs with different radii. (a) Pt UME with *a =* 29.4 μm, PS bead concentration was 3 fM. (b) Pt UME with *a =* 13.7 μm, PS bead concentration was 4 fM. (c) Pt UME with *a =* 5.80 μm, PS bead concentration was 5 fM. (d) Pt UME with *a* = 1.6 μm, PS bead concentration was 4 fM. The testing solution was 2.5 mM FcMeOH + 1 mM KCl. The applied potential was +0.4 V vs Ag/AgCl (KCl sat’d). Time interval was 0.08 s.

Analysis of the current steps using eq 1 reveals that, for the largest electrode (*a* = 29.40 μm), the estimated PS bead radius 1.526 ± 0.065 μm agrees well with the manufacturer-specified value 1.500 ± 0.012 μm. However, as the electrode radius decreases, the estimated particle size deviates from the true value. For an electrode with *a* = 13.70 μm, the estimated radius is 1.073 ± 0.025 μm, while for *a* = 5.80 μm, it decreases further to 0.667 ± 0.034 μm. Ultimately, when an electrode approaches the particle size such as the UME in Figure 1d, the particle radius found was 0.431 ± 0.038 μm. Figures S5–S8 show collision and background current traces for comparison. Similarly, statistical data with the number of events and averaged current step for each experiment are shown: Table S1 shows the run-level variability, Table S2 shows the collision-level statistical values, and Table S3 shows the run-level particle-size calculation with eq 1. The histograms and convergence plots are shown in Figures S9 and S10, respectively.

To further validate this trend, experiments were repeated using larger PS beads (*a* = 2.25 μm) under identical electrochemical testing conditions. A similar pattern was observed. For the largest electrode (*a* = 29.40 μm), the estimated radius 2.031± 0.141 μm is in reasonable agreement with the manufacturer value 2.25 ± 0.16 μm. However, decreasing the electrode radius to 13.70 μm results in an estimated radius of 1.487 ± 0.03 μm, indicating clear deviation. For the electrode with *a* = 5.80 μm, the estimated radius further decreases to 1.023 ± 0.074 μm. In this case, the smallest electrode had *a* = 2.1 μm, which is comparable to the PS bead radius. The estimated radius with this UME was 0.879 ± 0.074 μm. The signal at the UME size was a distinct current step, suggesting the PS bead stuck on the UME surface (Figure 2d), which follows the trend found in the experiments with a PS bead with *a* = 1.5 μm. This size inconsistency has been published in previous reports, indicating that edge effects become more pronounced as the electrode radius decreases, thereby affecting mass transport and distorting the current response in nanoparticle collision experiments.^23,29,47^ These edge effects account for the increasing deviation from the true particle size at smaller electrode dimensions, which ultimately limits the application of nanoparticle collision experiments to determine the particle size. The statistical data for experiments with this PS bead is shown in Tables S4, S5, and S6, whereas histograms and convergence plots are shown in Tables S11 and S12 for PS beads with *a* = 2.25 μm.

**Figure 2:**
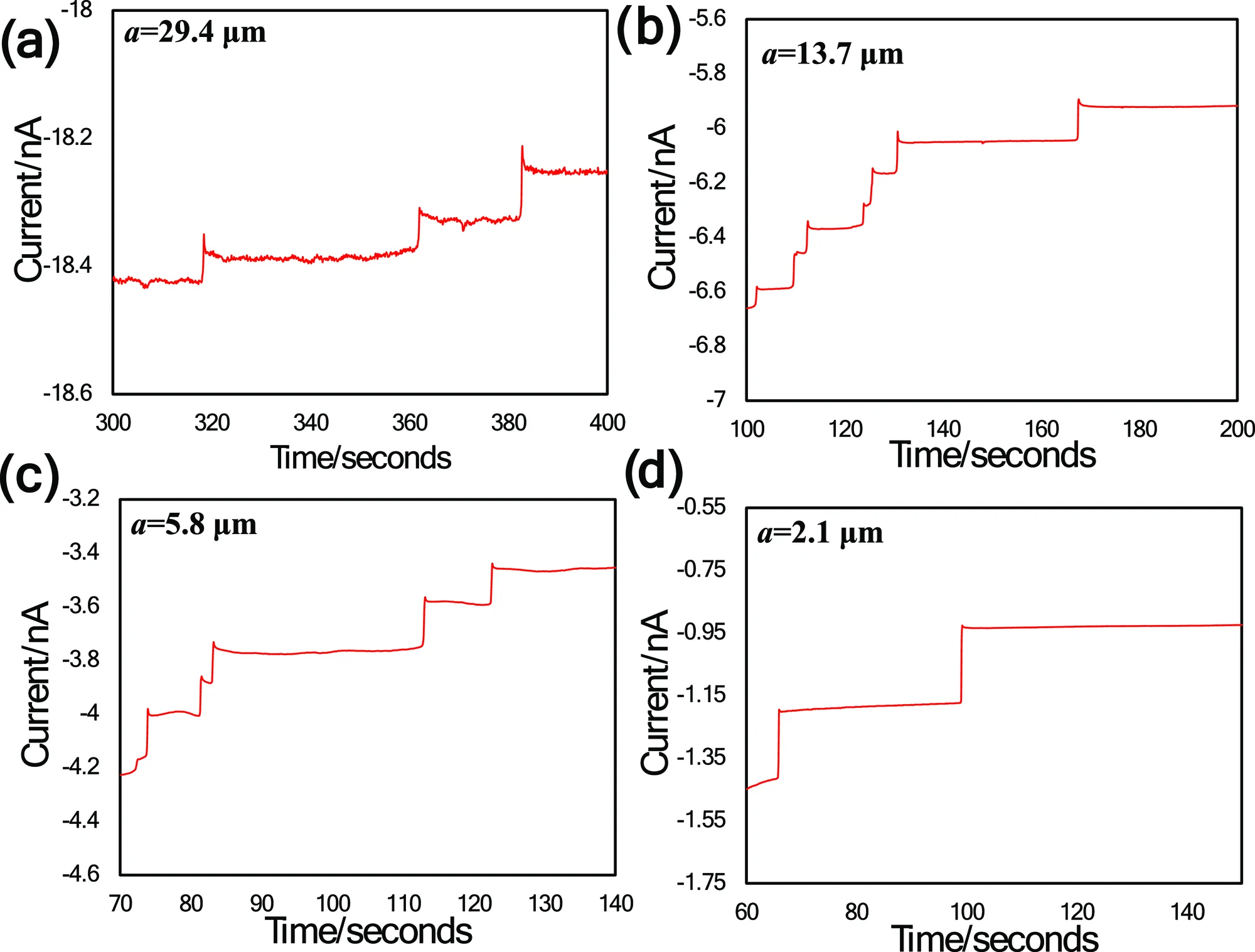
Collisions of PS bead with *a =* 2.25 μm on Pt UMEs with different radii. (a) Pt UME with *a =* 29.4 μm, PS bead concentration was 3 fM. (b) Pt UME with *a =* 13.7 μm, PS bead concentration was 4 fM. (c) Pt UME with *a =* 5.80 μm, PS bead concentration was 4 fM. (d) Pt UME with *a =* 2.10 μm, PS bead concentration was 5 fM. The testing solution was 2.5 mM FcMeOH + 1 mM KCl. The applied potential was +0.4 V vs Ag/AgCl (KCl sat’d). Time interval was 0.08 s.

Our results suggest that as the *r*_UME_/*r*_PS_ ratio decreases, eq 1 begins to diverge and fails to accurately estimate the particle size. This divergence is illustrated in Figure 3, where the relative percent error between the measured and true PS bead sizes, as well as the ratio *r*_measured_/*r*_true_, are plotted as a function of *r*_UME_/*r*_PS_. The values of *r*_UME_/*r*_PS_ for the different PS beads and UMEs are tabulated in Table 1. Notably, as shown in Figure 3a, the relative percent error decreases substantially when *r*_UME_/*r*_PS_ ≳ 13, suggesting that eq 1 converges and provides accurate particle-size estimates. This trend is further supported in Figure 3b, where the ratio *r*_measured_/*r*_true_ is plotted against *r*_UME_/*r*_PS_. As the electrode radius increases, this ratio approaches unity, indicating that eq 1 accurately predicts the PS bead size. However, as the electrode radius decreases, eq 1 progressively underestimates the particle size. This behavior is reflected by the increasing relative percent error in Figure 3a and the pronounced deviation of the *r*_measured_/*r*_true_ ratio from unity in Figure 3b at smaller *r*_UME_/*r*_PS_ values. The red dashed line denotes the practical transition between the accurate and underestimation regimes. To quantify this transition, each data set in Figure 3b was fitted with a power-law function, which provided the best description of the experimental trend. We then selected *r*_measured_/*r*_true_ = 0.9 (corresponding to a 10% error) as a practical benchmark for acceptable particle-size estimation. Using the fitted power-law relationships, we determined the value of *r*_UME_/*r*_PS_ at which *r*_measured_/*r*_true_ = 0.9, yielding a practical threshold of approximately 13. Below this value, eq 1 systematically underestimates the PS bead size, consistent with our experimental observations using smaller UMEs. A practical limitation of operating at larger *r*_UME_/*r*_PS_ values is that the magnitude of Δ*i*_ss_ decreases, making current-step detection more challenging. Nevertheless, as demonstrated here, these current steps remain measurable, allowing reliable particle-size estimation under these conditions.

**Figure 3:**
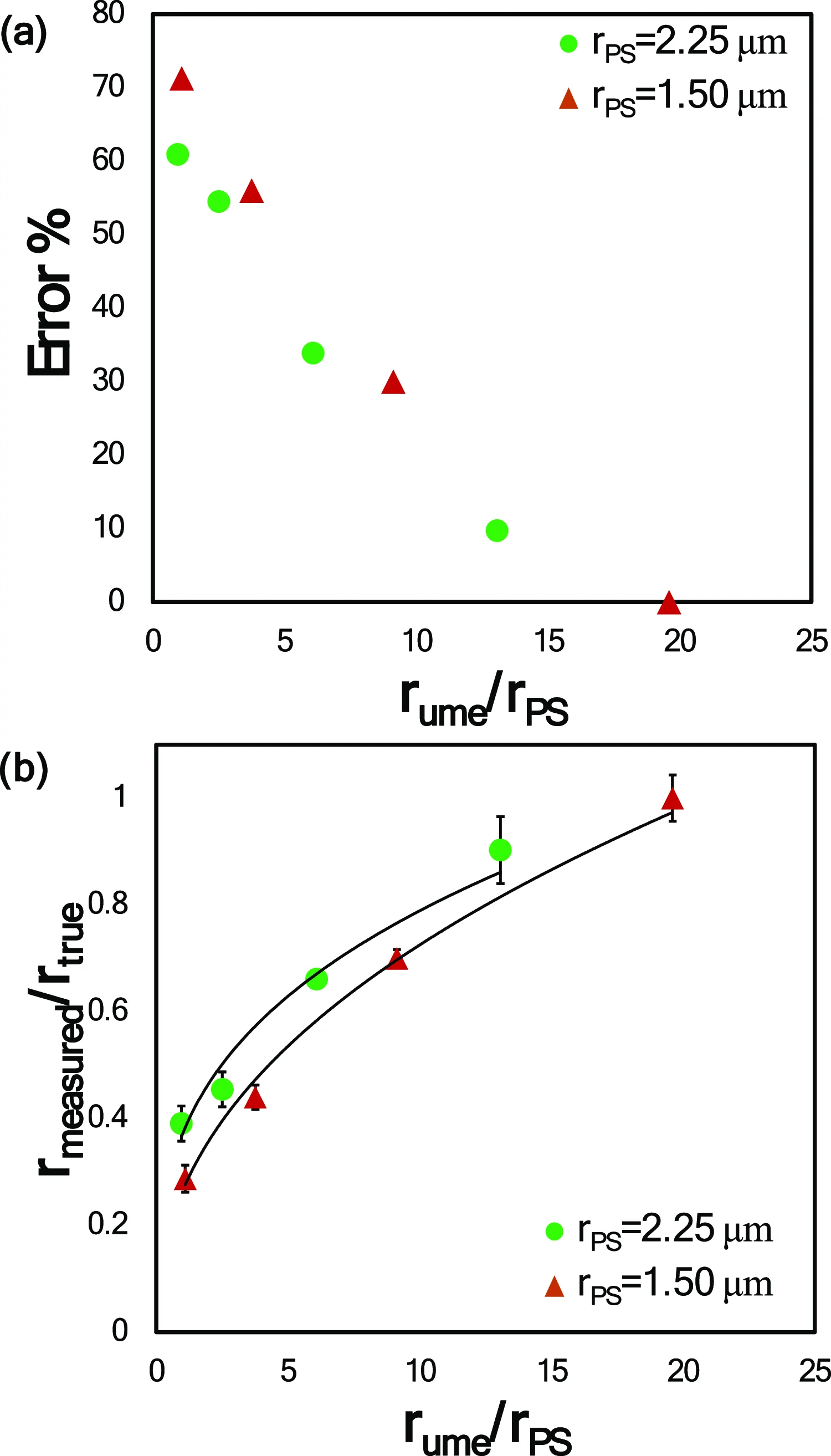
Utilization of eq 1 to estimate particle size valid at ratios above 13. Below this ratio, the error increases. (a) Relative error associated with the measured PS bead radius based on the true radius plotted as a function of *r*_ume_/*r*_PS_ for both PS bead sizes. (b) Ratio of r_measured_/r_true_ vs *r*_ume_/*r*_PS_ that exhibits a threshold (dashed line). Error bars were obtained from the standard deviation associated with the PS bead size calculated from the current steps during a collision experiment.

Overall, these results demonstrate that eq 1 provides reliable particle-size estimates when *r*_UME_/*r*_PS_ ≳ 13, whereas systematic underestimation occurs below this practical threshold. The agreement among the relative error analysis, the *r*_measured_/*r*_true_ ratio, and the fitted power-law relationships establishes this value as a practical criterion for the direct application of eq 1. In the following section, numerical simulations are used to investigate the physical origin of this transition and to develop a semiempirical edge-flux correction factor for measurements performed below this threshold.

### Numerical Simulations on Edge Effects and Electrode Sizes

Numerical simulations were conducted to elucidate how the current step is influenced by the particle landing position (i.e., edge vs center) and electrode size. Simulated results were compared with experimental data and theoretical predictions obtained from eq 1. The simulation geometry employed in this work was two-dimensional and axially symmetric. On the basis of prior studies, the current step was determined by simulating the flux of a redox species toward the UME. Specifically, the current step was obtained by comparing the steady-state limiting current in the absence and presence of a PS bead. To further refine the model, the current step associated with an individual PS bead was estimated by considering the number of PS beads that can geometrically fit within the UME ring, thereby improving the accuracy of the simulation, as described previously.^43^ Figures S13 and S14 show the geometry for the simulation and concentration profiles obtained for the computational work, respectively.

Using this approach, current steps were calculated for a PS bead with a radius of 1.50 μm and three-electrode radii (*a* = 1.6, 5.80, 13.7, and 29.4 μm) at two distinct locations: the center and the edge of the UME surface. These simulated values were compared with experimental data and theoretical predictions. The theoretical current steps were obtained by rearranging eq 1 to solve for Δ*i*_ss_ for each case.

To validate the simulated current steps, we simulate the current-step responses at different redox mediator concentrations, and they were compared to the theoretical current step for a specific UME size. Previous studies have shown that the redox mediator concentration and current step should scale linearly.^23,43^ For this to be validated, we simulated 3 different redox mediator concentrations as follows: 2.5, 5, and 7.5 mM. As shown in Figure S16, a linear trend was obtained as concentration increases for all three different responses. This result is expected because the concentration increases the steady-state limiting current, and therefore the magnitude of the current step will be affected. In this case, at higher concentrations of different redox mediators, the current step increases in all cases.

The results are presented in Figure 4, which compares simulated current steps at the edge and center, theoretical values, and experimental data for a PS bead with *a* = 1.50 μm. As the UME radius decreases, both simulated and theoretical current steps exhibit an exponential trend, whereas the experimental values deviate from this behavior. A clear difference is observed between edge- and center-based simulations, which arises from the nonuniform flux distribution at the UME surface. Specifically, the flux is higher near the electrode edge due to hemispherical diffusion, leading to larger current steps for edge-located particles. Despite this difference, both edge and center simulations follow a similar exponential dependence as *r*_UME_/*r*_Δ*i*_ss__ decreases.

**Figure 4:**
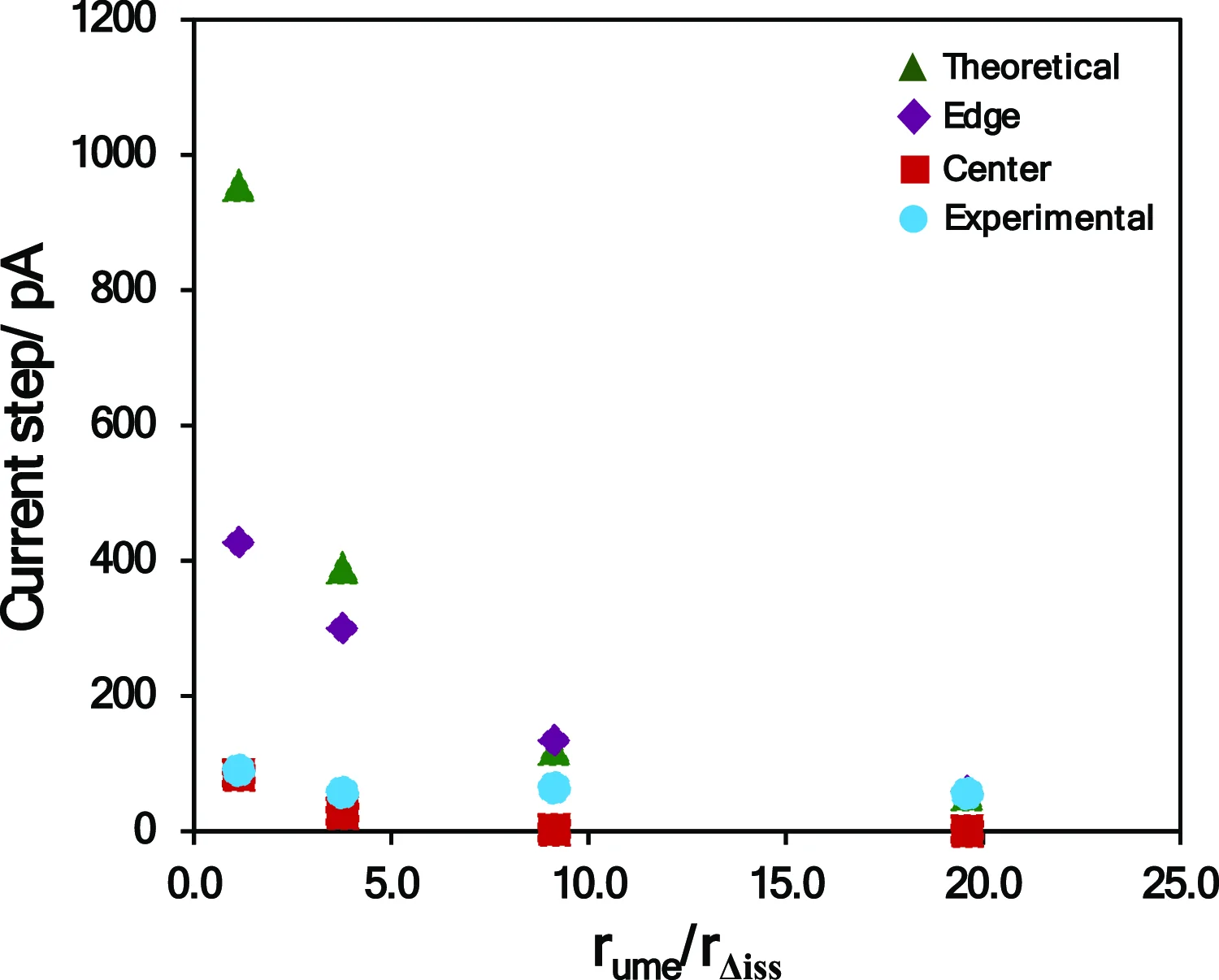
Comparison of current steps for experimental, simulated edge and center, and theoretical as a function of *r*_ume_/*r*_Δ*i*ss_. These results were obtained for a PS bead with *a =* 1.50 μm.

At larger electrode sizes (*r*_UME_/*r*_Δ*i*_ss__ ≳ 13), the edge-based simulated, theoretical, and experimental current steps converge, supporting the validity of eq 1 for accurately estimating PS bead size under these conditions. The error between experimental and theoretical values in this regime was approximately 2%. In contrast, the center-based contribution is negligible, indicating that the measured signal is dominated by edge-weighted flux contributions. This observation is consistent with previous studies, where edge-enhanced flux leads to a stronger electrochemical response for particle impacts near the electrode perimeter.^47^

However, as *r*_UME_/*r*_Δ*i*_ss__ decreases, eq 1 becomes increasingly inaccurate. In this regime, the theoretical prediction deviates from edge-based simulations and approaches the center-based response, particularly when *r*_UME_/*r*_Δ*i*_ss__ < 5, as shown in Figure 4 (blue circles). Under these conditions, eq 1 significantly underestimates the particle size, with errors reaching approximately 86%. This deviation arises because the measured current step reflects a spatially weighted convolution of flux contributions across the electrode surface, rather than a purely edge-dominated response or homogeneous flux. As a result, the stochastic landing position of insulating particles introduces variability that is not captured by eq 1.

Based on these results, two distinct regimes can be identified. When *r*_UME_/*r*_Δ*i*_ss__ ≳ 13, the current response is dominated by edge-enhanced flux, and eq 1 provides accurate particle-size estimates. In contrast, when *r*_UME_/*r*_Δ*i*_s*s*__ < 13, both edge and center contributions influence the measured current, leading to systematic deviations from theoretical predictions. As the particle size approaches that of the UME, the flux distribution becomes increasingly perturbed across the entire electrode surface, reducing the validity of edge-based assumptions and resulting in smaller measured current steps relative to theoretical values. Overall, these findings demonstrate that eq 1 accurately estimates the particle radius at specific *r*_UME_/*r*_Δ*i*_ss__ conditions (≳13) and highlights the need for a semiempirical edge-flux correction factor that accounts for spatial variations in flux. Interestingly, the experimental current-step response when a UME with *a* = 1.6 μm was utilized (*r*_UME_/*r*_Δ*i*_ss__ = 1.1) matches the simulated current response as shown in Figure 4 (red square and light blue circle). This suggests that under these conditions, the experimental response will mostly influence the center flux of the UME rather than the edge flux. Similarly, eq 1 does not accurately describe the particle size as mentioned in the previous section that shows the discrepancy between the particle size estimated with eq 1 and the manufacturers’ reported size.

### Analytical Expression and Validation

From the previous sections, we have shown evidence that eq 1 can be used to estimate the size of an insulating particle (i.e., PS bead) under specific conditions and that it fails when these conditions are not met. The final section of this study introduces a semiempirical edge-flux correction factor that extends the applicability of eq 1 to systems where edge effects produce systematic deviations from the ideal prediction (i.e., different electrode and PS bead sizes). This semiempirical edge-flux correction factor can be extracted from combined experimental and simulation data and applied to improve the particle-size estimation across different systems. Although this factor is not universal, we demonstrate in this section that it can be applied to different PS bead sizes and electrode radii. Ultimately, we test when this semiempirical edge-flux correction factor becomes invalid and no longer allows an accurate estimation of the particle size.

The correction is based on the nonuniform flux distribution at the UME surface. Radial diffusion enhances mass transport at the electrode perimeter, resulting in a higher flux at the edge compared to the center.^42^ Consequently, the magnitude of the current step depends on the landing position of the insulating particle: particles landing near the edge produce larger current steps, whereas those landing near the center produce smaller responses. Under most experimental conditions, the measured current step reflects a combination of these two limiting cases.^23^

Our approach introduces a semiempirical edge-flux correction factor that accounts for the relative contributions of edge- and center-limited current responses arising from the nonuniform flux distribution. This is because the flux is not uniform and is higher at the edge compared with the center. The correction factor is based on the nonuniform radial flux distribution at the UME surface rather than on preferential particle landing. Particles are expected to collide across the entire electrode surface, with no preferred landing location. However, because the diffusional flux is highest near the electrode perimeter, collisions occurring close to the edge contribute disproportionately to the measured blocking current. Consequently, the experimentally observed current represents a flux-weighted average of the collisions occurring over the entire electrode surface.

When *r*_UME_/*r*_PS_ ≳ 13, the particle blocks only a small fraction of the electrode surface. Under these conditions, the spatially averaged blocking current closely follows the prediction of eq 1 because the contribution of the edge-weighted flux is appropriately represented in the overall response. As *r*_UME_/*r*_PS_ decreases, the particle blocks an increasingly larger fraction of the electrode surface, reducing the dominance of edge-enhanced diffusion and causing the measured current to become an average of both edge- and center-weighted flux contributions. This transition explains why eq 1 progressively underestimates the particle size. These events were observed in our results discussed in the first section of this study (Figures 1 and 2). Furthermore, it was demonstrated with the numerical simulations presented in Figure 4, where *r*_UME_/*r*_Δ*i*_ss__ ≳ 13, that the simulated edge, theoretical prediction, and experimental data match. However, when *r*_UME_/*r*_Δ*i*_ss__ < 13, eq 1 does not predict the particle size accurately. Therefore, we can calculate a weighing factor using our experimental data, simulated edge, and center current-step responses using the following expression

2 $$P=\frac{{\Delta i}_{\exp}-{\Delta i}_{center}}{{\Delta i}_{edge}-{\Delta i}_{center}}$$

where Δ*i*_exp_, Δ*i*_edge_, and Δ*i*_center_ correspond to the experimental, simulated edge, and simulated center current steps. *P* is a normalized interpolation parameter that locates the experimentally measured current between the limiting center and edge responses obtained from the numerical simulations. It should not be interpreted as the probability that a particle lands near the electrode edge. Instead, *P* quantifies how strongly the experimentally measured current resembles the limiting edge response relative to the limiting center response predicted by the simulations. This *P* value can later be used to calculate a semiempirical edge-flux correction factor (*F*), which compensates for measurement bias due to spatial flux heterogeneity. Although *F* is obtained from experimental measurements and numerical simulations, it is physically motivated by the nonuniform diffusional flux at the UME surface rather than being an arbitrary empirical fitting parameter. Because the measured current step reflects a weighted contribution of edge- and center-dominated events, *F* compensates for the resulting measurement bias under conditions where the assumptions underlying eq 1 are no longer adequately satisfied. *F* is subsequently incorporated into eq 1 to improve particle-size estimation under conditions where both edge- and center-limited flux contributions influence the measured current step. The following expression assumes that the measured current step can be approximated as a linear combination of edge and center responses. This approximation is justified because the experimental relationship between the normalized response and the geometric ratio remained linear throughout the range investigated

3 F=P+(1−P)β

where β *=* Δ*i*_center_/Δ*i*_edge_ is the ratio of the limiting center and edge current responses obtained from the numerical simulations. This β has values of 0 < β > 1. Smaller β values indicate a greater disparity between center and edge responses, whereas values approaching unity indicate a more uniform current response across the electrode surface. As the particle and UME dimensions become comparable, center-weighted flux contributions increasingly influence the measured current step, which is when *r*_UME_/*r*_Δ*i*_ss__ < 13. This criterion was established based on our experimental observations that were found under the conditions established in this study. The complete derivation of eq 3 is added to the Supporting information for more details. The derivation assumes that the experimentally measured current can be represented as a linear interpolation between the limiting center and edge responses predicted by the finite-element simulations. When *P* is substituted into eq 3 and used to calculate *F*, it provides a correction for the nonuniform flux distribution at the UME surface under the conditions of *r*_UME_/*r*_Δ*i*_ss__ < 13. Although radial flux distribution at the UME surface is inherently nonuniform, the experimentally observed relationship between *r*_UME_/*r*_p_ and the normalized response parameters (*P*, β) remains linear within the investigated electrode-to-particle-size ratios of this study. This suggests that the effects of spatial flux heterogeneity are effectively captured through the definitions of *P* and β, leading to an emergent linear mapping between geometric ratios and the measured electrochemical response. Consequently, introducing additional nonlinear weighting (e.g., exponent α) does not improve predictive accuracy and instead increases variance in the particle-size estimation. Figures showing this linearity are shown in Figure S17. The linear interpolation employed in eq 3 is not intended to represent the exact spatial distribution of diffusional flux but rather an effective description of the experimentally observed blocking current over the investigated range of *r*_UME_/*r*_PS_.

With *F* calculated, this can now be added to eq 1 as follows

4 $$r_{{\Delta i}_{ss}}=r_{UME}\sqrt{\left(\frac{\Delta i_{ss}}{{\mathrm{Fi}}_{ss}}\right)}$$

Once *F* is added, it will now provide a better estimation for the particle size based on the current step. It is important to mention that this will only be applicable when *r*_UME_/*r*_Δ*i*_ss__ < 13, because below this ratio, eq 1 underestimates the particle size, but for *r*_UME_/*r*_Δ*i*_ss__ ≳ 13, eq 1 is valid. Because the experimentally measured current is systematically reduced relative to the idealized prediction as the particle-to-electrode size ratio decreases, *F* is introduced in the denominator of eq 4 so that the effective normalized blocking current reflects the experimentally observed flux heterogeneity. This formulation preserves the original structure of eq 1 while compensating for the systematic underestimation identified in the previous sections.

To test the validity of this correction, we used our data from previous sections (PS bead size *a* = 1.5 μm) and attempted to estimate the PS bead size using this *F* value and subsequently with smaller UME and PS beads. First, a *P* value was calculated using eq 2 and then calculating *F* with eq 3, and ultimately the size of the PS bead. First, utilizing experimental data, we estimate a *P* value for each UME we used in the study. The results are shown in Table 2, where the current steps (edge, center, and experimental) were used to calculate *P* values and ultimately estimate the *F* value. After obtaining these values, *F* values were then used in eq 4 to estimate the PS bead size. When a UME with *a =* 5.80 μm, the average PS bead size was 0.667 ± 0.034 μm (without correction), which shows a 56% error from the PS bead size from the manufacturer (*a* = 1.5 μm). After utilizing eq 4 and *F* value from Table 2, the PS bead size was estimated to be 1.531 ± 0.076 μm, which reduces the relative error to 2.0%. As shown, when eq 4 is used with the *F* value for this electrode, it reduces the error significantly and estimates the PS bead size more accurately than eq 1.

We then estimate the particle size of the data obtained with a UME with *a =* 13.7 μm. When utilizing the *F* = 0.46 value from Table 2, we found an average PS bead size of 1.574 ± 0.076 μm, which remarkably agrees with the manufacturer PS bead size. The error in this analysis was 4.9%. As shown, when the calculation accounts for the heterogeneity of the current response (edge vs center), the experimental value agrees and provides a more accurate measurement of the PS bead size. Lastly, although for the Pt UME with *a* = 29.4 μm, eq 1 already estimates a particle radius in agreement with the manufacturer, when testing the *F* value for this electrode, the estimated particle radius is 1.568 ± 0.066 μm, which is still in agreement with the manufacturer’s reported size; however, under these conditions, as shown in the previous section, edge effects are not impacting the particle-size estimation compared to when the UME radius decreases.

Finally, we found that applying *F* = 0.210 to correct the particle size for experiments where *r*_UME_/*r*_Δ*i*_ss__ ≈ 1 did not improve the accuracy of the particle-size prediction. After applying this correction factor, the predicted particle radius was 1.0 ± 0.1 μm, corresponding to a 33% difference from the true value. We also evaluated *F* = 0.460, which further increased the difference to 58%, yielding a predicted particle radius of 0.62 ± 0.07 μm. These results suggest that the proposed correction factors are not applicable when the particle size is comparable to the UME radius. In addition, the low collision frequency observed for the smallest UME–particle combination (0.008 ± 0.002 s^–1^) resulted in a limited number of collision events. Consequently, although a particle-size estimate could be obtained, the available number of events does not provide sufficient statistical precision to establish a robust estimate for this condition. We therefore identify this UME–particle combination as a lower experimental limit of the proposed correction framework and do not use this condition to make quantitative claims regarding model validity at this particle-to-electrode ratio. The run-level particle-size estimates obtained using eq 4 for the different UME sizes are summarized in Table S7.

To test whether this equation and *F* values can be used in other experiments, we utilized another independent experiment with a Pt UME with *a =* 5.9 μm and same PS bead size (*a =* 1.50 μm); for this experiment, 3 runs were performed and analyzed as in the previous experiments. The run-level current-step mean and variability was 58.04 ± 6.1 pA (SEM = 3.52 pA, 95% CI = 50.47–65.6 pA), which falls within statistical variation with the results with a Pt UME of *a* = 5.80 μm. The number of total collisions in the three runs was 57 events. Applying eq 4 and *F* value of 0.19, we estimated a PS bead radius of 1.57 ± 0.083 μm, which agrees with the manufacturer PS bead radius with a relative error of 6.8% as opposed to the value obtained with no correction factor included; 0.68 ± 0.036 μm with a relative error of 55%. Table S8 shows the comparison between the corrected and uncorrected values for this data set.

To test whether this analysis is valid across different Pt UMEs and PS bead sizes, we performed two experiments utilizing (1) Pt UMEs with *a =* 2.04 μm and a PS bead size of *a =* 500 nm (*N* = 2) and (2) another with a smaller Pt UME with *a =* 0.80 μm and PS bead size of *a =* 250 nm (*N* = 2). As in both cases, *r*_ume_ */r*_PS_ ≈ 4 to the radius of the UME, we decided to focus on utilizing *F* = 0.190, which accounts for such a condition. Figure 5 shows the results of the collision experiments for both experiments. The PS bead concentration was maintained low to obtain distinct discrete current steps and avoid agglomeration of PS beads bouncing over the UME surface.^48^

**Figure 5:**
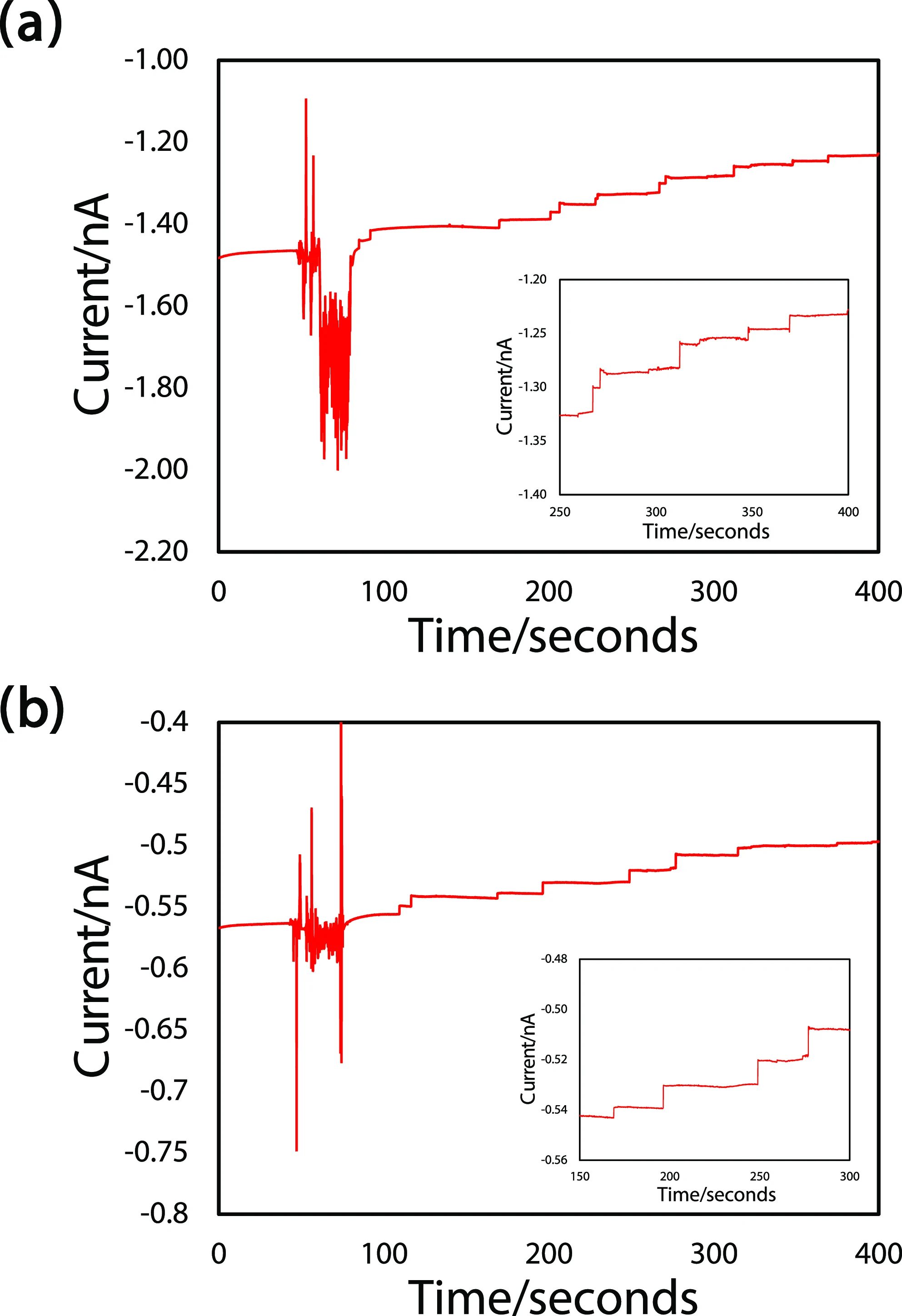
Collision data of PS beads with different sizes using smaller Pt UME. (a) With UME *a =* 2.01 μm and PS bead *a =* 500 nm. Concentration of PS beads was 5 fM. (b) With UME *a* = 800 nm and PS bead *a* = 250 nm. Concentration of PS beads was 15 fM. The testing solution was 2.5 mM FcMeOH + 1 mM KCl. The applied potential was +0.4 V vs Ag/AgCl (KCl sat’d). Time interval was 0.08 s.

When analyzing the current steps for the PS bead size with eq 1, as depicted in Figure 5a, we found at the run level the average current step to be 16.63 ± 0.53 pA (SEM = 0.38 pA, 95% CI = 11.86-21.3 pA, *N* = 2) and the PS bead size of 213 ± 4 nm, which has a 57% error compared to the value of the manufacturer. When eq 4 with *F* = 0.190 was used, the PS bead size was 490 ± 9 nm, which now has an error of 2 %, which drastically improves the estimation of the PS bead size; the comparisons are shown in Table S9. Because the particle radius becomes comparable to the electrode radius, the blocked area encompasses a larger fraction of the nonuniform diffusional field. Consequently, the measured blocking current reflects a broader spatial average of the surface flux distribution rather than being adequately described by the limiting edge response alone. It is worth mentioning that for this case, no simulations were performed, and we simply used the *F* value obtained with a larger UME and PS bead and applied it to these different conditions. The fact that this *F* value can be used across different UME and PS bead sizes and produces a significant improvement in the estimation of the PS bead size suggests potential transferability within similar insulating polymer systems under comparable electrochemical conditions.

We performed a similar comparison for the other experiment where a smaller Pt UME *a =* 800 nm and PS beads with *a =* 250 nm were used, as shown in Figure 5b. In this case, we kept *F* = 0.190 for eq 4 and we estimated a PS bead radius of 223 ± 4 nm, which is now closer to the manufacturer-reported size. The run-level mean current step was 8.364 ± 0.414 pA (SEM = 0.293 pA, 95% CI = 4.64–12.09 pA, *N* = 2). This result gave a relative error of 10%. These results were compared to the value obtained with eq 1, which was 97 ± 2 nm and has a 61% error and are shown in Table S10. As can be seen, this *F* value was used in two different Pt UME/PS bead size conditions and demonstrated to improve the estimation for PS bead sizes.

We then tested *F* value of 0.460; the error drastically increased from 2% to 37% for the results using a Pt UME with *a =* 2.04 μm and PS bead size of *a =* 500 nm. A similar case was found for the other system with Pt UME *a =* 800 nm and PS bead size of *a =* 250 nm. The error increased from 10% to 71%. This suggests that this *F* value of 0.460 does not account for the underestimation of eq 1. With this comparison of *F* values, we identified two conditions beside *r*_UME_/*r*_Δ*i*_ss__ ≳ 13 and *r*_UME_/*r*_Δ*i*_ss__ < 13. These conditions can be refined furthermore: *F* = 0.190 should only be used when 1 < *r*_UME_/*r*_Δ*i*_ss__ < 5, which is when the bead (e.g., polymeric) radius is comparable to the UME radius. It has been demonstrated that under these conditions, the current steps are well-resolved and are not affected by the background current.^43^ The value of *F* = 0.460 should only be used with the conditions of 5 < *r*_UME_/*r*_Δ*i*_ss__ < 13. However, it is worth mentioning that *F* is a semiempirical edge-flux correction factor, not a universal constant, and it needs to be tested with other samples such as biological samples to estimate whether this factor will be valid under such experimental conditions. For systems where soft polymeric particles and smaller UMEs’ are used (e.g., PS), this semiempirical edge-flux correction factor could be applied but only when the conditions are met such as 1 < *r*_ume_ */r*_PS_ < 5 or 5 < *r*_UME_/*r*_Δ*i*_ss__ < 13.

Bonezzi and Boika published a theoretical framework that accounts for edge effects and improves the accuracy of the predicted current step.^23^ We discussed their results with this for contrasting each work and offer the reader a broader perspective. A distinction from this study is that ours is applicable to estimate the particle radius, rather than the current step. Another main distinction between our study and theirs is our finding that edge-effect corrections are unnecessary when *r*_UME_/*r*_Δ*i*_ss__ ≳ 13. As demonstrated in the first section of this work, particle-size estimates obtained under these conditions agree well with the manufacturer-reported sizes of the PS beads used in this study (e.g., 3 and 4.5 μm diameter PS beads measured with 29.4 μm diameter UMEs). In contrast, Table 1 of Bonezzi and Boika’s work reports a 32% difference between the theoretical and experimental current-step heights for electrodes with a diameter of 22 μm. Our study provides a semiempirical correction factor for experimental conditions similar to these. Their study did not report any experimental data at electrodes larger than 22 μm.

**Table 1:** Values of *r*_ume_/*r*_PS_ Obtained from Experimental Data Shown in Figures 1 and 2

*r*_ume_ (μm)	*r*_ume_/*r*_PS_ *a* = 2.25 (μm)	*r*_ume_/*r*_PS_ *a =* 1.50 (μm)
29.4	13.06	19.6
13.7	9.13	6.09
5.80	3.86	2.51
2.1	0.93	N/A
1.6	N/A	1.1

To compare the two approaches, we applied their geometric correction factor to our experimental data. Bonezzi and Boika reported a geometric factor of *f*_*g*_ = 0.205, which they suggested could be applied over a broad range of electrode sizes. For our experimental conditions *a* = 13.7 μm and *r*_PS_ *=* 1.5 μm, the average experimental current step was 61.97 ± 2.20 pA. Using eq 1, the corresponding theoretical value of Δ*i*_ss_ is 122 pA, approximately twice the experimental current step. Applying *f*_*g*_ = 0.205 predicts a current step of only 25 pA, which is substantially smaller than both the experimental and theoretical values. In contrast, using the correction factor derived in this work for the same conditions (*F* = 0.460) yields a predicted current step of 57 pA, which is closer with the experimental value within the experimental uncertainty. For conditions where *r*_UME_/*r*_PS_ ≈ 4, our correction factor of *F =* 0.190 produces similar results to *f*_*g*_ = 0.205.

An important contribution of the present study is the identification of the conditions under which an edge-effect correction is required. This distinction was not clearly established in the work of Bonezzi and Boika, where *f*_*g*_ was described as an average value obtained by calculating the current step from the edge to the center of the UME. Also, a distinction with our work is that we applied our correction factor to a system with nanometer PS beads (*a* = 250 nm) and demonstrated that our *F* value of 0.190 is valid at different UME and PS bead sizes.

Furthermore, Bonezzi and Boika applied their correction factor to interpret the results reported by Dick et al., who used blocking collisions to characterize protein sizes with nanoelectrodes.^25^ It is therefore important to evaluate whether our semiempirical correction factors are also applicable to other systems reported in the literature and to compare their performance with previously proposed correction methods.

In their discussion of the work by Dick et al., Bonezzi and Boika applied *f*_*g*_ to experiments involving polystyrene beads with a radius of 11 ± 1 nm measured using a nanoelectrode with a radius of 140 nm and concluded the current step to be 21 ± 4 pA, which will result to be smaller than that reported by Dick et al.: 30 ± 7 pA. Under these conditions, 5 < *r*_UME_/*r*_Δ*i*_ss__ ≤ 13, corresponding to the regime in which our correction factor of *F* = 0.460 is applicable (Table 2). Applying this correction factor predicts a particle radius of 9 nm, which closely approximates the manufacturer’s reported value of 11 ± 1 nm.

**Table 2:** Data Used to Estimate *F* Values That Were Used in Eq 4 to Estimate the Particle Size

UME radius (μm)	Δ*i*_edge_ (pA)	Δ*i*_center_ (pA)	*X̅*Δ*i*_exp_ (pA)	*P*	β	*F*
1.6	427	100	91.97	–0.02456	0.234	0.215
5.80	310	33	57.5	0.1	0.106	0.190
13.7	135	1.76	61.97	0.452796	0.0130	0.460
29.4	58.9	0.454	56.48	0.950381	0.0077	0.951

Another example from Dick et al. further illustrates the importance of the *r*_UME_/*r*_Δ*i*_ss__ ratio. It is when they used glucose oxidase as the analyte (*a* = 4 nm) and an electrode with a radius of 120 nm. In their reported experiments, eq 1 accurately predicts the protein size without applying any correction factor. The measured current step was 18 ± 2.3 pA. Using this value together with the limiting current for a 120 nm radius electrode in 400 mM ferrocyanide yields an estimated protein radius of 4.3 nm, in excellent agreement with the reported protein radius of 4 nm. In contrast, Bonezzi and Boika attributed the discrepancy between their theoretical prediction and the experimental current step to protein agglomeration in the presence of the redox mediator, predicting a current step of only 3.3 pA rather than the experimentally observed 18 pA.

This example highlights the importance of considering the *r*_UME_/*r*_Δ*i*_ss__ ratio when determining whether an edge-effect correction is necessary. In this case, the ratio is approximately 30, well above the threshold value of 13 identified in the present work, indicating that no edge-effect correction is required. Although the uncorrected model accurately predicts the protein size in this example, it should be noted that protein stability and aggregation can strongly influence experimental measurements, as discussed by Bonezzi and Boika.

Overall, this comparison helps clarify the appropriate application of the correction factors proposed in this work. Specifically, when *r*_UME_/*r*_Δ*i*_ss__ ≳ 13, edge effects are negligible and no correction is required. Edge-effect corrections become important only as the particle size approaches that of the UME. We found a limitation in our proposed work when *r*_UME_/*r*_Δ*i*_ss__ ≈ 1. The proposed correction factors in this work (*F =* 0.46 and 0.190) are no longer valid and cannot accurately determine the particle size. Therefore, we conclude that the semiempirical correction factors developed here should be applied only within the range 1 < *r*_UME_/*r*_Δ*i*_ss__ < 13.

On the other hand, Bonezzi and Boika reported a correction factor of 0.081 for conditions where *r*_UME_/*r*_Δ*i*_*ss*__ ≈ 1. We applied this geometric factor to our experimental data for *r*_PS_ *=* 1.5 μm and *r*_ume_ *=* 1.6 μm. The run-level mean current step was 91.97 ± 16.11 pA and PS bead size was 1.44 ± 0.131 μm, which agrees with the manufacturer’s reported particle size within the associated uncertainty. In contrast, our correction factor of *F* = 0.215 does not accurately estimate the particle size when the particle and UME are of comparable dimensions, as it was estimated to be 0.914 ± 0.083 μm. This demonstrates the limitation of our model under these conditions. Interestingly, when *r*_UME_/*r*_Δ*i*_ss__ < 1, as in the experiment reported in this study using *r*_PS_ *=* 2.25 μm and *r*_ume_ *=* 2.1 μm, neither correction factor accurately predicts the particle size. Specifically, our correction factor of *F* = 0.190 fails to estimate the correct particle size, and the correction factor of 0.081 proposed by Bonezzi and Boika also fails to accurately predict either the particle size or the current step. We recognize that the smallest UME–particle condition is subject to an intrinsic physical limitation: as the particle-to-electrode size ratio approaches unity, the probability of observing collision events decreases, resulting in a low number of available collision events. This limitation is reflected in the statistical analysis through increased uncertainty in the estimated mean current step and derived particle size. Therefore, although the corrected particle-size estimate is reported, we do not interpret this condition as providing high-precision quantitative validation of the correction framework. Instead, we identify it as an experimentally constrained lower limit of the present approach.

Lutkenhaus et al. reported a study exploring the interpretation of blocking collision signals of yeast cells as a function of electrode radius.^45^ Our results are in partial agreement with those reported in this study. For larger electrodes, eq 1 successfully predicts the particle size. Consistent with these findings, both our experimental and simulated data show that eq 1 accurately predicts the particle size for electrodes with *a* = 29.4 μm for both PS bead sizes.

However, our results differ when smaller UMEs are used, as the particle size predicted by eq 1 is significantly smaller than the true particle size. Lutkenhaus et al. suggested that edge effects become negligible when *r*_UME_/*r*_Δ*i*_ss__ ≈ 1 and proposed a “magic ratio” of 0.67 (*r*_UME_/*r*_Δ*i*_ss__ ≈ 1.4). They supported this conclusion using finite-element simulations and by comparing their results with the *f*_*g*_ values reported by Bonezzi and Boika.^23^ They proposed that, at a ratio of 0.67, the correction factor converges for both edge and center collision responses, allowing eq 1 to accurately predict both the current step and the particle size.

In contrast, our results indicate that, for PS beads, a particle-to-electrode size ratio of ≳13 is required for eq 1 to accurately determine the particle size. Although the current steps become smaller at these larger ratios, they remain clearly distinguishable from the baseline and produce well-defined current steps, as shown in Figures 1a and 2a. Based on our experimental and simulated results, we propose that edge effects are still present at ratios ≳ 13; however, the perturbation of the diffusion layer by the PS bead becomes sufficiently small that its influence on the measured current step is minimized, and as already mentioned, correction factors are only necessary when performing measurements at conditions where UME and particle sizes are comparable.

The correction approach presented here is currently valid only for inlaid disk electrodes embedded in glass with RG > 10. It is also limited to spherical insulating particles such as polystyrene. We are currently investigating whether this correction factor can be extrapolated to other systems. For example, applying our correction empirical factor to the data reported by Dick et al. suggests that it may also be applicable to other types of particles, such as proteins and potentially another soft particle, including polypropylene.

Lee et al. reported blocking collision experiments using polypropylene microparticles with a diameter of 0.90 μm and a Pt UME with a radius of *a* = 5 μm.^49^ By analyzing the reported figures and experimental data, we estimated a current step of approximately 2 nA for the polypropylene particles and a limiting current of 132 nA under the reported experimental conditions. Applying our correction empirical factor (*F* = 0.460), yielded an estimated particle diameter of 0.91 μm, in excellent agreement to the values reported from SEM and DLS measurements. These results are encouraging and therefore suggest that the proposed correction factor may be applicable beyond polystyrene particles. More importantly, they further support the conclusion that the *r*_UME_/*r*_Δ*i*_ss__ ratio governs when edge effects become significant for particle sizing by blocking stochastic collisions.

Ultimately, after accounting for edge effects and understanding the limitations in determining the particle size, blocking collisions remain a valuable electrochemical technique compared to other particle-sensing methods. For example, nanopore stochastic electrochemistry and Coulter-counter methods are also widely used for particle sensing.^31–33,50^ A key limitation of blocking collisions is electrode fouling, as the technique depends on particles adhering to the electrode surface. As particles accumulate, the electrode surface becomes increasingly blocked, leading to fouling and an eventual need to replace the electrode. In contrast, nanopore sensing and Coulter-counter methods rely on the fabrication of nanometer-scale channels.^50^ Nevertheless, these techniques are also well-established technologies for particle sizing. Importantly, Coulter-counter-based devices are widely used for analyzing biological samples such as mammalian cells.^51^

## Conclusion

This work demonstrated several aspects of stochastic electrochemistry that remain challenging, particularly those involving edge effects at disk-shaped UMEs. The nonuniform diffusional flux associated with edge effects governs the magnitude of the blocking current and therefore limits accurate particle-size estimation when eq 1 is applied without correction. In this study, we derived the conditions under which particle collisions can be used to accurately estimate the particle size based on the observed current step for the first time. We examined the relationship between particle size and UME size, and then we evaluated how this relationship affects the estimation of polymeric particle sizes (e.g., PS particles). As demonstrated in this work, these experiments can be successfully used to estimate the sizes of spherical particles when the edge effect is properly accounted for.

This study establishes a practical criterion for applying eq 1 directly: when *r*_UME_/*r*_PS_ ≳ 13, particle size can be estimated within approximately 10% error without additional correction. Below this threshold, systematic underestimation occurs because of edge effects, and the correction factor developed here substantially improves the accuracy of the estimated particle size. We employed numerical simulations together with experimental measurements to derive a flux-weighted correction factor that compensates for the spatial heterogeneity of the diffusional flux at the UME surface. Our analysis yielded a factor *F* with value of 0.190, which reduced the particle-size estimation error from 56% to 6.8% for particles with a diameter of 1.50 μm. With respect to smaller particles, the correction reduced the relative error from 57% to 2% for particles with *a* = 500 nm and from 61% to 10% for particles with *a* = 250 nm. Overall, this study contributes to increasing research knowledge on the use of stochastic electrochemistry for particle-size quantification. Ongoing work in our research group is focused on expanding the applicability of this correction estimation to other systems, for example, those related to bacterial cells.

## Supplementary Material



## Notes

Steady-state voltammetry for the different UMEs used in this study; details of the numerical simulations and further analysis for our analytical treatment applied for increasing the accuracy of the particle-size calculation; experiments under different supporting electrolyte concentrations as well as histograms, convergence plots, and statistical data collected in this work (PDF)
