# Supplementary material for: Quantitative Compensation of Edge Effects for Particle Sizing in Stochastic Electrochemical Collision Experiments
Source: ACS Meas Sci Au. 2026 Sep 11;6(5):1944–56. doi: 10.1021/acsmeasuresciau.6c00290 (PMC13693430; doi:10.1021/acsmeasuresciau.6c00290)
Supplement: Supplementary file 1 [file tg6c00290_si_001.pdf]

Supporting Information

Quantitative Compensation of Edge Effects for Particle Sizing in Stochastic Electrochemical Collision Experiments

Alfredo Eduardo Chávez-Ramos[2], Jake Saddler[1], Drake Katt[1], Mónica Galicia[2], César Alfonso Ortiz-Ledón*[1]

[1] Department of Chemistry and Biochemistry, Eastern Illinois University, 61920, United States

[2]Departamento de Ciencias Químico-Biológicas, Instituto de Ciencias Biomédicas, Universidad Autónoma de Ciudad Juárez, 32310, CH, México.

Corresponding Autor

*César A. Ortiz-Ledón: Telephone 217-581-2720; Email: caortiz2@eiu.edu

Numerical simulations: Finite-element simulations using COMSOL 5.4 were employed to model diffusion-limited current at a Pt UME. The concentration field of the redox species was described by the steady-state diffusion equation:

$$\nabla^2 C = 0 \qquad \text{Eq. S1}$$

An axisymmetric geometry was used to represent the disk electrode, with radii of (a) 29.4, b) 13.7, c) 5.8 µm and d) 1.6 µm. An insulating spherical particle of radius 1.5 µm was placed at defined positions relative to the electrode surface. More detail of this model can be found in the following sections where these are described thoroughly.

Boundary conditions were defined as follows: at the electrode surface, a diffusion-limited condition was imposed ($C^* = 0$), corresponding to rapid electron transfer kinetics. With respect to the insulating particle surface, a no-flux boundary condition was applied.

$$n \cdot \nabla C = 0 \qquad \text{Eq. S2}$$

Regarding the outer boundaries of the simulation domain, the concentration was fixed to the bulk value. The computational domain was chosen sufficiently large to avoid boundary-induced artifacts, and the mesh was refined near the electrode and particle surfaces to accurately resolve concentration gradients.[1,2]

Simulations were performed for a 2.5 mM ferrocenemethanol solution. The particle was positioned both at the center and near the edge of the electrode surface to capture spatial variations in the diffusion field. The faradaic current was calculated by integrating the diffusive flux over the electrode surface

$$i = nF \int J \cdot dA \qquad \text{Eq. S3}$$

and current steps were determined from the difference between particle-blocked and particle-free These simulated current steps were used to quantify edge effects and to derive an analytical correction for particle size estimation. To determine the flux blocked by a PS bead, we approximated the number of PS beads that can fit in a ring at the edge and center of the UME. To estimate the number of PS beads, the following equation was employed:

$$N = \frac{\pi D_{ume}}{D_{PS}} \qquad \text{Eq. S4}$$

Where N is the number of PS beads that can be set on the modeled ring, D_{ume} is the diameter of the electrode tested and D_{PS} *is 3 µm.* Figure S9 shows the geometry to simulate the current response in both presence and absence of the PS bead. Figure S10 and S11 depict the concentration profiles at +0.4 V *vs.* Ag/AgCl and illustrate the behavior of the flux been blocked by the PS bead.

Edge-flux correction factor (*F*) derivation. This factor was derived by observing our experimental and simulated data. First, we found that experimentally and supported by the numerical simulations that:

$$\Delta i_{center} < \Delta i_{exp} < \Delta i_{edge}$$

This establishes the current response has two limits, edge and center response, the experimental response can lie between these two limits. Therefore, *P* is defined in the main text and *P*= 0 the response behaves like the center and *P*=1 has edge-like response. For the derivation of F, these current steps and P are combined to relate how the measured current behaves as weighted average of the two limiting responses. Therefore, we can write the following expression:

$$\Delta i_{exp} = P\Delta i_{edge} + (1-P)\Delta i_{center}$$

By rearranging this equation and dividing by Δi_{edge} , we can find the following equation:

$$\frac{\Delta i_{exp}}{\Delta i_{edge}} = P + (1-P)\frac{\Delta i_{center}}{\Delta i_{edge}}$$

Ultimately, this can be further reduced to Equation 3 from the main text of this manuscript:

$$F = P + (1-P)\beta$$

Where, F and β are defined as:

$$\beta = \Delta i_{center}/\Delta i_{edge}$$

$$F = \Delta i_{exp}/\Delta i_{edge}$$

Because the experimentally measured blocking currents always fall between the simulated edge- and center-limited responses, we approximate the spatially averaged blocking current using a first-order linear interpolation between these two limiting cases. This reduced-order approximation replaces the continuous spatial integration of the flux distribution with a weighted average of its extrema while preserving the experimentally observed current bounds.

Different supporting electrolyte concentrations: Blocking collision experiments were performed using PS beads with *a* = 1.50 μm at a Pt UME with *a* = 5.80 μm while varying the KCl concentration and maintaining the ferrocenemethanol concentration at 2.5 mM to evaluate the effect of migration on the current step magnitude. As shown in Figure S4, collision experiments conducted with 10 fM PS beads at different KCl concentrations demonstrate that the number of collision events decreases markedly as the KCl concentration increases. This behavior is consistent with previous studies, which reported that, at low

supporting electrolyte concentrations, migration becomes the dominant mode of mass transport for polymeric particles.[3–5]

Our results agree with these findings. The number of collision events decreased from 22 collisions at 1 mM KCl to only 1 collision at 5 mM KCl and 2 collision at 25 mM KCl. In contrast, the current step magnitude remained statistically unaffected within experimental error. The average current step at 1 mM KCl was 61 ± 31 pA, while the two collision events observed at 25 mM KCl produced current steps of 71 and 57 pA, and the single event at 5 mM KCl produced a current step of 44 pA. These results suggest that, under our experimental conditions, the current step magnitude is not significantly affected by the supporting electrolyte concentration.

As the number of collision events resulted substantially lower at higher KCl concentrations, all subsequent experiments were performed in 1 mM KCl to obtain an enough number of events for assessment of edge effects on particle size prediction.

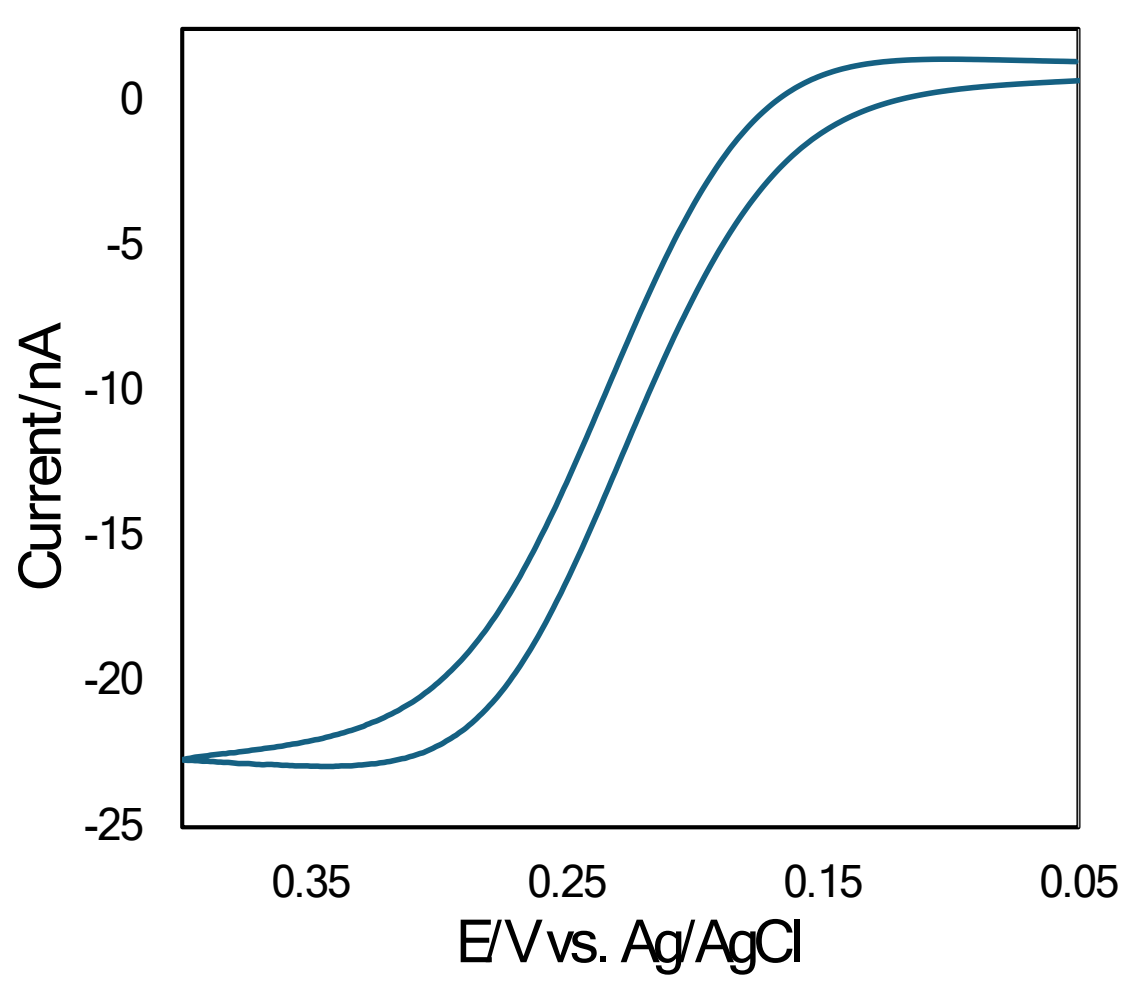

Figure S1. Steady-state voltammetry of a Pt UME with a=29.4 µm in 2.5 mM FcMeOH +1 mM KCl. Scan rate=0.02 Vs^{-1}.

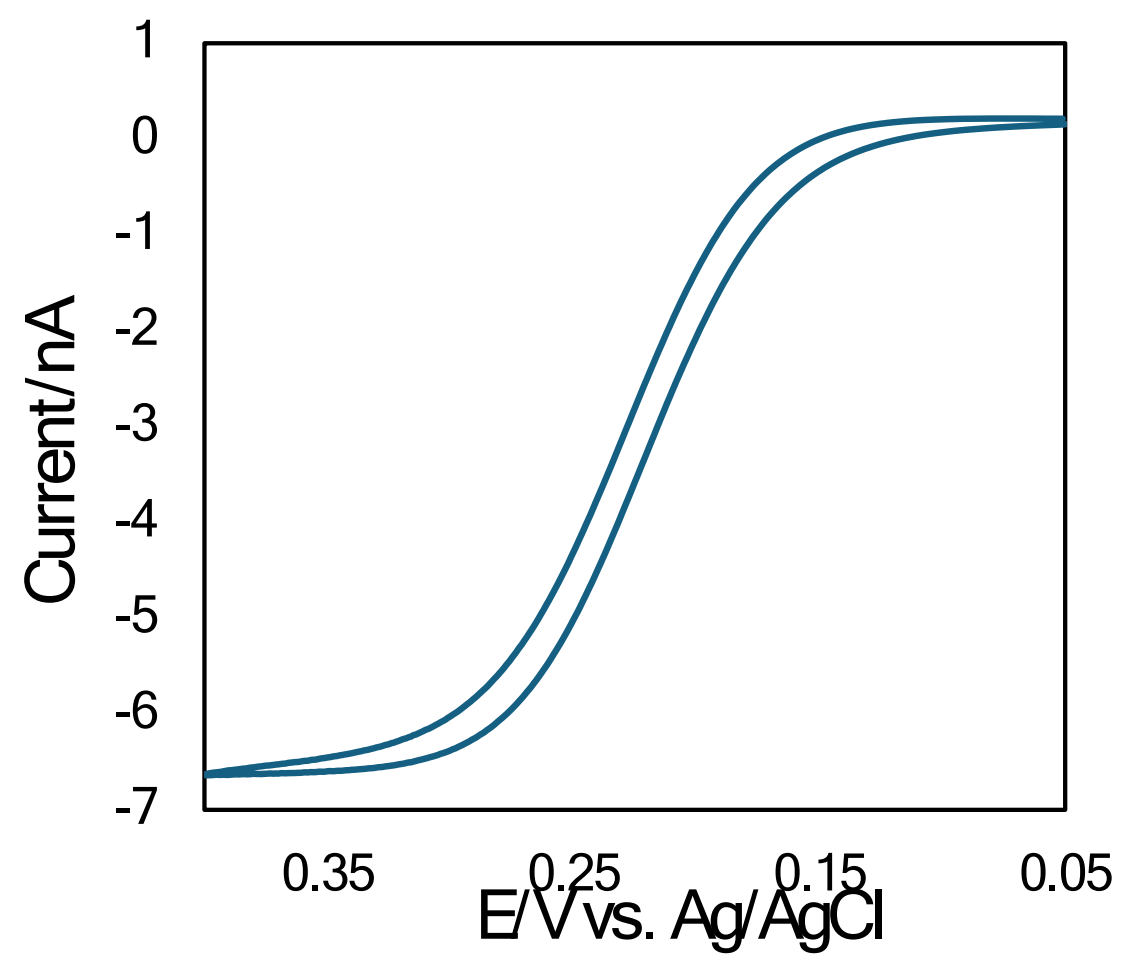

Figure S2. Steady-state voltammetry of a Pt UME with *a*=13.7 µm in 2.5 mM FcMeOH +1 mM KCl. Scan rate=0.02 Vs^{-1}.

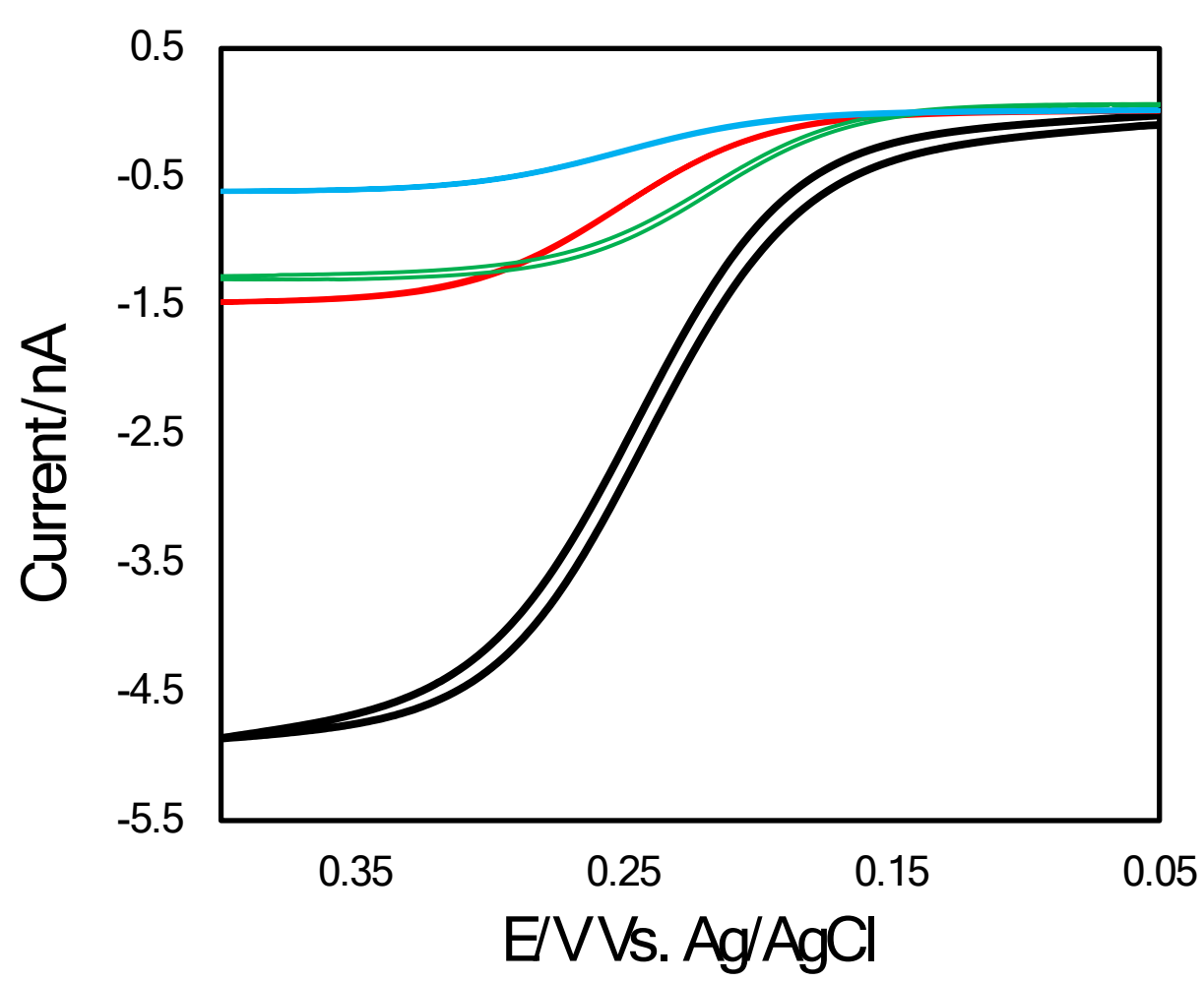

Figure S3. Steady state voltammograms of a 2.5 mM FcMeOH +1 mM KCl on three different Pt UMEs with *a*=5.80 µm (black), *a*=2.04 µm (red), *a*=1.6 µm and, *a*=0.80 µm (blue). Scan rate 0.02 V s^{-1}. These UMEs were used in this work to determine the particle size of PS beads with different sizes.

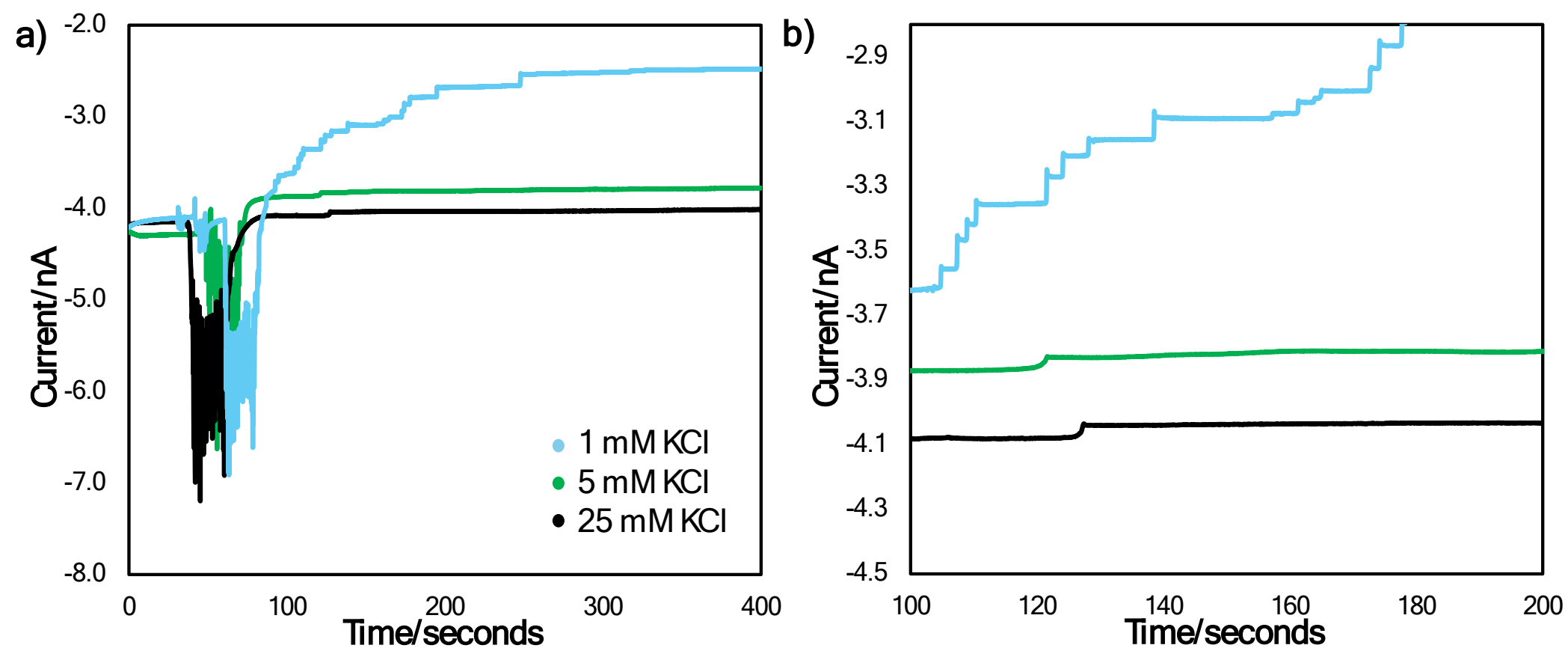

Figure S4. Collisions of PS bead with a=1.50 μm on a Pt UME with a=5.80 μm in different KCl concentration, PS bead concentration was 10 fM. The testing solution was 2.5 mM FcMeOH. Applied potential was +0.4 V vs. Ag/AgCl (KCl sat'd). Time interval 0.08 s.

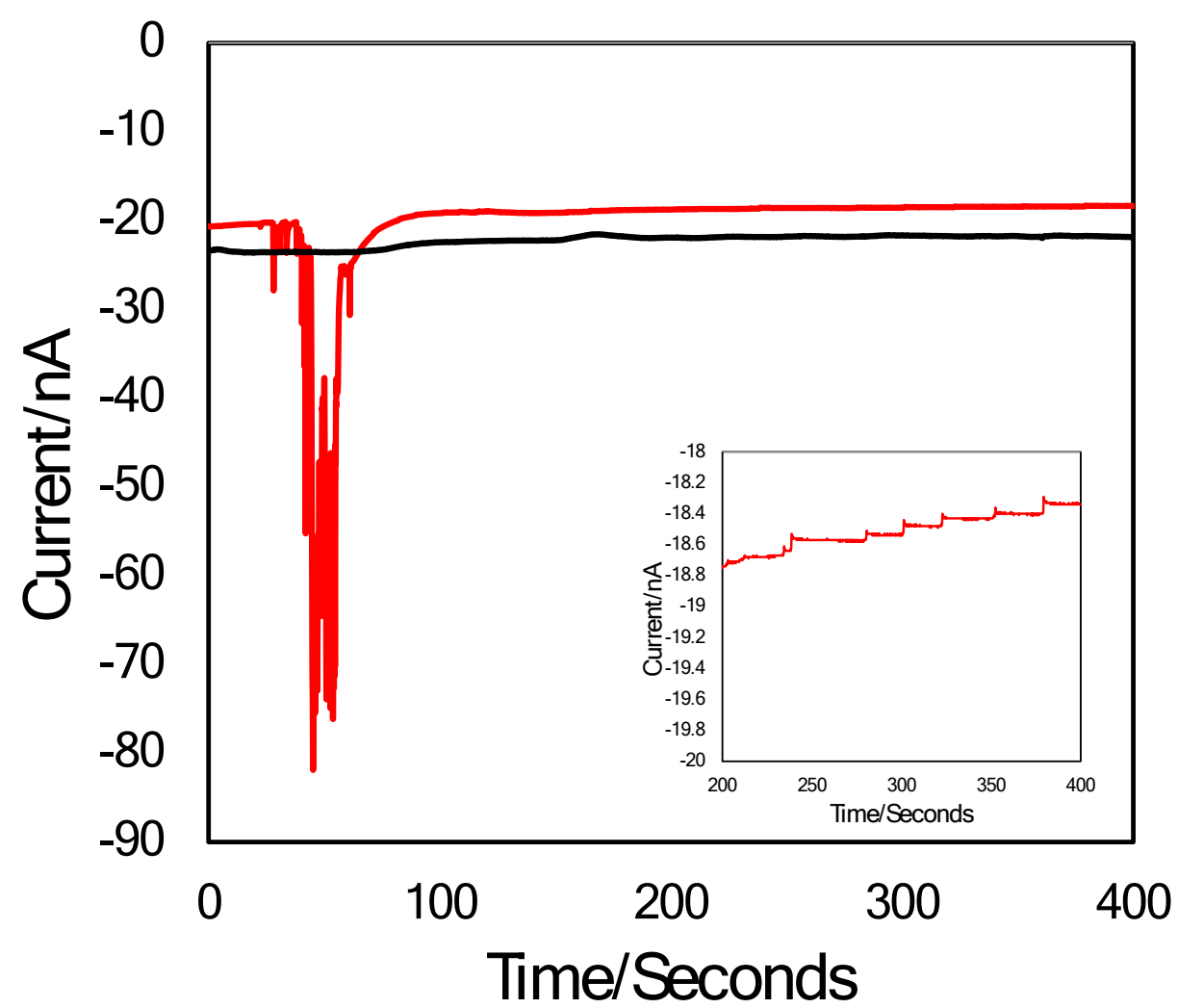

Figure S5. Collisions of PS bead with a=1.50 μm on a Pt UME with a=29.4 μm, PS bead concentration was 3 fM. The black trace represents the i-t curve recorded with no PS beads.

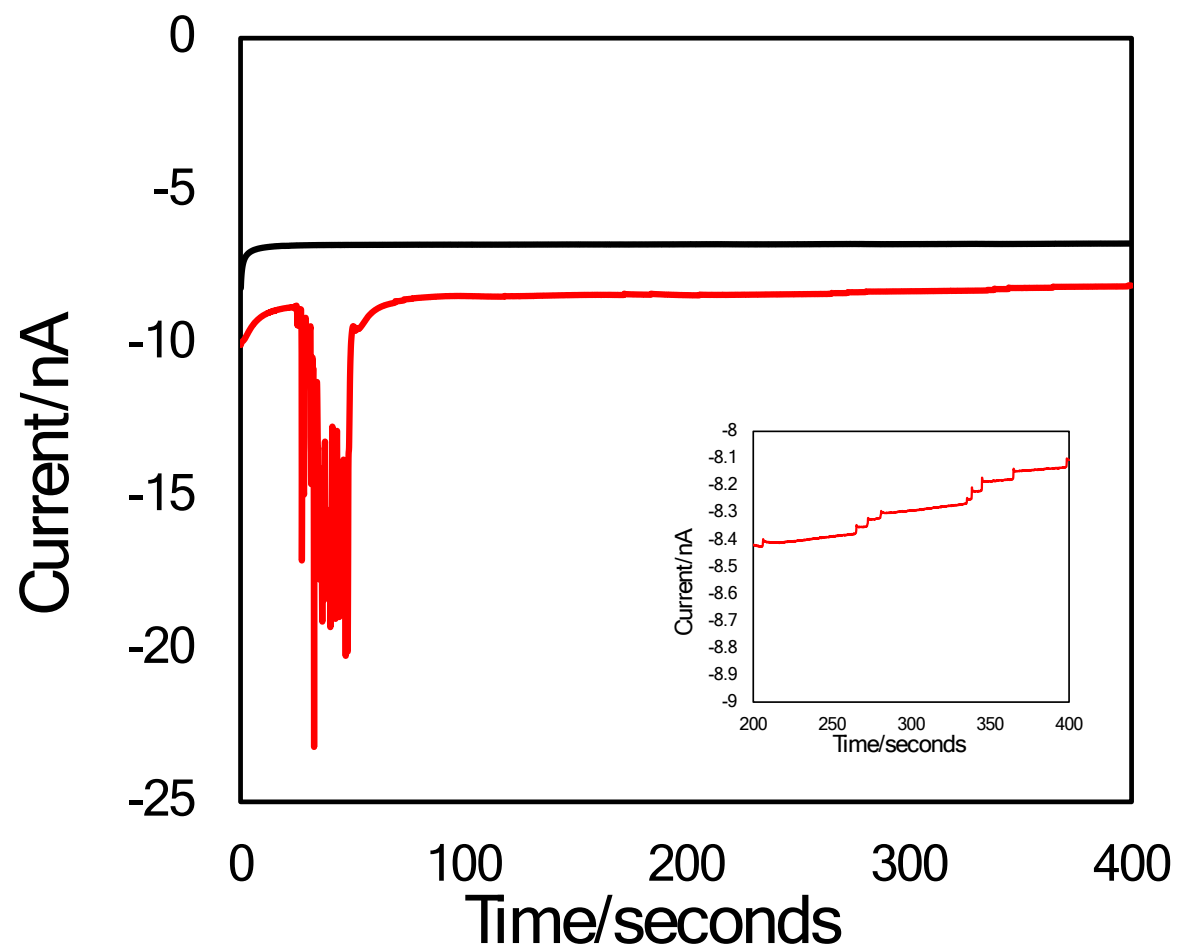

Figure S6. Collisions of PS bead with a=1.50 µm on a Pt UME with a=13.7 µm, PS bead concentration was 4 fM. The black trace represents the i-t curve recorded with no PS beads.

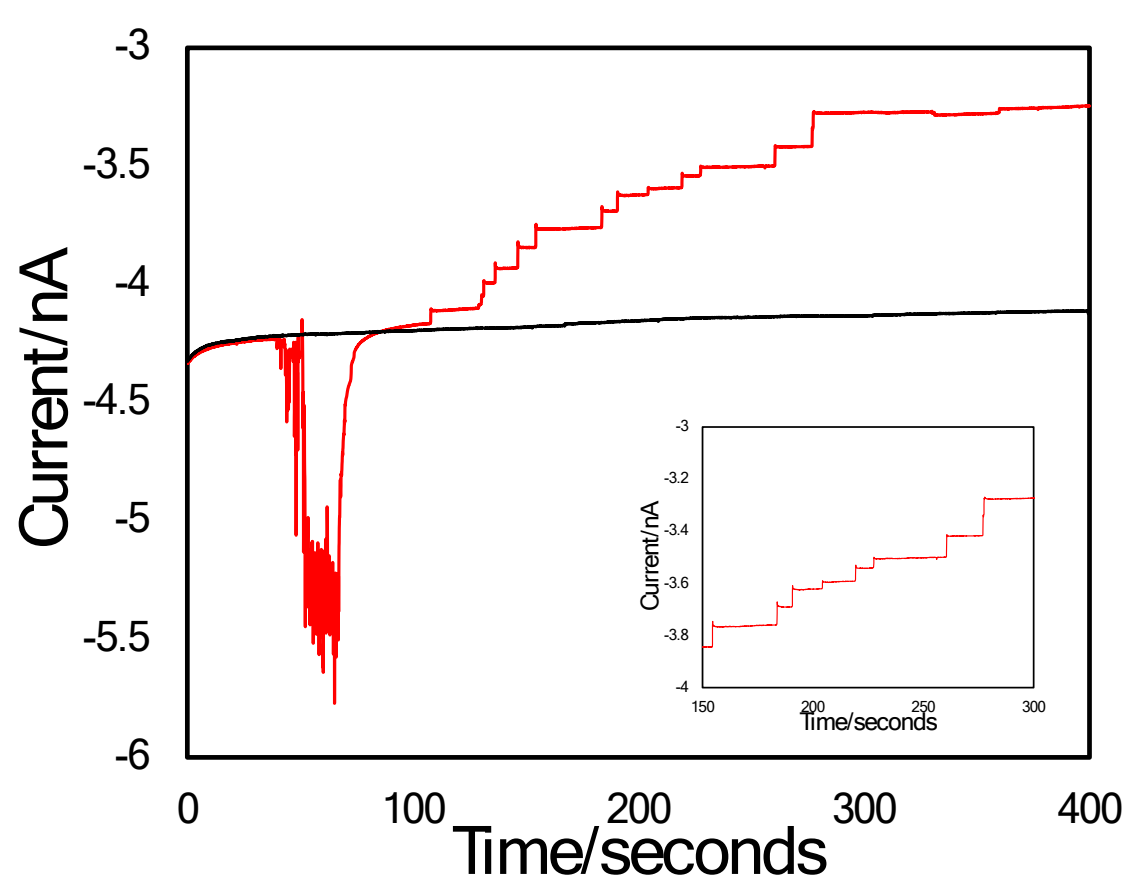

Figure S7. Collisions of PS bead with a=1.50 µm on a Pt UME with a=5.80 µm, PS bead concentration was 5 fM. The black trace represents the i-t curve recorded with no PS beads.

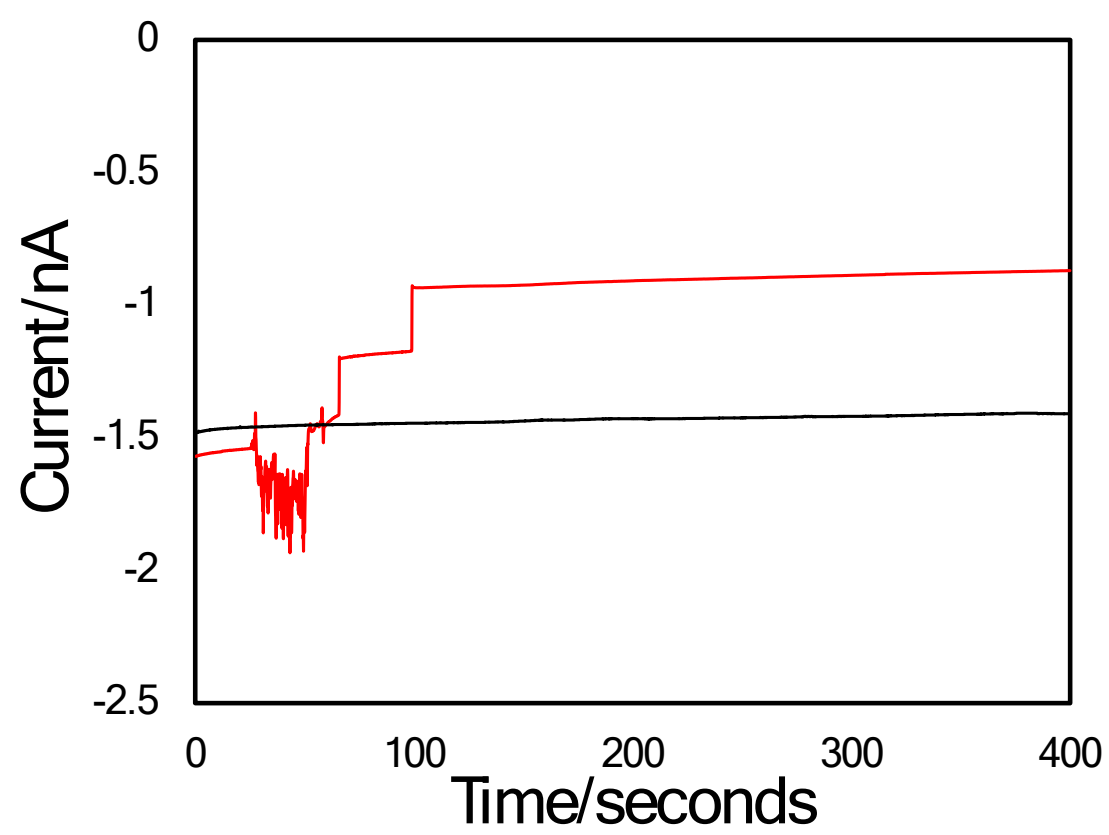

Figure S8. Collisions of PS bead with a=1.50 μm on a Pt UME with a=1.60 μm, PS bead concentration was 4 fM. The black trace represents the i-t curve recorded in absence of PS beads.

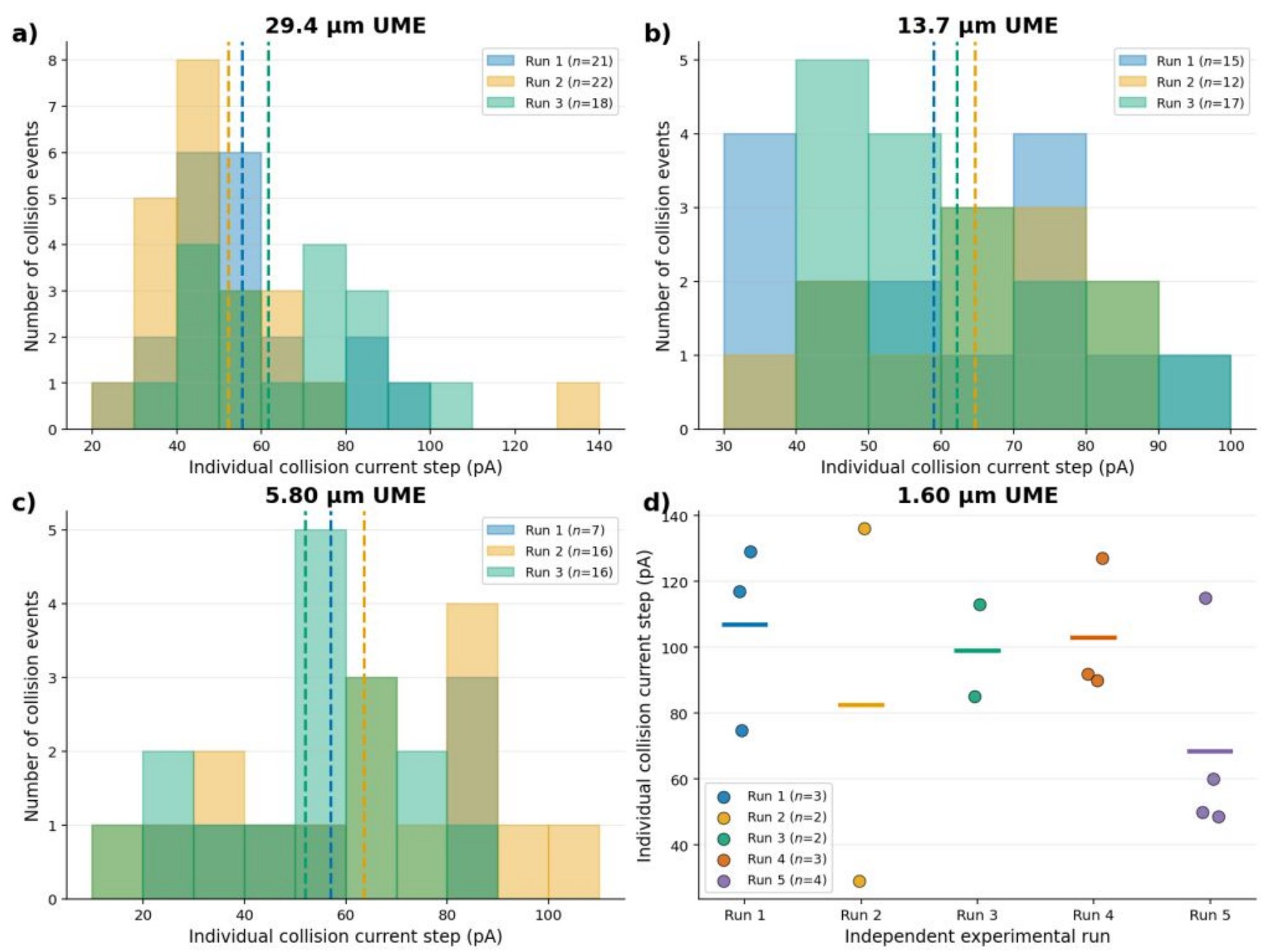

Figure S9. Distribution of individual collision currents measured using UMEs of different diameters. (a–c) Histograms showing the distribution of individual collision current steps recorded during three independent experimental runs using 29.4 μm (a), 13.7 μm (b), and 5.80 μm (c) diameter UMEs. Collision events are grouped by experimental run and shown as overlapping histograms: Run 1 (blue), Run 2 (yellow), and Run 3 (green). The number of collision events included in each run is indicated in the legend. Dashed vertical lines denote the mean individual collision current step for each corresponding experimental run. (d) Individual collision current steps measured using a 1.60 μm diameter UME across five independent experimental runs. Individual measurements are shown as points, with the number of events analyzed in each run indicated in the legend; horizontal lines represent the mean collision current step for each run. Here, n denotes the number of individual collision events.

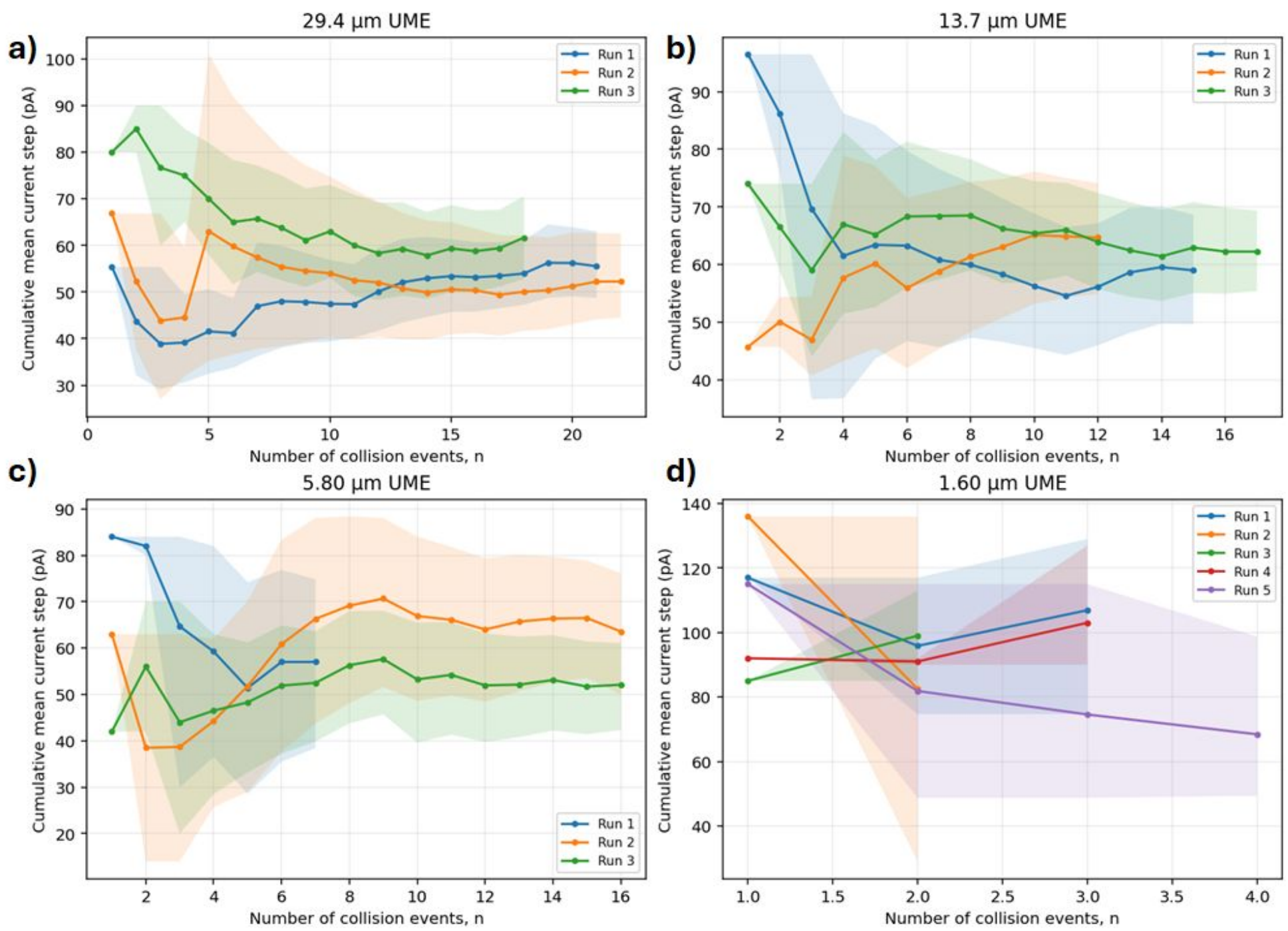

Figure S10. Convergence of the cumulative mean collision current with increasing number of collision events. (a–d) Cumulative mean collision current step as a function of the number of individual collision events (*n*) for 29.4 µm (a), 13.7 µm (b), 5.80 µm (c), and 1.60 µm (d) diameter UMEs. Each colored trace represents an independent experimental run. The cumulative mean at each value of *n* was calculated from the collision events accumulated sequentially within the corresponding experimental run. Shaded regions represent 95% bootstrap confidence intervals calculated from 10,000 resamples of the collision events included at each value of *n*. *N* denotes the number of independent experimental runs, whereas *n* denotes the number of individual collision events. The lower number of collision events observed for the 1.60 µm UME reflects the intrinsically lower collision probability under conditions where the electrode and particle dimensions are comparable.

Table S1. Run-level statistical data from collision experiments performed in this study using PS beads with a = 1.50 µm.

UME radius (µm)	Collision Frequency (s^{-1})	Mean current step (pA)	SD of run-means (pA)	SEM (pA)	95% CI (pA)	N	Total number of collisions
1.6	0.008 ± 0.002	91.97	16.11	7.21	71.96–111.97	5	14
5.80	0.031 ± 0.010	57.55	5.75	3.32	43.24–71.85	3	39
13.7	0.042 ± 0.007	61.97	2.85	1.64	54.87–69.06	3	44
29.4	0.057 ± 0.005	56.48	4.78	2.76	44.60–68.35	3	61

Table S2 Collision-level statistics for individual experimental runs for PS bead with *a*=1.5 μm on different UMEs.

UME radius (μm)	Run	n Collision events	Mean current step (pA)	SD of individual collisions (pA)
29.4	1	21	55.53	17.14
29.4	2	22	52.25	22.26
29.4	3	18	61.67	20.07
13.7	1	15	58.99	19.68
13.7	2	12	64.69	17.72
13.7	3	17	62.24	15.28
5.80	1	7	57.00	26.88
5.80	2	16	63.56	27.71
5.80	3	16	52.09	19.78
1.60	1	3	106.90	28.52
1.60	2	2	82.50	75.66
1.60	3	2	99.00	19.8
1.60	4	3	103.00	20.81
1.60	5	4	68.43	31.46

Table S3. Run-level particle size estimation for PS beads with *a*=1.5 μm using Eq. 1.

UME radius (μm)	N	Total collisions, n	Mean r_{PS} (μm)	SD(μm)	SEM (μm)	95% CI
1.6	5	14	0.431	0.038	0.017	0.384–0.478
5.8	3	39	0.667	0.034	0.02	0.581–0.753
13.7	3	44	1.073	0.025	0.015	1.009–1.137
29.4	3	61	1.526	0.065	0.038	1.365–1.688

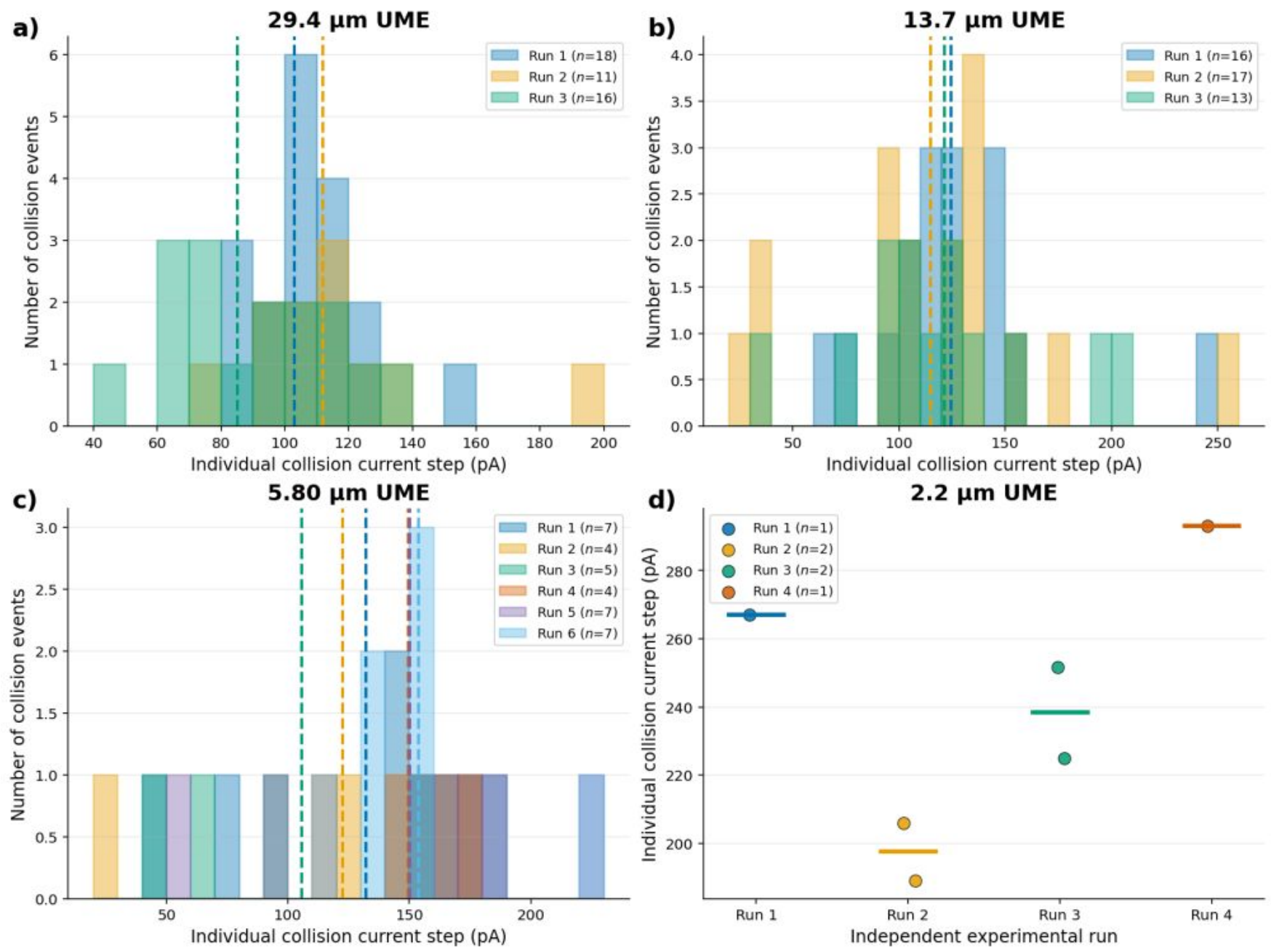

Figure S11. Individual collision current-step distributions for a=2.25 µm PS beads at different UMEs. Histograms show the current-step values obtained from individual nanoparticle collision events for (a) 29.4 µm, (b) 13.7 µm, and (c) 5.80 µm diameter UMEs. Histograms were constructed using 10 pA bins. Different colors represent independent experimental runs, and the dashed vertical lines indicate the mean current step for each run. The number of collision events (n) analyzed in each run is indicated in the legend. (d) Individual collision current-step values for the 2.20 µm UME are shown as individual data points because of the limited number of collision events obtained in each run. Horizontal lines indicate the mean current step for each run. Here, n denotes the number of individual collision events.

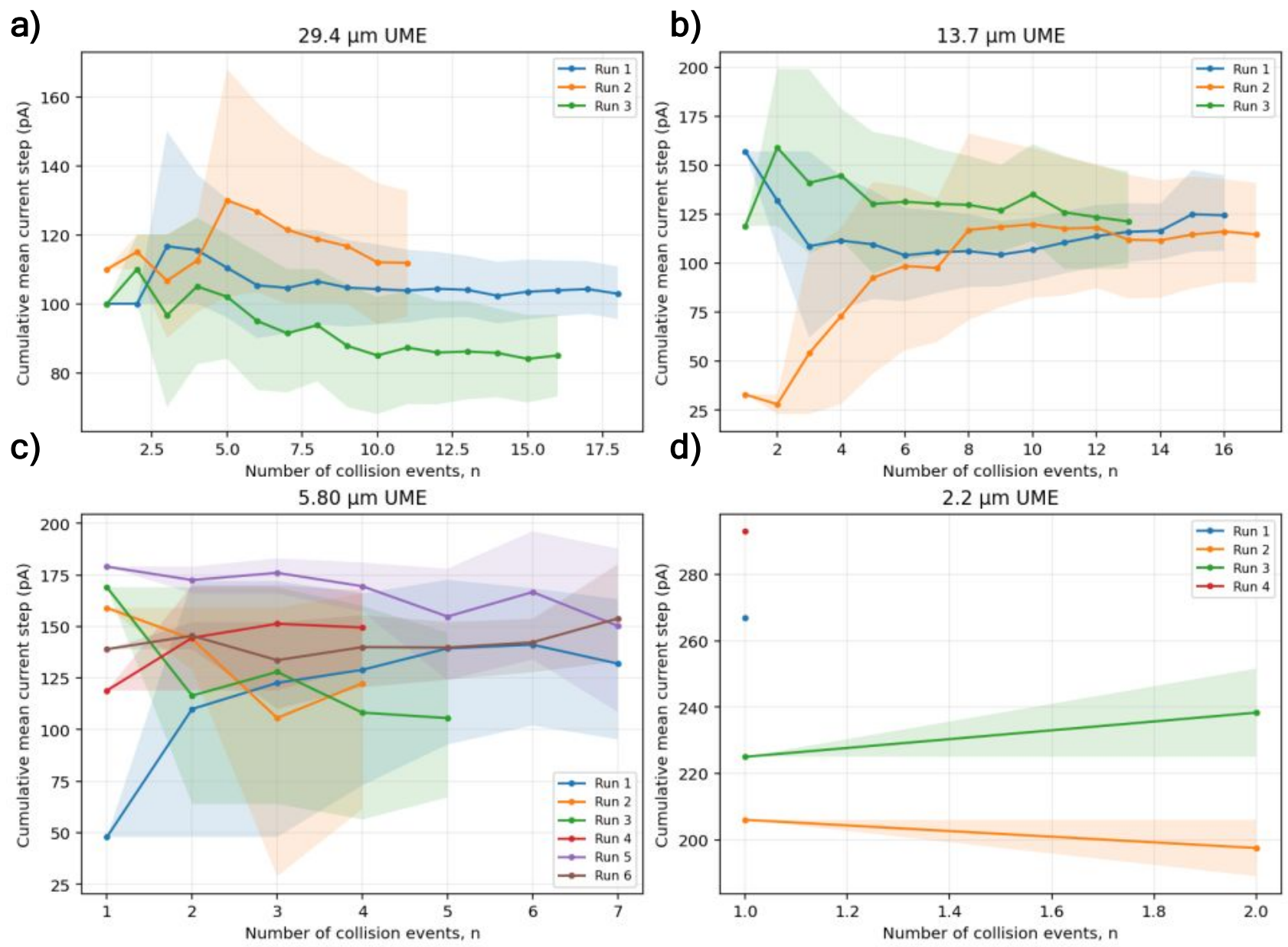

Figure S12. Convergence of the cumulative mean collision current for 2.25 µm-radius polystyrene particles. (a–d) Cumulative mean collision current step as a function of the number of individual collision events (*n*) for 29.4 µm (a), 13.7 µm (b), 5.80 µm (c), and 2.2 µm (d) UMEs. Each colored trace represents an independent experimental run. The cumulative mean at each value of *n* was calculated sequentially using the collision events accumulated within the corresponding experimental run. Shaded regions represent 95% percentile bootstrap confidence intervals calculated from 10,000 resamples of the collision events included at each value of *n*. *N* denotes the number of independent experimental runs, whereas *n* denotes the number of individual collision events. The analysis was used to assess the stability and uncertainty of the estimated mean collision current as a function of the number of collision events.

Table S4. Run-level statistical data from collision experiments performed in this study using PS beads with $a = 2.25$ µm.

UME radius (µm)	Collision Frequency (s^{-1})	Mean current step (pA)	SD between experiments (pA)	SEM (pA)	95% CI (pA)	N	Total number of collisions
2.2	0.004 ± 0.002	248.95	40.93	20.47	183.82–314.08	4	6
5.80	0.015 ± 0.004	135.62	19.11	7.8	115.56–155.68	6	34
13.7	0.044 ± 0.006	120.12	4.96	2.86	111.01–129.24	3	46
29.4	0.043 ± 0.010	99.9	13.66	7.88	74.81–125.00	3	45

Table S5. Collision-level statistics for individual experimental runs for PS bead with a=2.25 µm on different UMEs.

UME radius (µm)	Run	n Collision events	Mean current step (pA)	SD of individual collisions (pA)
29.4	1	18	102.88	17.13
29.4	2	11	111.82	33.41
29.4	3	16	85.00	25.29
13.7	1	16	124.43	41.03
13.7	2	17	114.70	54.77
13.7	3	13	121.23	47.58
5.8	1	7	132.00	49.86
5.8	2	4	122.50	64.98
5.8	3	5	105.60	52.74
5.8	4	4	149.50	23.24
5.8	5	7	150.28	58.42
5.8	6	7	153.86	34.58
2.2	1	1	267	-
2.2	2	2	197.50	12.02
2.2	3	2	238.30	18.81
2.2	4	1	293	-

Table S6. Run-level particle size estimation for PS beads with a=2.25 µm using Eq. 1.

UME radius (µm)	N	Total collisions, n	Mean r_{PS} (µm)	SD(µm)	SEM (µm)	95% CI
2.2	4	6	0.879	0.074	0.037	0.762–0.997
5.8	6	34	1.023	0.074	0.03	0.946–1.100
13.7	3	46	1.487	0.03	0.017	1.412–1.562
29.4	3	45	2.031	0.141	0.081	1.681–2.381

Table S7. Run-level particle size estimation for PS beads with a=1.5 µm using Eq. 4, with F values from Table 2.

UME radius (µm)	N	Total collisions, n	Mean r_{PS} (µm)	SD(µm)	SEM (µm)	95% CI
1.6	5	14	0.914	0.083	0.037	0.811–1.017
5.8	3	39	1.531	0.076	0.044	1.342–1.720
13.7	3	44	1.574	0.037	0.021	1.483–1.665
29.4	3	61	1.568	0.066	0.038	1.404–1.732

Table S8. Run-level particle size estimation with Pt UME with a=5.9 µm using PS beads with a= 1.5 µm

	Mean rp (µm)	SD (µm)	SEM (µm)	95% CI (µm)
Eq. 1	0.687	0.036	0.021	0.598–0.777
Eq. 2 (F=0.19)	1.576	0.083	0.048	1.369–1.783

Table S9. Particle size estimation with Pt UME with a=2.04 µm using PS beads with a= 500 nm. The run-level statistical analysis was estimated from two collision experiments (N=2).

	Mean r_{PS} (nm)	SD (nm)	SEM (nm)	95% CI (nm)
Eq. 1	213	4	3	177-249
Eq. 2 (F=0.19)	490	9	6	416-454

Table S10. Particle size estimation with Pt UME with a=800 nm using PS beads with a= 250 nm. The run-level statistical analysis was estimated from two collision experiments (N=2).

	Mean r_{PS} (nm)	SD (nm)	SEM (nm)	95% CI (nm)
Eq. 1	97	2	2	75.4-118.
Eq. 2 (F=0.19)	223	4	3	172.7-271.8

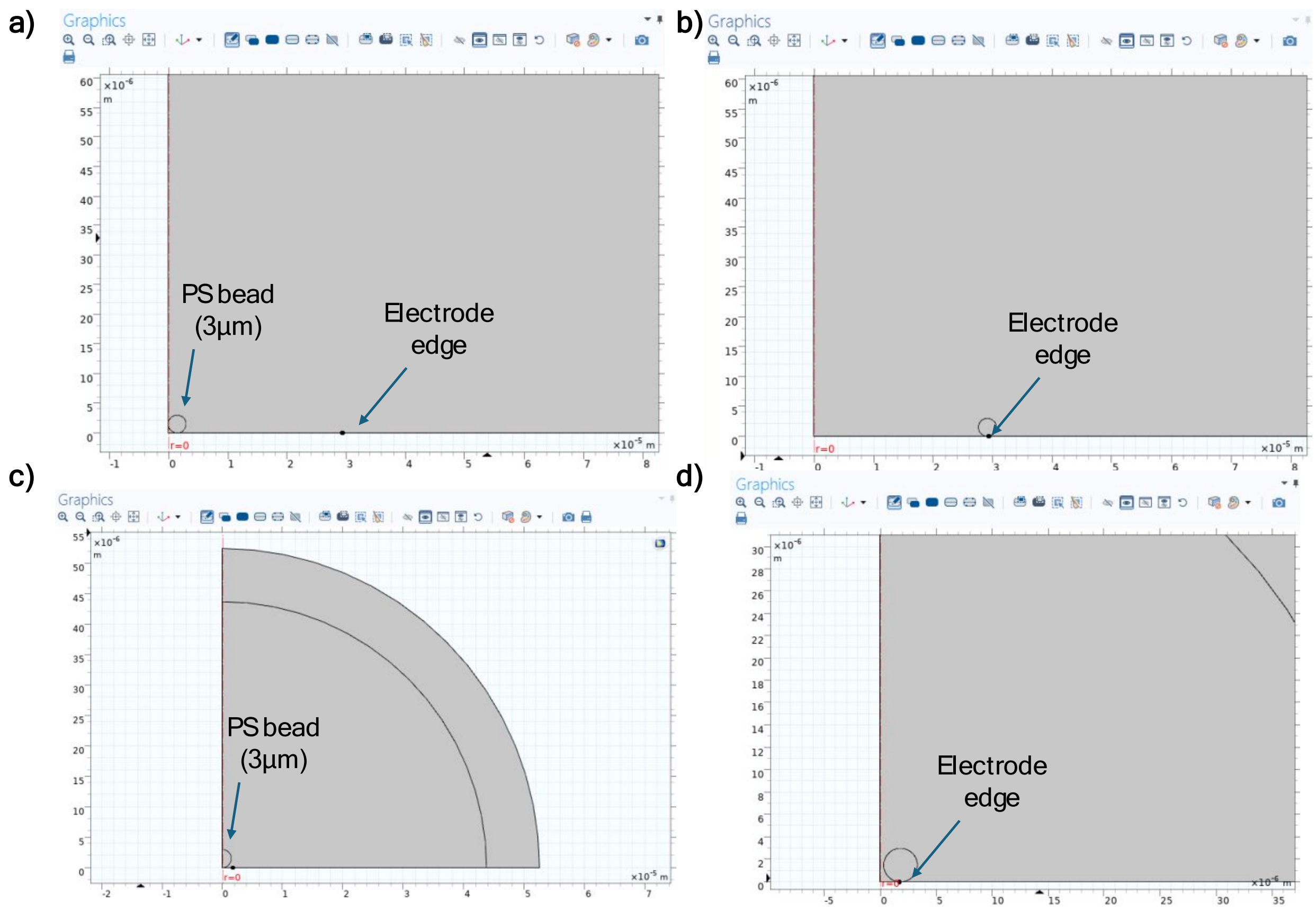

Figure S13. The constructed geometry was established for computing the simulations for the PS bead on a UME. The PS bead was positioned at edge and center of the electrode. The geometries were built to assess the different electrode sizes where a) and b) illustrate the geometries used for electrodes with a>5 µm and c) and d) when the $r_{ume}/r_{PS} \approx 1$.

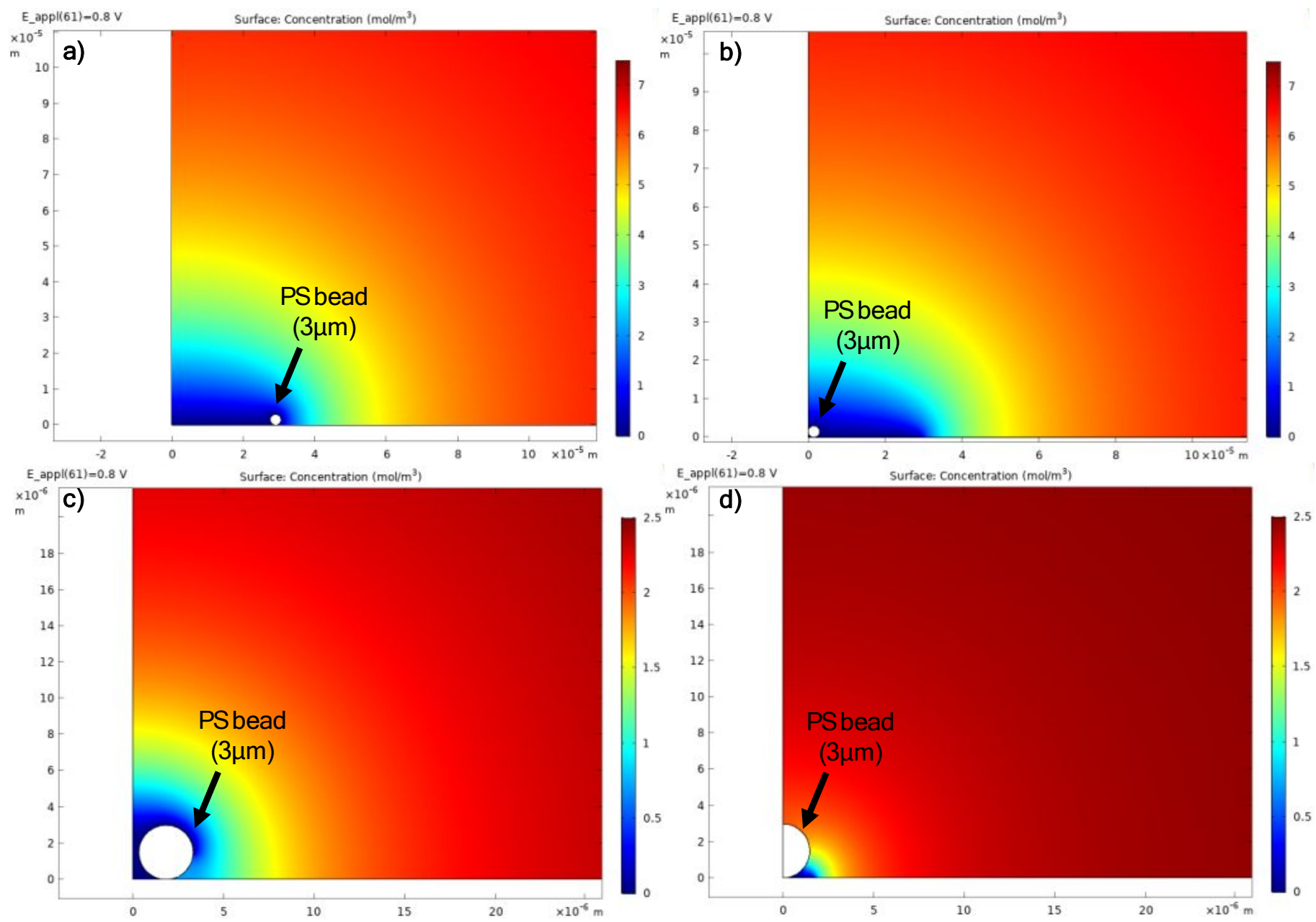

Figure S14. Concentration profiles with a PS bead adsorbed on the UME surface that exhibit the flux of ferrocenemethanol towards the electrode surface at steady state. The concentration maps shown in a) and b) reflect the profiles on UMEs with *a*> PS beads, whereas c) and d) maps illustrate when $r_{ume}/r_{PS} \approx 1$.

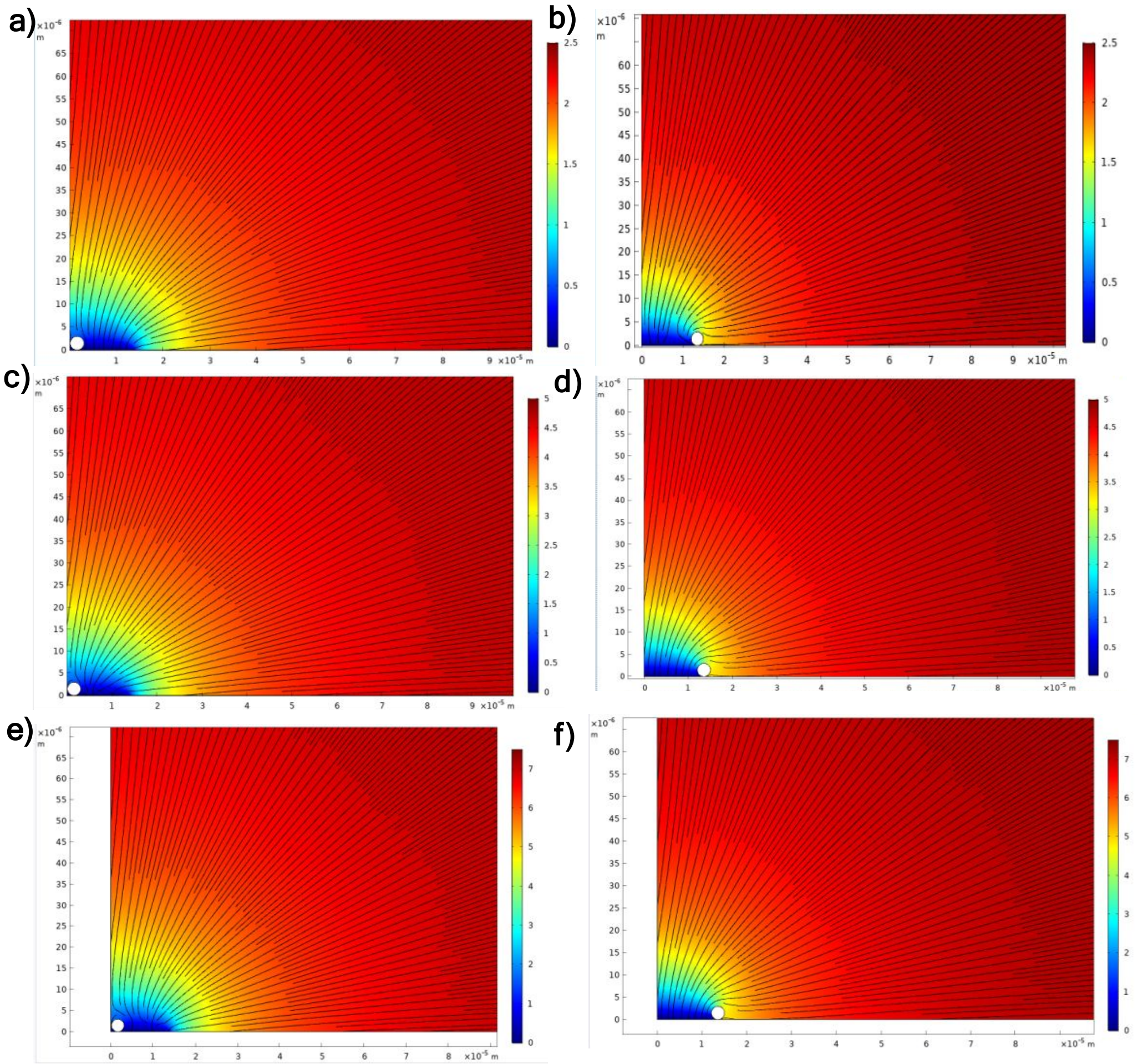

Figure S15. Steady-state concentration profiles for a polystyrene (PS) bead (a = 1.5 µm) adsorbed on the surface of an ultramicroelectrode (UME; a = 13.7 µm). The profiles illustrate the ferrocenemethanol concentration distribution and the corresponding diffusive flux toward the electrode surface at steady state for different bulk ferrocenemethanol concentrations. Panels a) and b) correspond to 2.5 mM, panels c) and d) to 5.0 mM, and panels e) and f) to 7.5 mM ferrocenemethanol. For each concentration, the left panel shows the bead located at the electrode center, while the right panel shows the bead positioned at the electrode edge. The color scale represents the ferrocenemethanol concentration, and the X and Y axes indicate distances within the simulated geometry.

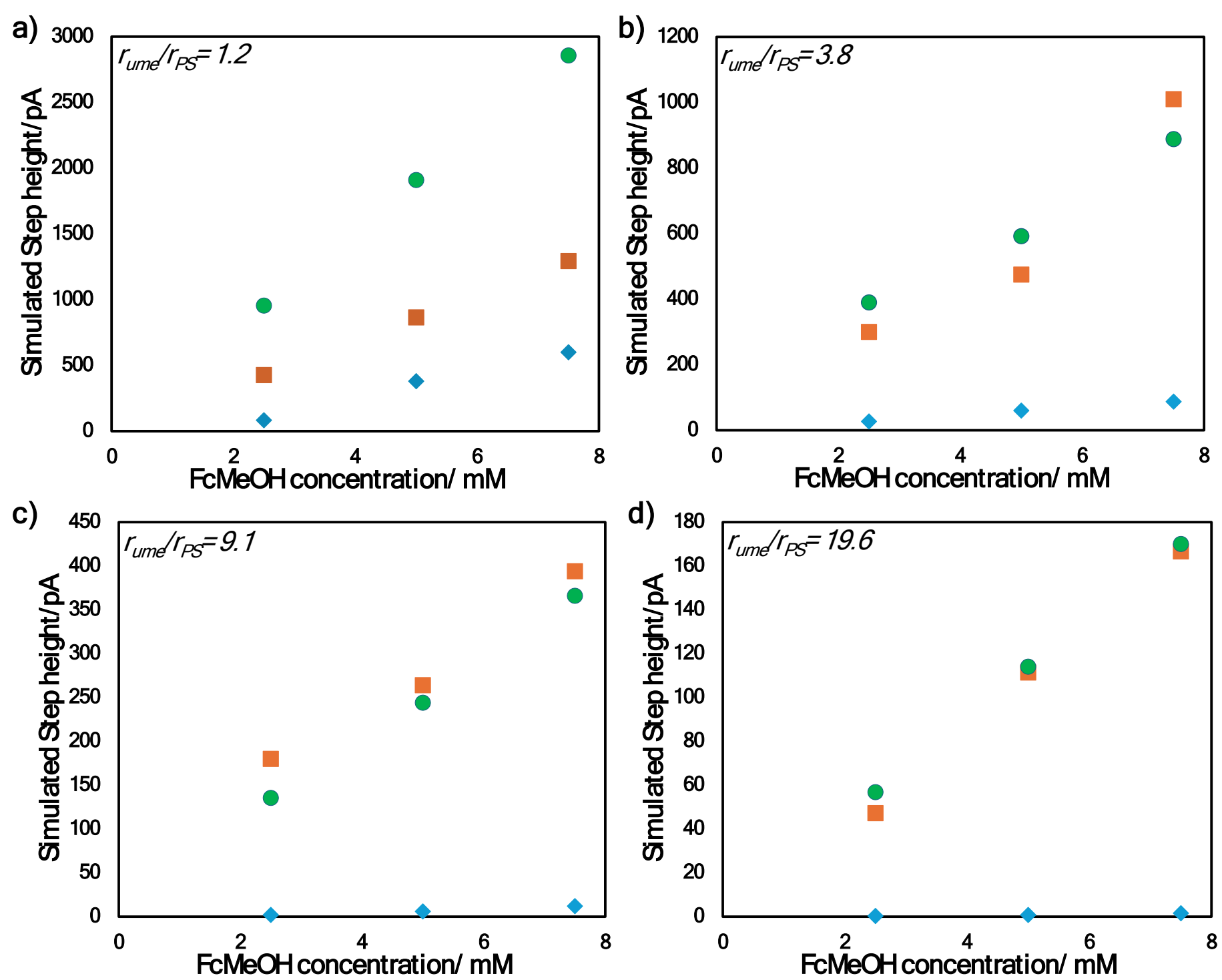

Figure S16. Simulated and theoretical current step at different redox mediator concentration with different UME sizes. a) *a*=1.6 µm, b) *a*=5.80 µm, c) *a*=13.70 µm, and d) *a*=29.40 µm. ● Theoretical, ■ Edge and ◆Center.

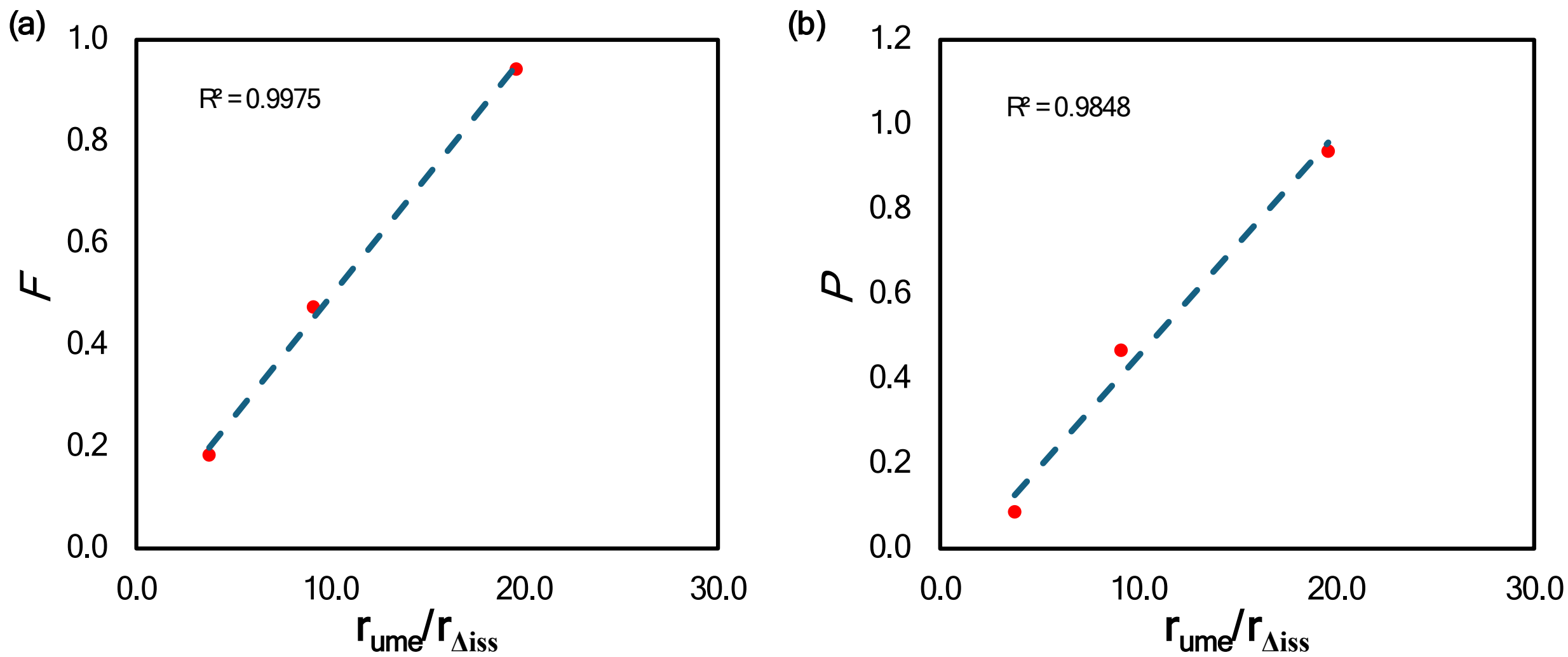

Figure S17. The values of F(a) and P(b) used to correct the particle size in this work were plotted against the ratio of $r_{UME}/r_{\Delta iss}$, to determine if this scales linearly.

References

(1) Boika, A.; Thorgaard, S. N.; Bard, A. J. Monitoring the Electrophoretic Migration and Adsorption of Single Insulating Nanoparticles at Ultramicroelectrodes. *Journal of Physical Chemistry B* 2013, *117* (16), 4371–4380. https://doi.org/10.1021/jp306934g.

(2) Park, J. H.; Boika, A.; Park, H. S.; Lee, H. C.; Bard, A. J. Single Collision Events of Conductive Nanoparticles Driven by Migration. *Journal of Physical Chemistry C* 2013, *117* (13), 6651–6657. https://doi.org/10.1021/jp3126494.

(3) Lemay, S. G.; Renault, C.; Dick, J. E. Particle Mass Transport in Impact Electrochemistry. *Current Opinion in Electrochemistry*. Elsevier B.V. June 1, 2023.

COMSOL report

Contents

1. Global Definitions 22
1.1. Parameters 22
2. Component 1 23
2.1. Definitions 23
2.2. Geometry 1 24
2.3. Electroanalysis 25
2.4. Mesh 1 43
3. Study 1 46
3.1. Parametric Sweep 46
3.2. Stationary 46
3.3. Solver Configurations 46
4. Results 55
4.1. Data Sets 55
4.2. Tables 56
4.3. Plot Groups 57

1 Global Definitions

Date	Jul 30, 2026 4:30:04 PM

USED PRODUCTS

COMSOL Multiphysics
Batteries & Fuel Cells Module

1.1 PARAMETERS

PARAMETERS 1

Name	Expression	Value	Description
E_appl	0[V]	0 V	Applied potential
Ef	0[V]	0 V	Formal potential
c_bulk	2.5[mmol/L]	2.5 mol/m³	Reactant concentration
re	13.7[um]	1.37E−5 m	Electrode radius
r_max	25*re	3.425E−4 m	Size of simulation space
D1	7.5e-10[m^2/s]	7.5E−10 m²/s	Reactant diffusion coefficient
D2	7.5e-10[m^2/s]	7.5E−10 m²/s	Product diffusion coefficient
k0	0.01[m/s]	0.01 m/s	Reaction rate
E_start	-0.4[V]	−0.4 V	Start potential
E_vertex	0.8[V]	0.8 V	Vertex potential
E_step	20[mV]	0.02 V	Potential step (output)

2 Component 1

Date	Jan 23, 2018 9:43:53 AM

SETTINGS

Description	Value
Unit system	Same as global system
Geometry shape order	Automatic

SPATIAL FRAME COORDINATES

First	Second	Third
r	phi	z

MATERIAL FRAME COORDINATES

First	Second	Third
R	PHI	Z

GEOMETRY FRAME COORDINATES

First	Second	Third
Rg	PHIg	Zg

MESH FRAME COORDINATES

First	Second	Third
Rm	PHIm	Zm

2.1 DEFINITIONS

2.1.1 Coordinate Systems

Boundary System 1

Coordinate system type	Boundary system
Tag	sys1

COORDINATE NAMES

First	Second	Third
t1	to	n

2.2 GEOMETRY 1

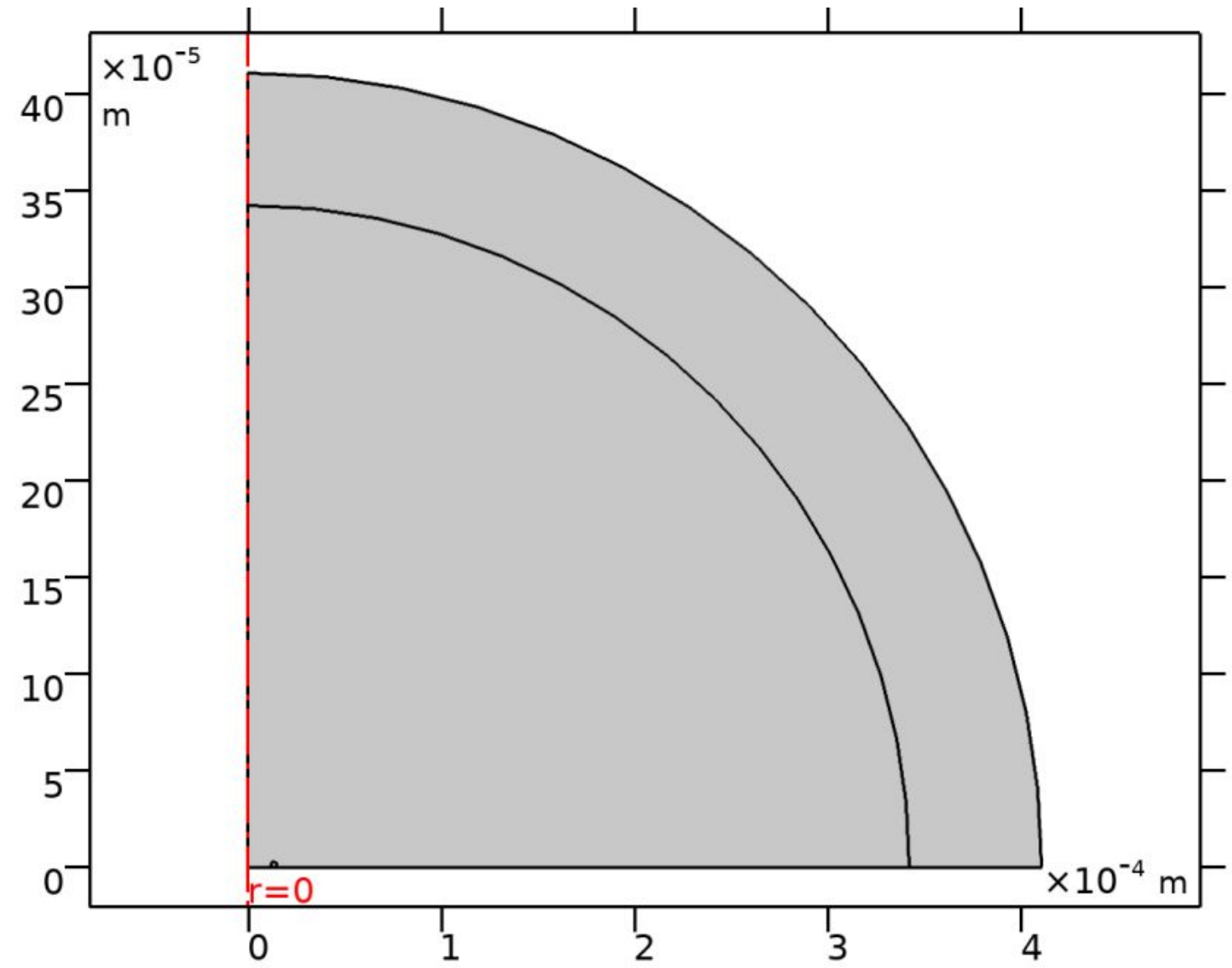

Geometry 1

UNITS

Length unit	m
Angular unit	deg

GEOMETRY STATISTICS

Description	Value
Space dimension	2
Number of domains	3
Number of boundaries	12
Number of vertices	10

2.2.1 Circle 1 (c1)

POSITION

Description	Value
Position	{0, 0}

SIZE AND SHAPE

Description	Value
Radius	r_max
Sector angle	90

2.2.2 Circle 2 (c2)

POSITION

Description	Value
Position	{0, 0}

SIZE AND SHAPE

Description	Value
Radius	1.2*r_max
Sector angle	90

2.2.3 Point 1 (pt1)

POINT

Description	Value
Point coordinate	{re, 0}

2.2.4 Circle 3 (c3)

POSITION

Description	Value
Position	{0.88*re, 0}
Base	Corner

SIZE AND SHAPE

Description	Value
Radius	1.5E-6

2.3 ELECTROANALYSIS

USED PRODUCTS

COMSOL Multiphysics
Batteries & Fuel Cells Module

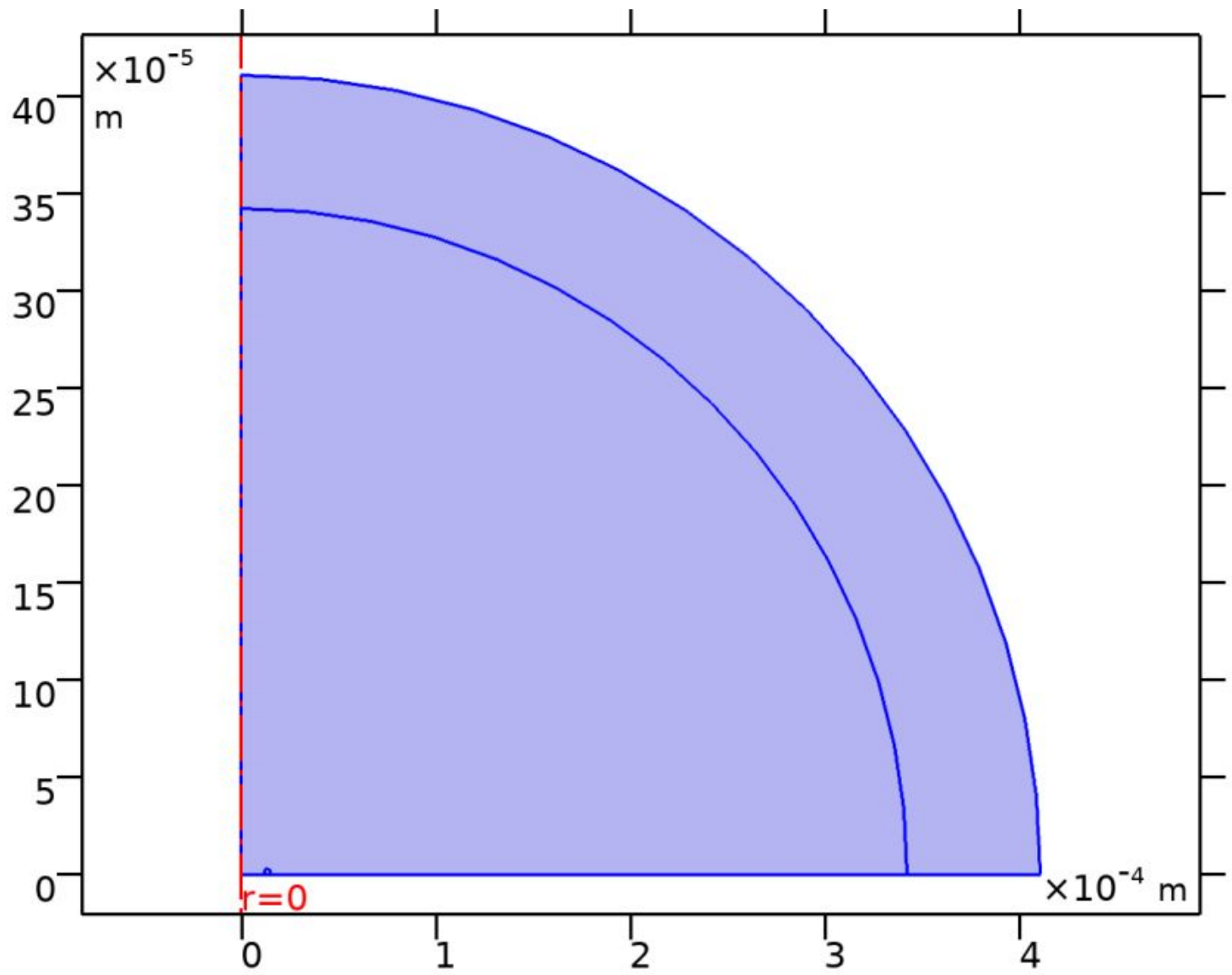

Electroanalysis

SELECTION

Geometric entity level	Domain
Selection	Domains 1–2

EQUATIONS

$$\nabla \cdot \mathbf{J}_i = R_i$$
$$\mathbf{J}_i = -D_i \nabla c_i$$
$$\phi_l = 0$$

2.3.1 Interface settings

Discretization

SETTINGS

Description	Value
Concentration	Linear

Transport mechanisms

SETTINGS

Description	Value
Convection	Off
Migration in electric field	Off

2.3.2 Variables

Name	Expression	Unit	Description	Selection	Details
domflux.Redr	2*elan.dflux_Redr*pi*r	mol/(m·s)	Domain flux, r component	Domains 1–2	

Name	Expression	Unit	Description	Selection	Details
domflux.Redz	2*elan.dflux_Redz*pi*r	mol/(m·s)	Domain flux, z component	Domains 1–2	
domflux.Oxir	2*elan.dflux_Oxir*pi*r	mol/(m·s)	Domain flux, r component	Domains 1–2	
domflux.Oxiz	2*elan.dflux_Oxiz*pi*r	mol/(m·s)	Domain flux, z component	Domains 1–2	
elan.mulstopcond	1	1	Multiplicative stop condition	Global	* operation
elan.stopcond	elan.mulstopcond	1	Solver stop condition	Global	
elan.nr	nr	1	Normal vector, r component	Boundary 7	
elan.nphi	0	1	Normal vector, phi component	Boundary 7	
elan.nz	nz	1	Normal vector, z component	Boundary 7	
elan.nr	unr	1	Normal vector, r component	Boundaries 9–12	
elan.nphi	0	1	Normal vector, phi component	Boundaries 9–12	
elan.nz	unz	1	Normal vector, z component	Boundaries 9–12	
elan.nr	dnr	1	Normal vector, r component	Boundaries 1–6, 8	
elan.nphi	0	1	Normal vector, phi component	Boundaries 1–6, 8	
elan.nz	dnz	1	Normal vector, z component	Boundaries 1–6, 8	
elan.nrmesh	root.nrmesh	1	Normal vector (mesh), r component	Boundary 7	
elan.nphimesh	0	1	Normal vector (mesh), phi component	Boundary 7	

Name	Expression	Unit	Description	Selection	Details
elan.nzmesh	root.nzmesh	1	Normal vector (mesh), z component	Boundary 7	
elan.nrmesh	unrmesh	1	Normal vector (mesh), r component	Boundaries 9–12	
elan.nphimesh	0	1	Normal vector (mesh), phi component	Boundaries 9–12	
elan.nzmesh	unzmesh	1	Normal vector (mesh), z component	Boundaries 9–12	
elan.nrmesh	dnrmesh	1	Normal vector (mesh), r component	Boundaries 1–6, 8	
elan.nphimesh	0	1	Normal vector (mesh), phi component	Boundaries 1–6, 8	
elan.nzmesh	dnzmesh	1	Normal vector (mesh), z component	Boundaries 1–6, 8	
elan.nrc	-root.nrc/elan.ncLen	1	Normal vector, r component	Boundaries 9–12	
elan.nphic	0	1	Normal vector, phi component	Boundaries 9–12	
elan.nzc	-root.nzc/elan.ncLen	1	Normal vector, z component	Boundaries 9–12	
elan.nrc	root.nrc/elan.ncLen	1	Normal vector, r component	Boundaries 1–8	
elan.nphic	0	1	Normal vector, phi component	Boundaries 1–8	
elan.nzc	root.nzc/elan.ncLen	1	Normal vector, z component	Boundaries 1–8	
elan.ncLen	sqrt(root.nrc^2+root.nzc^2+eps)	1	Help variable	Boundaries 1–12	
elan.ndflux_Red	elan.bndFlux_Red	mol/(m²·s)	Normal diffusive flux	Boundaries 2, 4–12	
elan.ntflux_Red	elan.bndFlux_Red	mol/(m²·s)	Normal total flux	Boundaries 2, 4–12	

Name	Expression	Unit	Description	Selection	Details
elan.ndflux_Oxi	elan.bndFlux_Oxi	mol/(m²·s)	Normal diffusive flux	Boundaries 2, 4–12	
elan.ntflux_Oxi	elan.bndFlux_Oxi	mol/(m²·s)	Normal total flux	Boundaries 2, 4–12	
elan.bndFlux_Red	if(r>0.001/sqrt(sqrt(mean(emetric2))),-0.5*dflux_spatial(Red)/(pi*r),NaN)	mol/(m²·s)	Boundary flux	Boundaries 1–6, 8	
elan.bndFlux_Red	if(r>0.001/sqrt(sqrt(mean(emetric2))),-0.5*uflux_spatial(Red)/(pi*r),NaN)	mol/(m²·s)	Boundary flux	Boundaries 9–12	
elan.bndFlux_Red	if(r>0.001/sqrt(sqrt(mean(emetric2))),0.25*(uflux_spatial(Red)-dflux_spatial(Red))/(pi*r),NaN)	mol/(m²·s)	Boundary flux	Boundary 7	
elan.bndFlux_Oxi	if(r>0.001/sqrt(sqrt(mean(emetric2))),-0.5*dflux_spatial(Oxi)/(pi*r),NaN)	mol/(m²·s)	Boundary flux	Boundaries 1–6, 8	
elan.bndFlux_Oxi	if(r>0.001/sqrt(sqrt(mean(emetric2))),-0.5*uflux_spatial(Oxi)/(pi*r),NaN)	mol/(m²·s)	Boundary flux	Boundaries 9–12	
elan.bndFlux_Oxi	if(r>0.001/sqrt(sqrt(mean(emetric2))),0.25*(uflux_spatial(Oxi)-dflux_spatial(Oxi))/(pi*r),NaN)	mol/(m²·s)	Boundary flux	Boundary 7	
elan.R_Red	0	mol/(m³·s)	Total rate expression	Domains 1–2	+ operation
elan.R_Oxi	0	mol/(m³·s)	Total rate expression	Domains 1–2	+ operation

Name	Expression	Unit	Description	Selection	Details
elan.nil	0	A/m²	Inward electrolyte current density	Domains 1–2	+ operatio n

2.3.3 Transport Properties 1

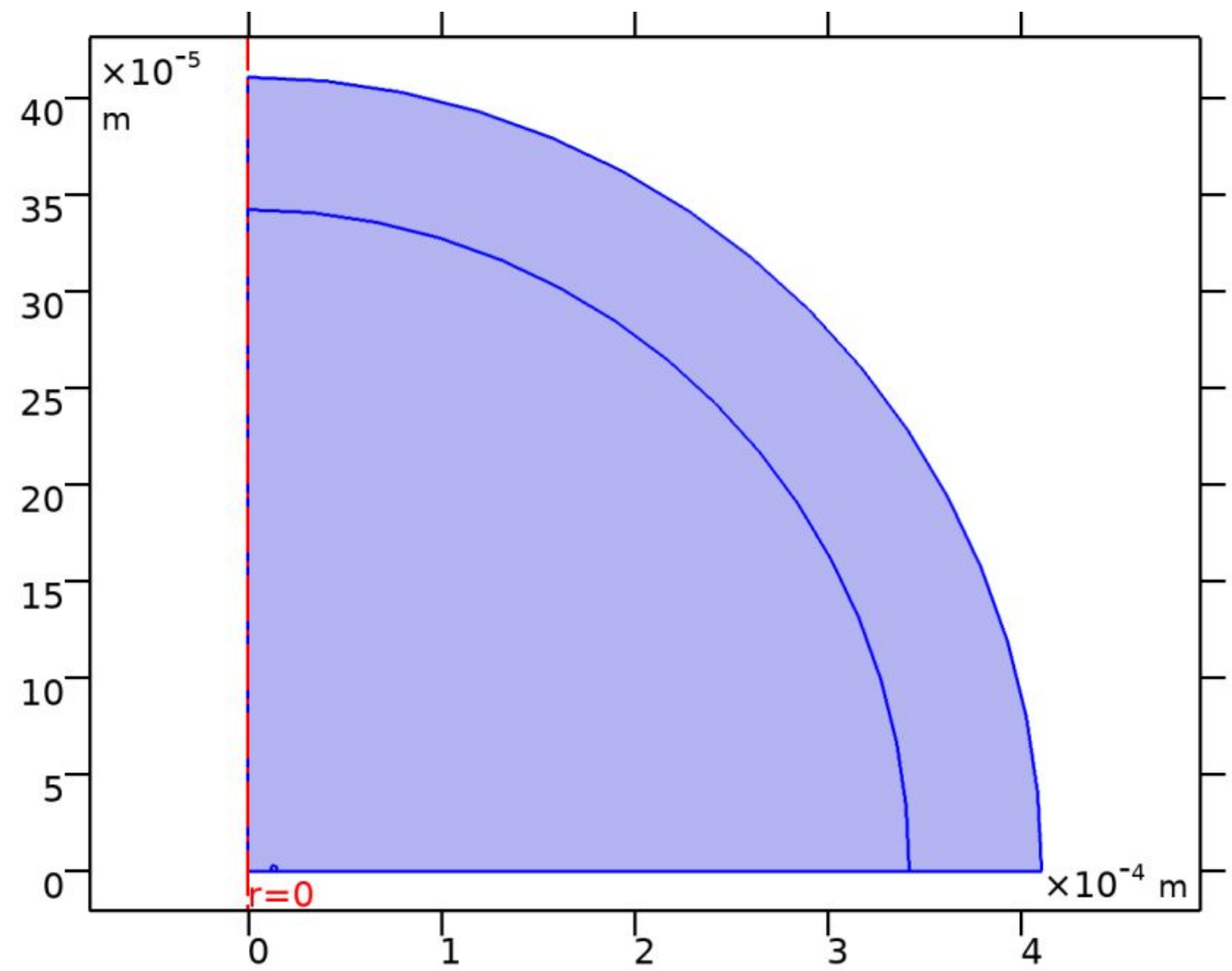

Transport Properties 1

SELECTION

Geometric entity level	Domain
Selection	Domains 1–2

EQUATIONS

$$\nabla \cdot \mathbf{J}_i = R_i$$

$$\mathbf{J}_i = -D_i \nabla c_i$$

$$\phi_l = 0$$

Diffusion

SETTINGS

Description	Value
Material	None
Diffusion coefficient	User defined
Diffusion coefficient	{{D1, 0, 0}, {0, D1, 0}, {0, 0, D1}}
Diffusion coefficient	User defined
Diffusion coefficient	{{D2, 0, 0}, {0, D2, 0}, {0, 0, D2}}
Coordinate system	Global coordinate system
Temperature	User defined

Description	Value
Temperature	293.15[K]

Variables

Name	Expression	Unit	Description	Selection	Details
elan.D_Redrr	D1	m²/s	Diffusion coefficient, rr component	Domains 1–2	+ operation
elan.D_Redphir	0	m²/s	Diffusion coefficient, phir component	Domains 1–2	+ operation
elan.D_Redzr	0	m²/s	Diffusion coefficient, zr component	Domains 1–2	+ operation
elan.D_Redrphi	0	m²/s	Diffusion coefficient, rphi component	Domains 1–2	+ operation
elan.D_Redphiphi	D1	m²/s	Diffusion coefficient, phiphi component	Domains 1–2	+ operation
elan.D_Redzphi	0	m²/s	Diffusion coefficient, zphi component	Domains 1–2	+ operation
elan.D_Redrz	0	m²/s	Diffusion coefficient, rz component	Domains 1–2	+ operation
elan.D_Redphiz	0	m²/s	Diffusion coefficient, phiz component	Domains 1–2	+ operation
elan.D_Redzz	D1	m²/s	Diffusion coefficient, zz component	Domains 1–2	+ operation
elan.D_Oxirr	D2	m²/s	Diffusion coefficient, rr component	Domains 1–2	+ operation
elan.D_Oxiphir	0	m²/s	Diffusion coefficient, phir component	Domains 1–2	+ operation
elan.D_Oxizr	0	m²/s	Diffusion coefficient, zr component	Domains 1–2	+ operation

Name	Expression	Unit	Description	Selection	Details
elan.D_Oxirphi	0	m²/s	Diffusion coefficient, rphi component	Domains 1–2	+ operation
elan.D_Oxiphiphi	D2	m²/s	Diffusion coefficient, phiphi component	Domains 1–2	+ operation
elan.D_Oxizphi	0	m²/s	Diffusion coefficient, zphi component	Domains 1–2	+ operation
elan.D_Oxirz	0	m²/s	Diffusion coefficient, rz component	Domains 1–2	+ operation
elan.D_Oxiphiz	0	m²/s	Diffusion coefficient, phiz component	Domains 1–2	+ operation
elan.D_Oxizz	D2	m²/s	Diffusion coefficient, zz component	Domains 1–2	+ operation
elan.Dav_Red	0.5*(elan.D_Redrr+elan.D_Redzz)	m²/s	Average diffusion coefficient	Domains 1–2	
elan.Dav_Oxi	0.5*(elan.D_Oxirr+elan.D_Oxizz)	m²/s	Average diffusion coefficient	Domains 1–2	
elan.tflux_Redr	elan.dflux_Redr	mol/(m²·s)	Total flux, r component	Domains 1–2	+ operation
elan.tflux_Redphi	elan.dflux_Redphi	mol/(m²·s)	Total flux, phi component	Domains 1–2	+ operation
elan.tflux_Redz	elan.dflux_Redz	mol/(m²·s)	Total flux, z component	Domains 1–2	+ operation
elan.dfluxMag_Red	sqrt(elan.dflux_Redr^2+elan.dflux_Redphi^2+elan.dflux_Redz^2)	mol/(m²·s)	Diffusive flux magnitude	Domains 1–2	
elan.tfluxMag_Red	sqrt(elan.tflux_Redr^2+elan.tflux_Redphi^2+elan.tflux_Redz^2)	mol/(m²·s)	Total flux magnitude	Domains 1–2	
elan.tflux_Oxir	elan.dflux_Oxir	mol/(m²·s)	Total flux, r component	Domains 1–2	+ operation

Name	Expression	Unit	Description	Selection	Details
elan.tflux_Oxiphi	elan.dflux_Oxiphi	mol/(m²·s)	Total flux, phi component	Domains 1–2	+ operation
elan.tflux_Oxiz	elan.dflux_Oxiz	mol/(m²·s)	Total flux, z component	Domains 1–2	+ operation
elan.dfluxMag_Oxi	sqrt(elan.dflux_Oxir^2+elan.dflux_Oxiphi^2+elan.dflux_Oxiz^2)	mol/(m²·s)	Diffusive flux magnitude	Domains 1–2	
elan.tfluxMag_Oxi	sqrt(elan.tflux_Oxir^2+elan.tflux_Oxiphi^2+elan.tflux_Oxiz^2)	mol/(m²·s)	Total flux magnitude	Domains 1–2	
elan.dflux_Redr	-elan.D_Redrr*Redr-elan.D_Redrz*Redz	mol/(m²·s)	Diffusive flux, r component	Domains 1–2	
elan.dflux_Redphi	-elan.D_Redphir*Redr-elan.D_Redphiz*Redz	mol/(m²·s)	Diffusive flux, phi component	Domains 1–2	
elan.dflux_Redz	-elan.D_Redzr*Redr-elan.D_Redzz*Redz	mol/(m²·s)	Diffusive flux, z component	Domains 1–2	
elan.grad_Redr	Redr	mol/m⁴	Concentration gradient, r component	Domains 1–2	
elan.grad_Redphi	0	mol/m⁴	Concentration gradient, phi component	Domains 1–2	
elan.grad_Redz	Redz	mol/m⁴	Concentration gradient, z component	Domains 1–2	
elan.dflux_Oxir	-elan.D_Oxirr*Oxir-elan.D_Oxirz*Oxiz	mol/(m²·s)	Diffusive flux, r component	Domains 1–2	
elan.dflux_Oxiphi	-elan.D_Oxiphir	mol/(m²·s)	Diffusive flux, phi component	Domains 1–2	

Name	Expression	Unit	Description	Selection	Details
	*Oxir-elan.D_Oxiphiz*Oxiz				
elan.dflux_Oxiz	-elan.D_Oxizr*Oxir-elan.D_Oxizz*Oxiz	mol/(m²·s)	Diffusive flux, z component	Domains 1–2	
elan.grad_Oxir	Oxir	mol/m⁴	Concentration gradient, r component	Domains 1–2	
elan.grad_Oxiphi	0	mol/m⁴	Concentration gradient, phi component	Domains 1–2	
elan.grad_Oxiz	Oxiz	mol/m⁴	Concentration gradient, z component	Domains 1–2	
elan.phil	0	V	Electrolyte potential	Domains 1–2	
elan.Res_Red	-elan.R_Red	mol/(m³·s)	Equation residual	Domains 1–2	
elan.Res_Oxi	-elan.R_Oxi	mol/(m³·s)	Equation residual	Domains 1–2	

Shape functions

Name	Shape function	Unit	Description	Shape frame	Selection
Red	Lagrange (Linear)	mol/m³	Concentration	Material	Domains 1–2
Oxi	Lagrange (Linear)	mol/m³	Concentration	Material	Domains 1–2

Weak expressions

Weak expression	Integration order	Integration frame	Selection
2*(elan.dflux_Redr*test(Redr)+elan.dflux_Redz*test(Redz))*pi*r	2	Material	Domains 1–2
2*(elan.dflux_Oxir*test(Oxir)+elan.dflux_Oxiz*test(Oxiz))*pi*r	2	Material	Domains 1–2
2*elan.streamline*(isScalingSystemDomain==0)*pi*r	2	Material	Domains 1–2
2*elan.crosswind*(isScalingSystemDomain==0)*pi*r	4	Material	Domains 1–2

2.3.4 Axial Symmetry 1

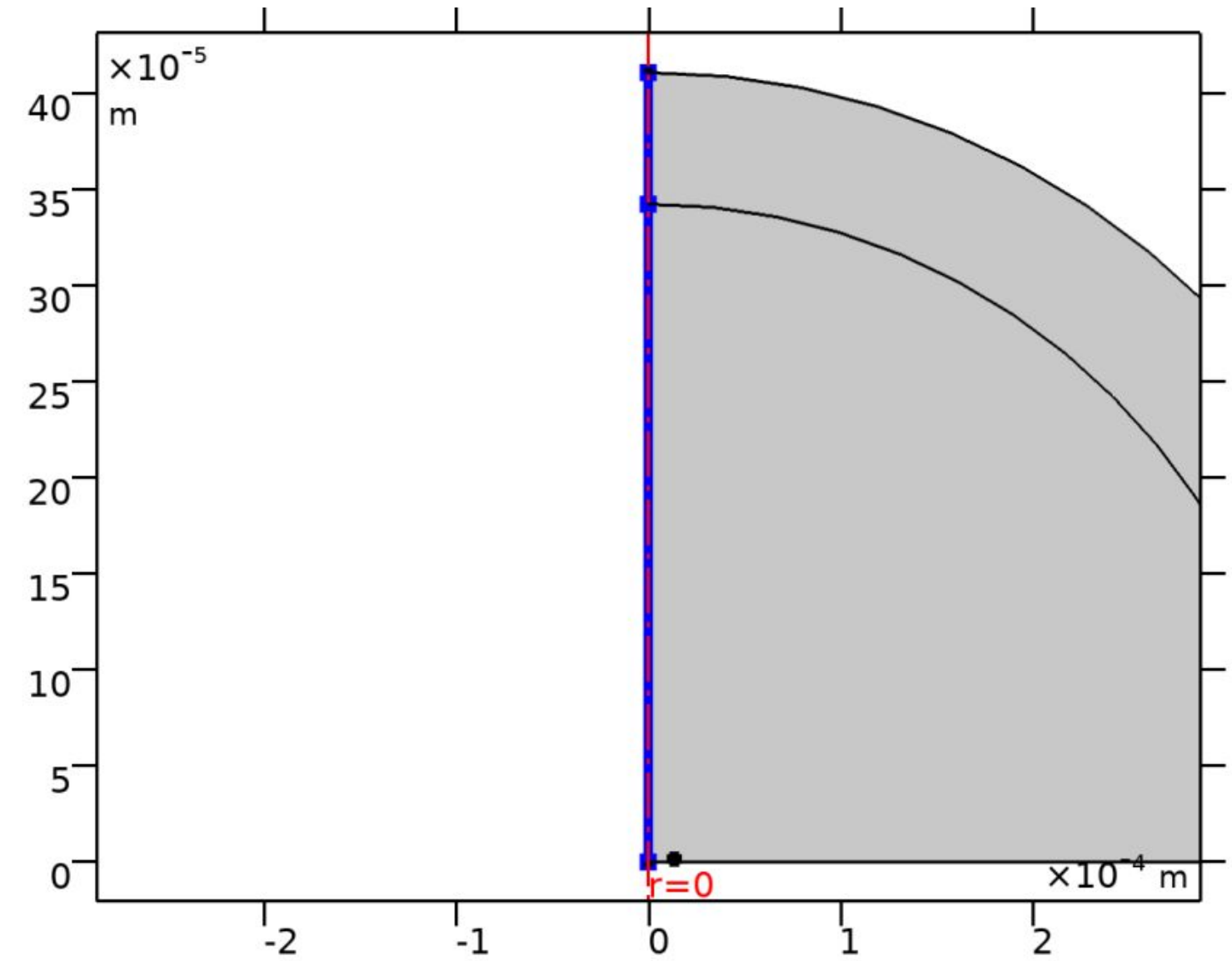

Axial Symmetry 1

SELECTION

Geometric entity level	Boundary
Selection	Boundaries 1, 3

2.3.5 No Flux 1

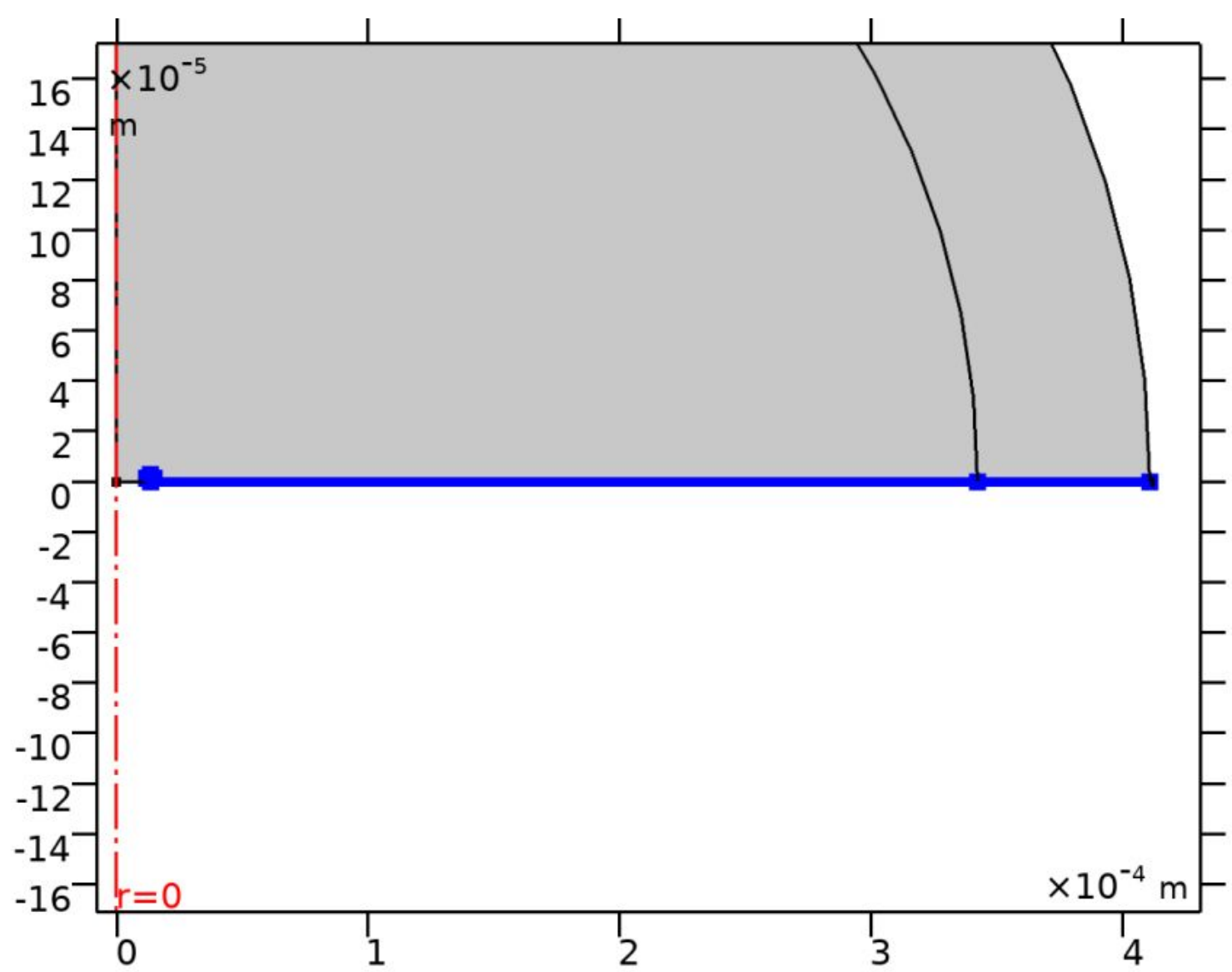

No Flux 1

SELECTION

Geometric entity level	Boundary

Selection	Boundaries 4–6, 9–12

EQUATIONS

$$-\mathbf{n}\cdot\mathbf{J}_i = 0$$

2.3.6 Initial Values 1

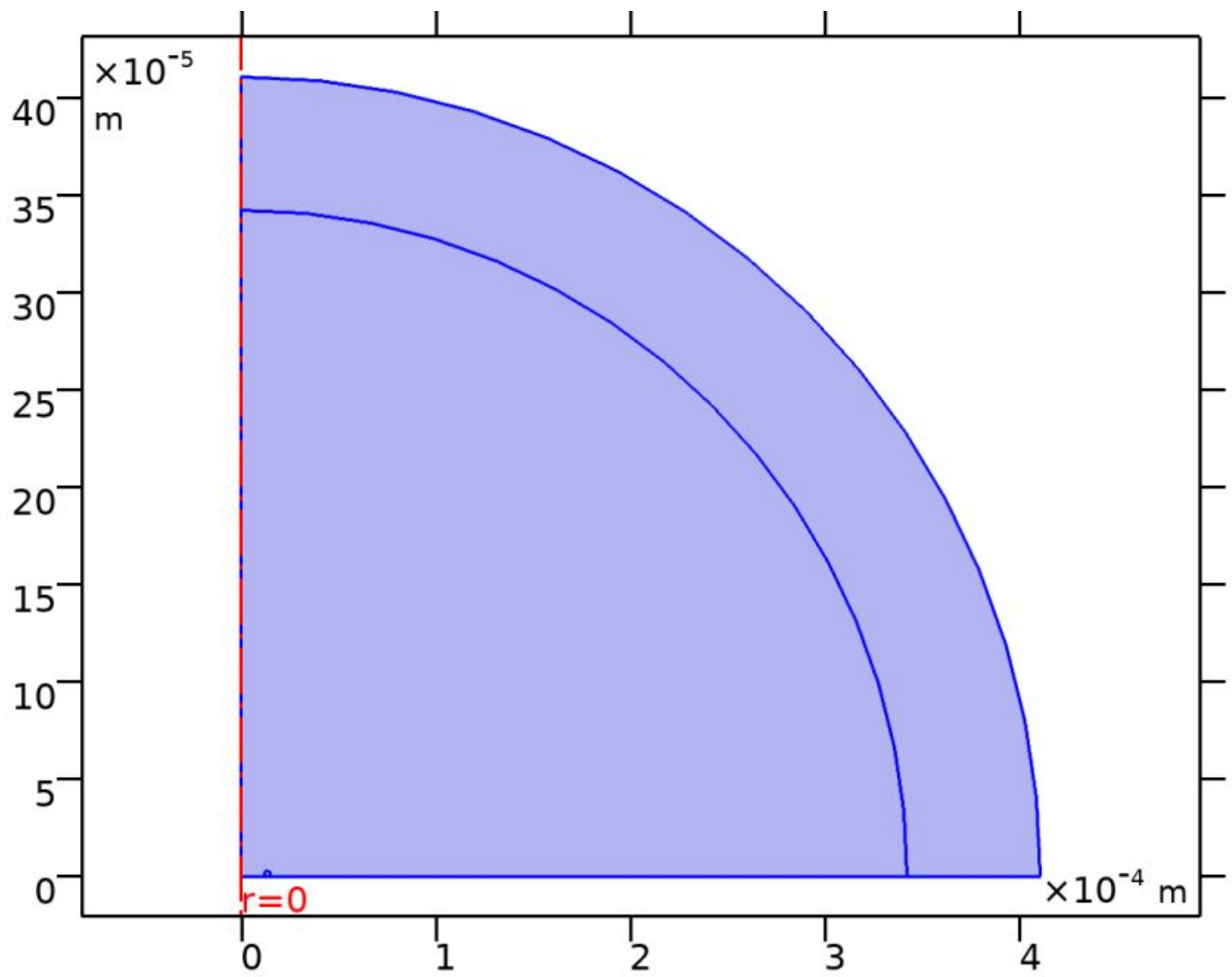

Initial Values 1

SELECTION

Geometric entity level	Domain
Selection	Domains 1–2

Initial values

SETTINGS

Description	Value
Concentration	{c_bulk, 0}

2.3.7 Electrode Surface 1

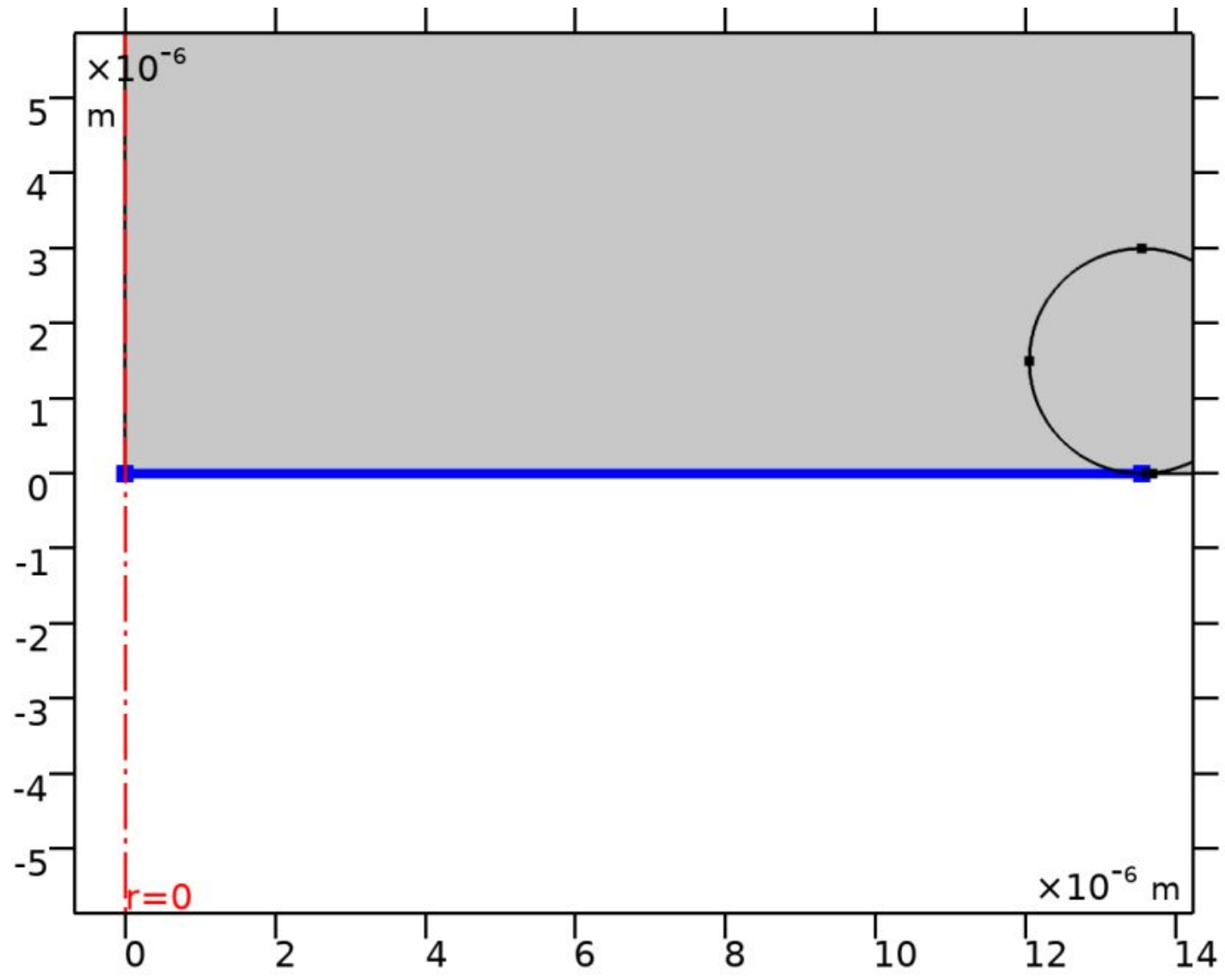

Electrode Surface 1

SELECTION

Geometric entity level	Boundary
Selection	Boundary 2

EQUATIONS

$$i_{\mathrm{total}} = \sum_m i_{\mathrm{loc},m}$$

$$-\mathbf{n} \cdot \mathbf{J}_i = R_{i,\mathrm{tot}}, \quad R_{i,\mathrm{tot}} = \sum_m R_{i,m}$$

Dissolving-depositing species

SETTINGS

Description	Value
Species	
Solve for surface concentration variables	On
Film resistance	No film resistance
Perturbation amplitude	0
Boundary condition	Electrode potential
Electrode potential vs. reference	E_appl
Electric reference potential	User defined
Electric reference potential	0

Variables

Name	Expression	Unit	Description	Selection	Details
elan.nil	elan.itot	A/m²	Inward electrolyte current density	Boundary 2	+ operation
elan.Evsref0	E_appl	V	Electrode potential vs. reference	Boundary 2	
elan.phisext	elan.Evsref0	V	External electric potential	Boundary 2	
elan.phis_els1	elan.els1.int(elan.phisext*elan.dvolfactor)/elan.Area_els1	V	Electric potential	Global	
elan.dvolfactor	2*pi*r	m	Differential volume factor	Boundary 2	
elan.Area_els1	elan.els1.int(elan.dvolfactor)	m²	Area	Global	
elan.itotavg_els1	elan.els1.int(elan.itot*elan.dvolfactor)/elan.Area_els1	A/m²	Average total interface current density	Global	
elan.Temp	elan.els1.minput_temperature	K	Temperature	Boundary 2	
elan.Evsref	elan.phisext-elan.phil	V	Electrode potential vs. adjacent reference	Boundary 2	
elan.Ect	elan.phisext-elan.phil	V	Electrode potential	Boundary 2	
elan.itot	0	A/m²	Total interface current density	Boundary 2	+ operation
elan.Itot_els1	elan.els1.int(elan.itot*elan.dvolfactor)	A	Total current	Global	
elan.rhos	8960	kg/m³	Density	Boundary 2	
elan.Ms	0.06355	kg/mol	Molar mass	Boundary 2	

Electrode Reaction 1

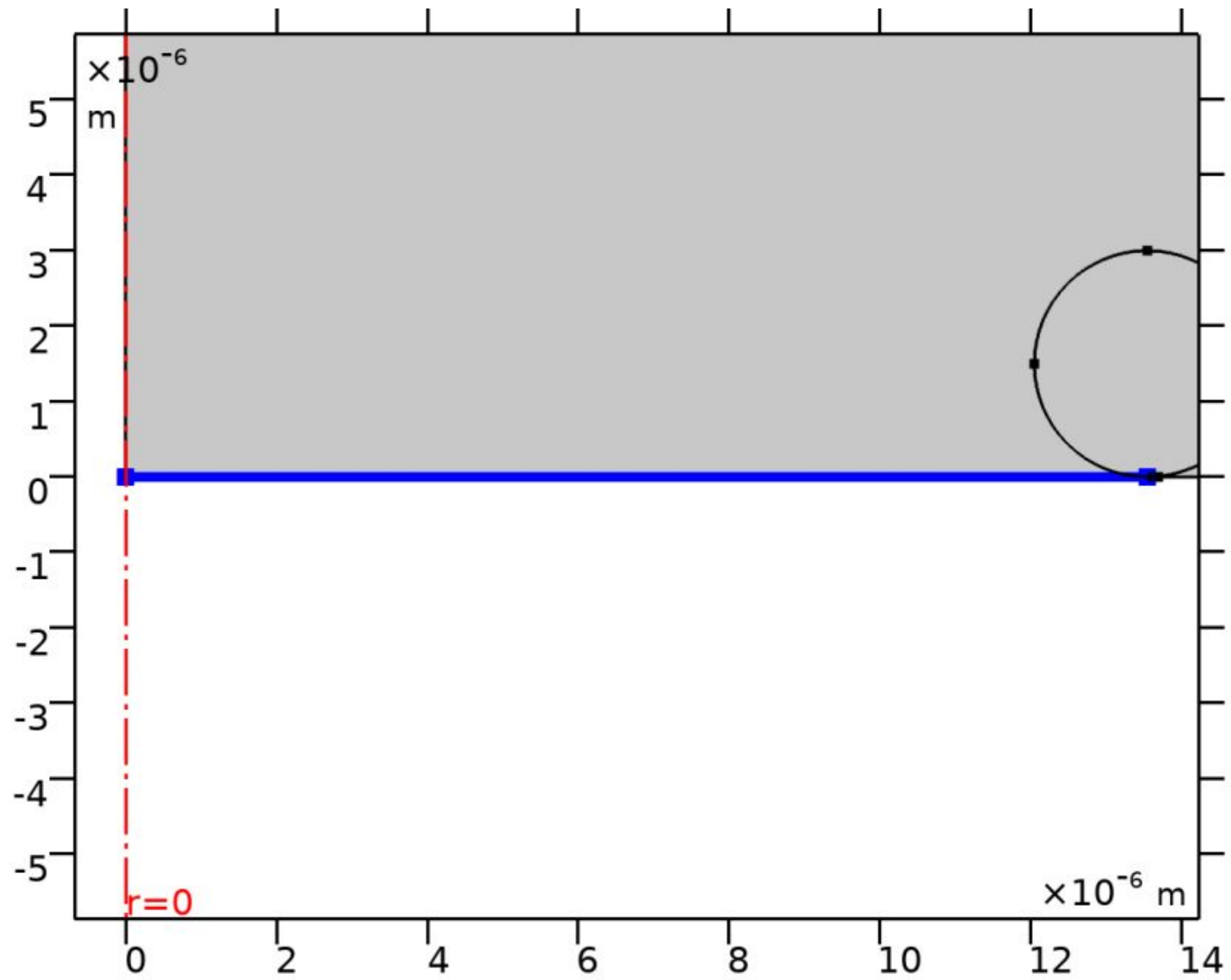

Electrode Reaction 1

SELECTION

Geometric entity level	Boundary
Selection	Boundary 2

EQUATIONS

$$\eta = \phi_{s,ext} - \phi_l - E_{eq}$$

Equilibrium potential

SETTINGS

Description	Value
Equilibrium potential	User defined
Equilibrium potential	Ef
Kinetics expression type	Electroanalytical Butler - Volmer
Heterogeneous rate constant	0.01[m/s]
Cathodic transfer coefficient	0.5
Number of participating electrons	1
Stoichiometric coefficient	{1, -1}
Specify	Temperature derivative
Temperature derivative of equilibrium potential	User defined
Temperature derivative of equilibrium potential	0
Temperature	User defined
Temperature	293.15[K]

Variables

Name	Expression	Unit	Description	Selection	Details
elan.itot	elan.iloc_er1	A/m²	Total interface current density	Boundary 2	+ operation
elan.Eeq_er1	Ef	V	Equilibrium potential	Boundary 2	
elan.alphac_er1	0.5	1	Cathodic transfer coefficient	Boundary 2	
elan.k0_er1	0.01[m/s]	m/s	Heterogeneous rate constant	Boundary 2	
elan.iloc_er1	F_const*elan.k0_er1*(elan.cred_er1*exp((1-elan.alphac_er1)*F_const*elan.eta_er1/(R_const*elan.els1.er1.minput_temperature))-elan.cox_er1*exp(-elan.alphac_er1*F_const*elan.eta_er1/(R_const*elan.els1.er1.minput_temperature)))	A/m²	Local current density	Boundary 2	
elan.els1.er1.iloc	elan.iloc_er1	A/m²	Local current density, Electrode Reaction 1	Boundary 2	
elan.dEeqdT_er1	0	V/K	Temperature derivative of equilibrium potential	Boundary 2	
elan.els1.er1.N0_Red	-elan.iloc_er1/F_const	mol/(m²·s)	Inward flux	Boundary 2	
elan.cred_er1	Red	mol/m³	Reduced species concentration	Boundary 2	+ operation

Name	Expression	Unit	Description	Selection	Details
elan.cox_er1	Oxi	mol/m³	Oxidized species concentration	Boundary 2	+ operation
elan.els1.er1.N0_Oxi	elan.iloc_er1/F_const	mol/(m²·s)	Inward flux	Boundary 2	
elan.Qirrev_er1	elan.iloc_er1*elan.eta_er1	W/m²	Irreversible heat flux	Boundary 2	
elan.Qrev_er1	elan.iloc_er1*elan.els1.er1.minput_temperature*elan.dEeqdT_er1	W/m²	Reversible heat flux	Boundary 2	
elan.els1.er1.Qb	elan.Qrev_er1+elan.Qirrev_er1	W/m²	Electrochemical reaction boundary heat source	Boundary 2	
elan.Qbtot	elan.els1.er1.Qb	W/m²	Electrochemical reaction boundary heat source	Boundary 2	+ operation
elan.eta_er1	elan.Ect-elan.Eeq_er1	V	Overpotential	Boundary 2	

Weak expressions

Weak expression	Integration order	Integration frame	Selection
2*elan.els1.er1.N0_Red*test(Red)*pi*r	2	Material	Boundary 2
2*elan.els1.er1.N0_Oxi*test(Oxi)*pi*r	2	Material	Boundary 2

2.3.8 Concentration 1

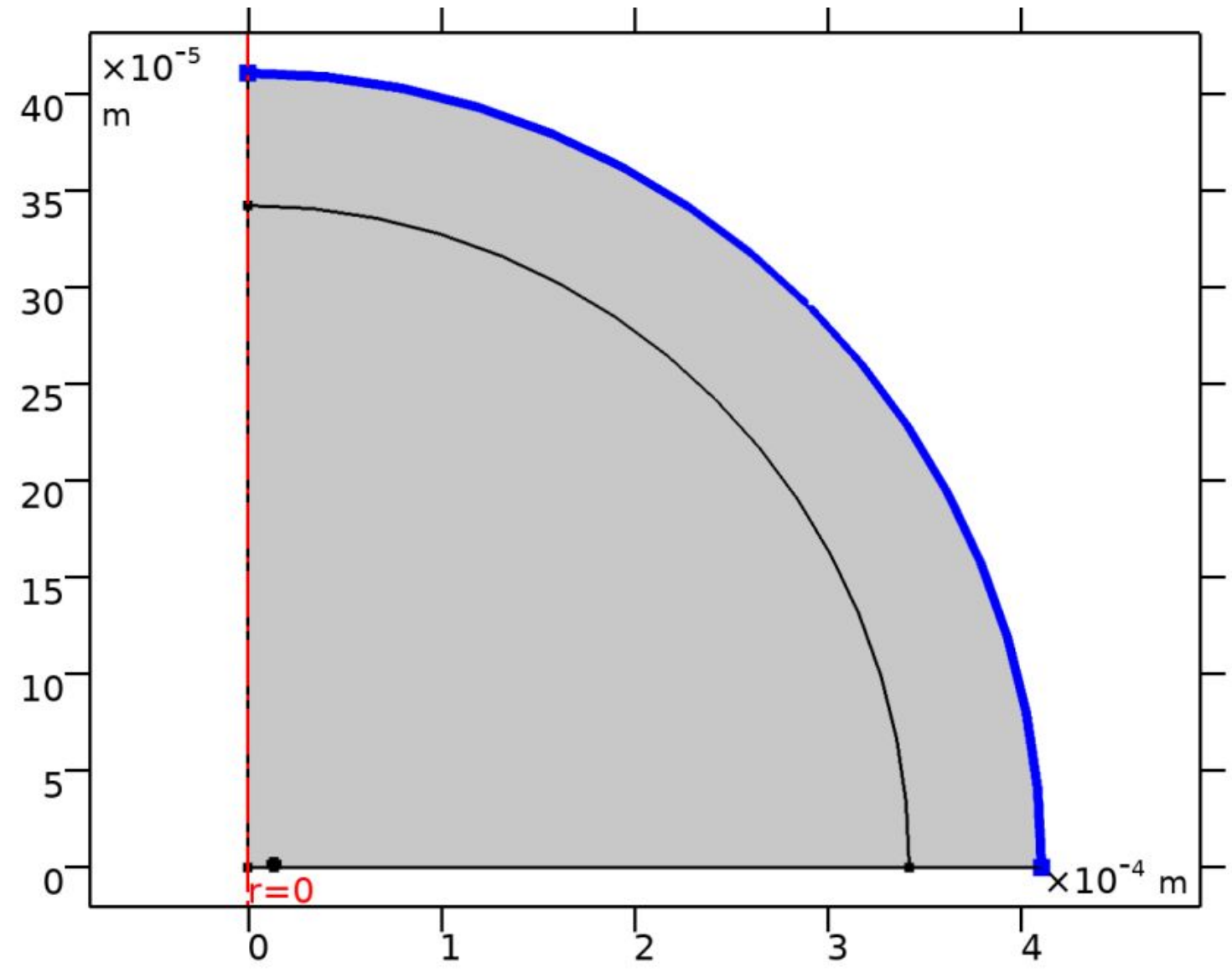

Concentration 1

SELECTION

Geometric entity level	Boundary
Selection	Boundary 8

EQUATIONS

$$c_i = c_{0,j}$$

Concentration

SETTINGS

Description	Value
Species Red	On
Species Oxi	On
Concentration	{c_bulk, 0}

Variables

Name	Expression	Unit	Description	Selection
elan.c0_Red	c_bulk	mol/m³	Concentration	Boundary 8
elan.c0_Oxi	0	mol/m³	Concentration	Boundary 8

Constraints

Constraint	Constraint force	Shape function	Selection	Details
-Red+elan.c0_Red	test(-Red+elan.c0_Red)	Lagrange (Linear)	Boundary 8	Elemental
-Oxi+elan.c0_Oxi	test(-Oxi+elan.c0_Oxi)	Lagrange (Linear)	Boundary 8	Elemental

2.4 MESH 1

MESH STATISTICS

Description	Value
Minimum element quality	4.788E-4
Average element quality	0.984
Triangle	8832
Quad	1261
Edge element	493
Vertex element	10

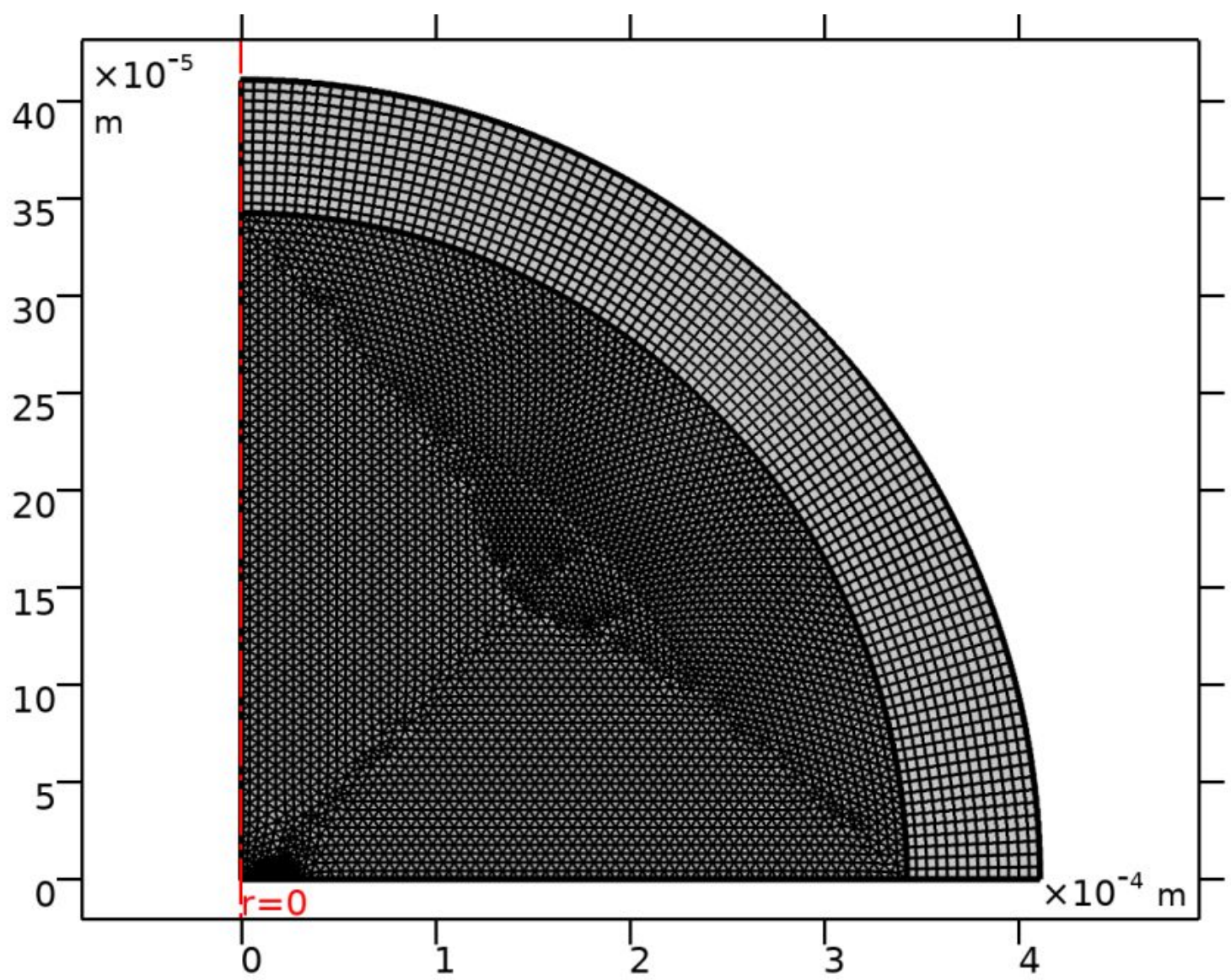

Mesh 1

2.4.1 Size (size)

SETTINGS

Description	Value
Maximum element size	5.55E-6
Minimum element size	1.88E-8
Curvature factor	0.25
Maximum element growth rate	1.3
Predefined size	Finer
Custom element size	Custom

2.4.2 Free Triangular 1 (ftri1)

SELECTION

Geometric entity level	Domain
Selection	Domain 1

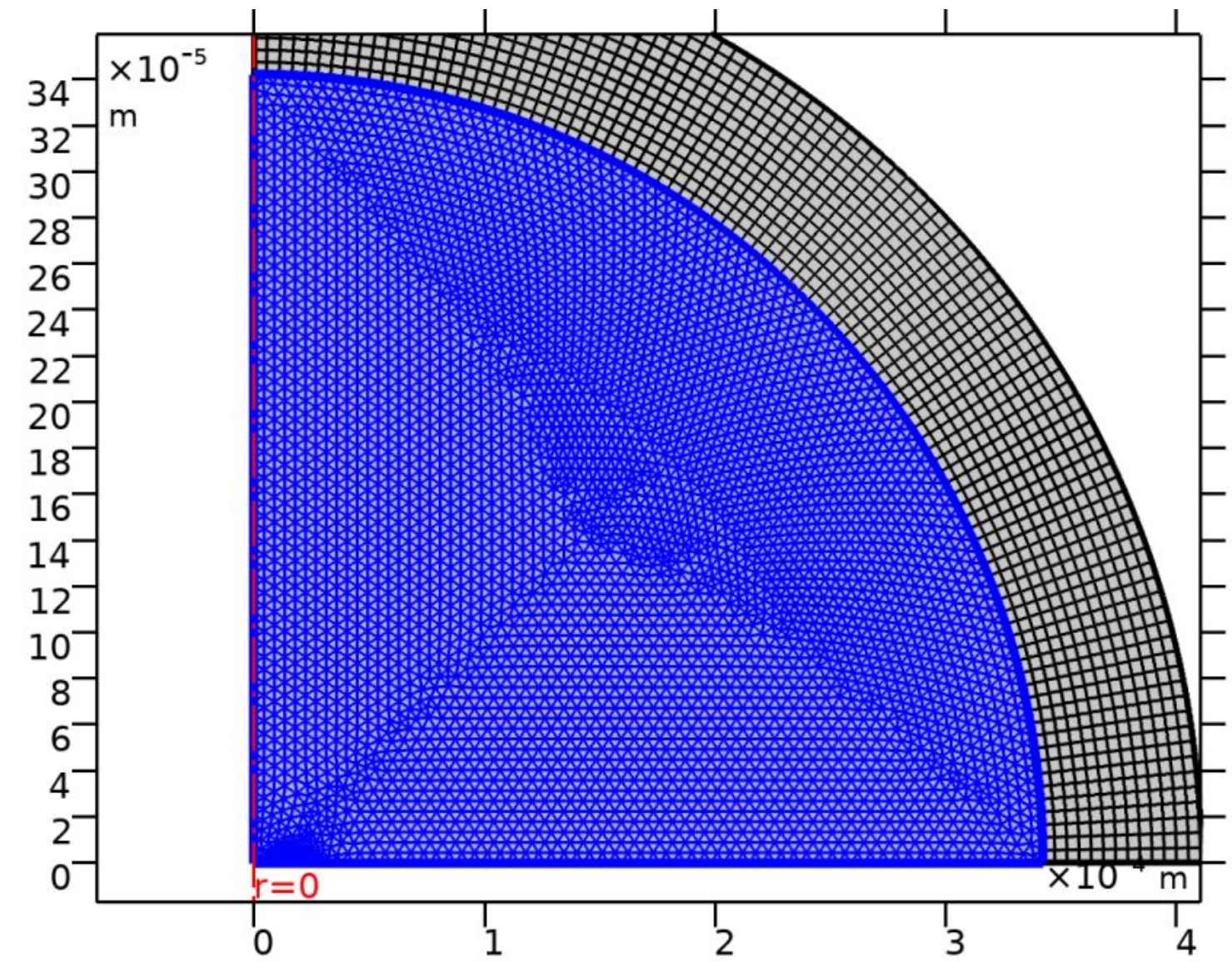

Free Triangular 1

Size 1 (size1)

SELECTION

Geometric entity level	Point
Selection	Point 7

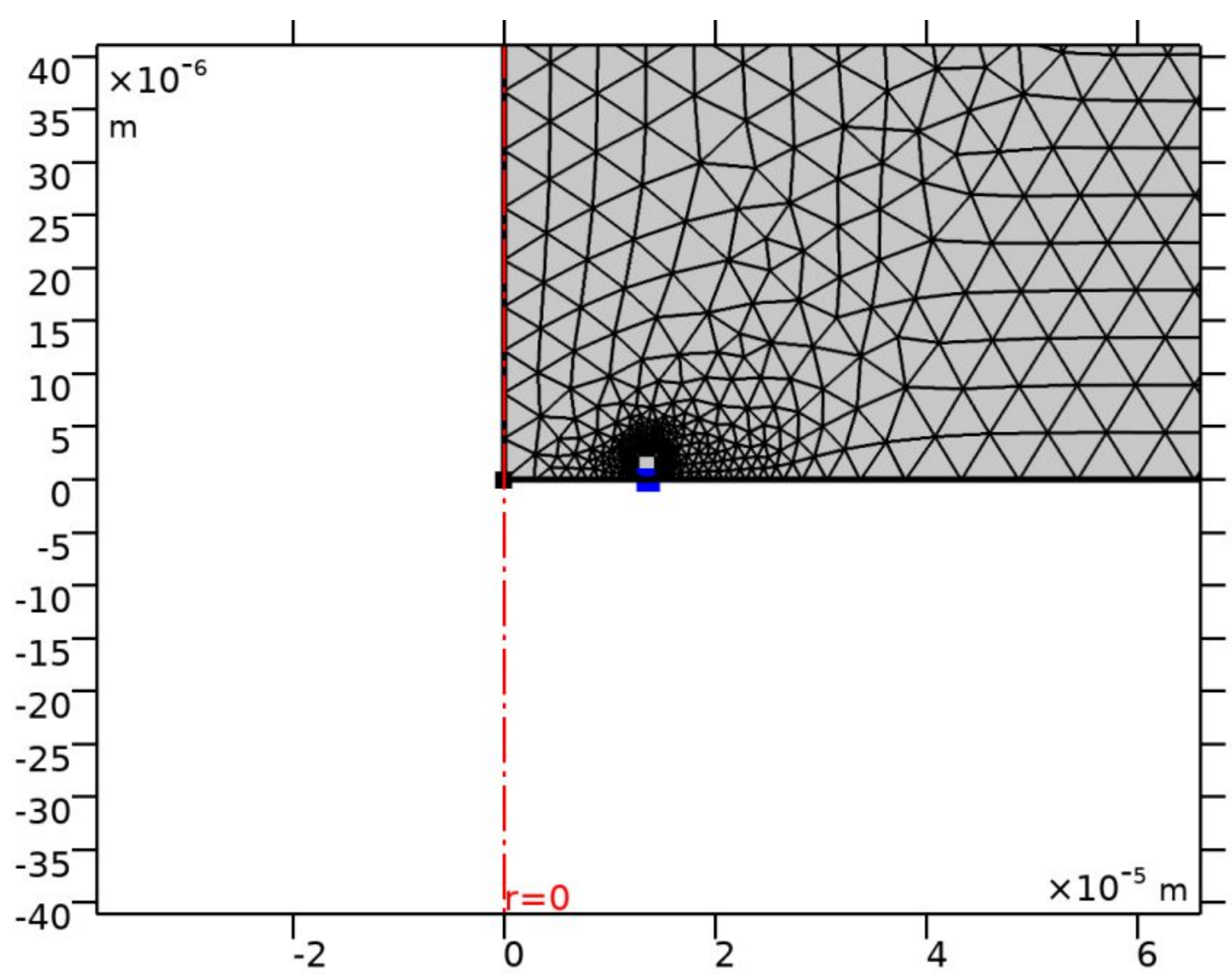

Size 1

SETTINGS

**Description**	**Value**
Maximum element size	re/100
Minimum element size	4.5E-8

Description	Value
Minimum element size	Off
Curvature factor	0.3
Curvature factor	Off
Resolution of narrow regions	Off
Maximum element growth rate	1.3
Maximum element growth rate	Off
Custom element size	Custom

2.4.3 Mapped 1 (map1)

SELECTION

Geometric entity level	Domain
Selection	Domain 2

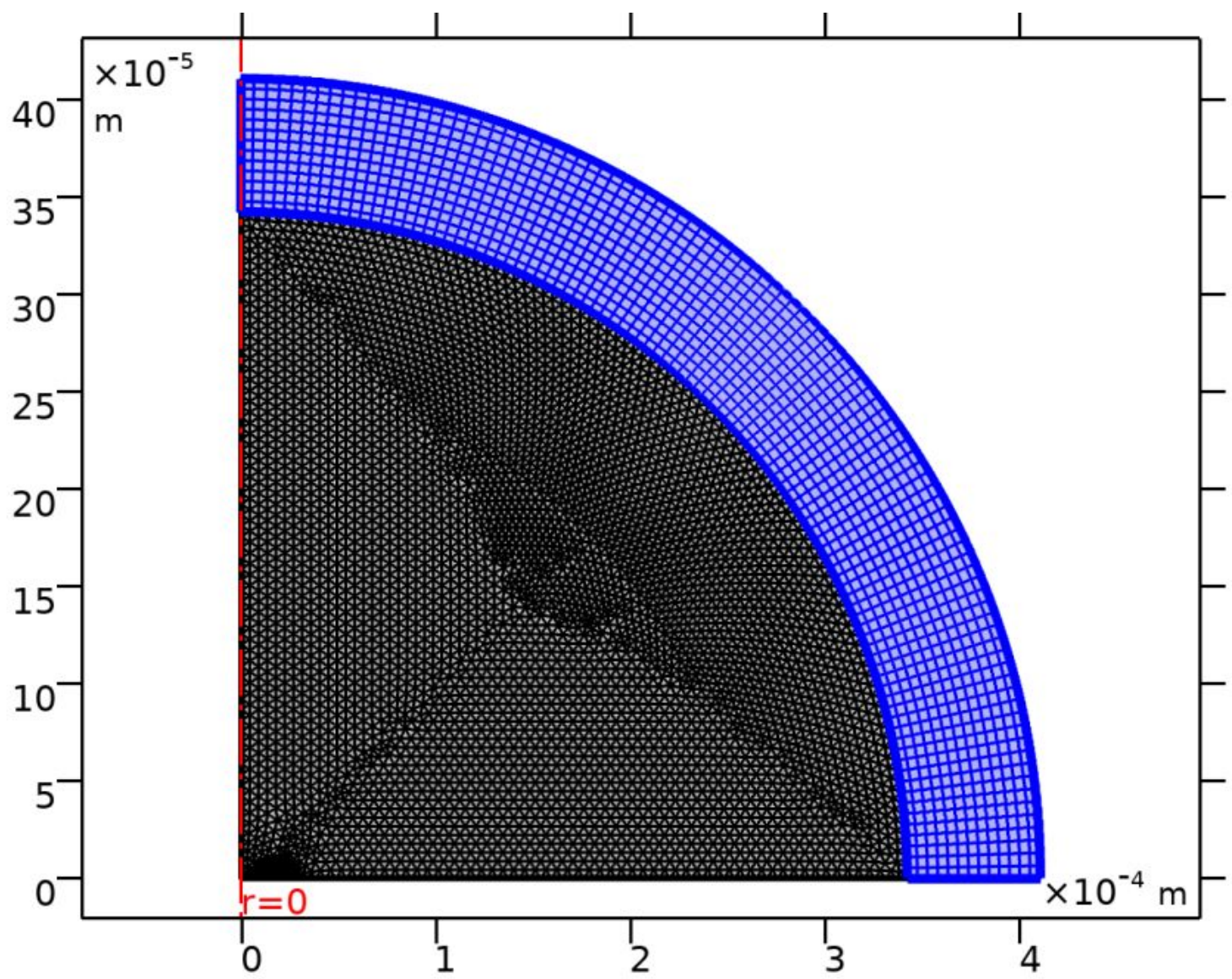

Mapped 1

3 Study 1

COMPUTATION INFORMATION

Computation time	1 min 34 s
CPU	AMD64 Family 21 Model 101 Stepping 1, 4 cores
Operating system	Windows 10

3.1 PARAMETRIC SWEEP

Parameter name	Parameter value list	Parameter unit
E_appl	range(E_start, E_step, E_vertex)	V

STUDY SETTINGS

Description	Value
Sweep type	Specified combinations
Parameter name	E_appl
Unit	V

PARAMETERS

Parameter name	Parameter value list	Parameter unit
E_appl (Applied potential)	range(E_start, E_step, E_vertex)	V

3.2 STATIONARY

STUDY SETTINGS

Description	Value
Include geometric nonlinearity	Off

MESH SELECTION

Geometry	Mesh
mesh1	mesh1

PHYSICS AND VARIABLES SELECTION

Physics interface	Discretization
Electroanalysis (elan)	physics

MESH SELECTION

Geometry	Mesh
Geometry 1 (geom1)	mesh1

3.3 SOLVER CONFIGURATIONS

3.3.1 Solution 1

Compile Equations: Stationary (st1)

STUDY AND STEP

Description	Value
Use study	Study 1
Use study step	Stationary

LOG

```
<---- Compile Equations: Stationary in Study 1/Solution 1 (sol1) ----------------
Started at Aug 7, 2026 9:31:14 AM.
Geometry shape order: Linear
Running on AMD64 Family 21 Model 101 Stepping 1, AuthenticAMD.
Using 1 socket with 4 cores in total on LAPTOP-CSABA14L.
Available memory: 7.57 GB.
Number of vertex elements: 8
Number of boundary elements: 370
Number of elements: 8832
Minimum element quality: 0.01443
Number of vertex elements: 10
Number of boundary elements: 493
Minimum element quality: 0.01443
Time: 1 s.
Physical memory: 1.38 GB
Virtual memory: 1.68 GB
Ended at Aug 7, 2026 9:31:16 AM.
----- Compile Equations: Stationary in Study 1/Solution 1 (sol1) --------------->
```

Dependent Variables 1 (v1)

GENERAL

Description	Value
Defined by study step	Stationary

INITIAL VALUE CALCULATION CONSTANTS

Constant name	Initial value source
E_appl	range(E_start, E_step, E_vertex)

LOG

```
<---- Dependent Variables 1 in Study 1/Solution 1 (sol1) -----------------------
Started at Aug 7, 2026 9:31:16 AM.
Solution time: 0 s.
Physical memory: 1.38 GB
Virtual memory: 1.67 GB
Ended at Aug 7, 2026 9:31:16 AM.
----- Dependent Variables 1 in Study 1/Solution 1 (sol1) ---------------------->
```

Concentration (comp1.Red) (comp1_Red)

GENERAL

Description	Value
Field components	comp1.Red

Concentration (comp1.Oxi) (comp1_Oxi)

GENERAL

Description	Value
Field components	comp1.Oxi

Stationary Solver 1 (s1)

RESULTS WHILE SOLVING

Description	Value
Plot	On
Plot group	Concentration (elan)
Probes	None

LOG

```
<---- Stationary Solver 1 in Study 1/Solution 1 (sol1) ------------------------
Started at Aug 7, 2026 9:31:16 AM.
Parametric solver
Linear solver
Number of degrees of freedom solved for: 11750 (plus 1206 internal DOFs).

Parameter E_appl = -0.4.
Nonsymmetric matrix found.
Scales for dependent variables:
Concentration (comp1.Oxi): 2
Concentration (comp1.Red): 2.5
Orthonormal null-space function used.
Iter      SolEst     Damping    Stepsize #Res #Jac #Sol   LinErr   LinRes
   1        0.61   1.0000000        0.61    1    1    1  2.6e-09  1.2e-13

Parameter E_appl = -0.38.
Iter      SolEst     Damping    Stepsize #Res #Jac #Sol   LinErr   LinRes
   1        0.61   1.0000000        0.61    2    2    2  3.3e-09  7.1e-14

Parameter E_appl = -0.36.
Iter      SolEst     Damping    Stepsize #Res #Jac #Sol   LinErr   LinRes
   1        0.61   1.0000000        0.61    3    3    3  5.1e-09  7.9e-14

Parameter E_appl = -0.34.
Iter      SolEst     Damping    Stepsize #Res #Jac #Sol   LinErr   LinRes
   1        0.61   1.0000000        0.61    4    4    4  4.7e-09  4.8e-14

Parameter E_appl = -0.32.
Iter      SolEst     Damping    Stepsize #Res #Jac #Sol   LinErr   LinRes
   1        0.61   1.0000000        0.61    5    5    5  1.5e-09  2.5e-14

Parameter E_appl = -0.3.
Iter      SolEst     Damping    Stepsize #Res #Jac #Sol   LinErr   LinRes
   1        0.61   1.0000000        0.61    6    6    6  2.9e-10  2.9e-14

Parameter E_appl = -0.28.
Iter      SolEst     Damping    Stepsize #Res #Jac #Sol   LinErr   LinRes
   1        0.61   1.0000000        0.61    7    7    7  1.1e-09  1.6e-14

Parameter E_appl = -0.26.
Iter      SolEst     Damping    Stepsize #Res #Jac #Sol   LinErr   LinRes
   1        0.61   1.0000000        0.61    8    8    8  4.1e-10  1.1e-14

Parameter E_appl = -0.24.
Iter      SolEst     Damping    Stepsize #Res #Jac #Sol   LinErr   LinRes
   1        0.61   1.0000000        0.61    9    9    9  4.6e-10  6.8e-15

Parameter E_appl = -0.22.
Iter      SolEst     Damping    Stepsize #Res #Jac #Sol   LinErr   LinRes
   1        0.61   1.0000000        0.61   10   10   10  3.8e-10  7.5e-15

Parameter E_appl = -0.2.
Iter      SolEst     Damping    Stepsize #Res #Jac #Sol   LinErr   LinRes
   1        0.61   1.0000000        0.61   11   11   11  1.1e-10    4e-15
```

```
Parameter E_appl = -0.18.
Iter      SolEst     Damping    Stepsize #Res #Jac #Sol   LinErr   LinRes
   1        0.61   1.0000000        0.61   12   12   12  8.4e-11  2.2e-15

Parameter E_appl = -0.16.
Iter      SolEst     Damping    Stepsize #Res #Jac #Sol   LinErr   LinRes
   1        0.61   1.0000000        0.61   13   13   13  1.8e-10  1.4e-15

Parameter E_appl = -0.14.
Iter      SolEst     Damping    Stepsize #Res #Jac #Sol   LinErr   LinRes
   1        0.61   1.0000000        0.61   14   14   14  6.3e-10  2.4e-14

Parameter E_appl = -0.12.
Iter      SolEst     Damping    Stepsize #Res #Jac #Sol   LinErr   LinRes
   1        0.61   1.0000000        0.61   15   15   15  2.2e-10  5.1e-15

Parameter E_appl = -0.1.
Iter      SolEst     Damping    Stepsize #Res #Jac #Sol   LinErr   LinRes
   1        0.61   1.0000000        0.61   16   16   16  1.6e-10  8.4e-16

Parameter E_appl = -0.08.
Iter      SolEst     Damping    Stepsize #Res #Jac #Sol   LinErr   LinRes
   1        0.61   1.0000000        0.61   17   17   17  1.2e-11  7.8e-16

Parameter E_appl = -0.06.
Iter      SolEst     Damping    Stepsize #Res #Jac #Sol   LinErr   LinRes
   1        0.61   1.0000000        0.61   18   18   18  7.7e-11  8.8e-15

Parameter E_appl = -0.04.
Iter      SolEst     Damping    Stepsize #Res #Jac #Sol   LinErr   LinRes
   1        0.61   1.0000000        0.61   19   19   19  1.9e-11  1.2e-15

Parameter E_appl = -0.02.
Iter      SolEst     Damping    Stepsize #Res #Jac #Sol   LinErr   LinRes
   1        0.61   1.0000000        0.61   20   20   20  9.8e-11  2.8e-15

Parameter E_appl = 0.
Symmetric matrices found.
Iter      SolEst     Damping    Stepsize #Res #Jac #Sol   LinErr   LinRes
   1        0.62   1.0000000        0.62   21   21   21  7.4e-11  3.8e-16

Parameter E_appl = 0.02.
Nonsymmetric matrix found.
Iter      SolEst     Damping    Stepsize #Res #Jac #Sol   LinErr   LinRes
   1        0.64   1.0000000        0.64   22   22   22  1.7e-11  3.6e-16

Parameter E_appl = 0.04.
Iter      SolEst     Damping    Stepsize #Res #Jac #Sol   LinErr   LinRes
   1        0.72   1.0000000        0.72   23   23   23  4.1e-11  3.4e-16

Parameter E_appl = 0.06.
Iter      SolEst     Damping    Stepsize #Res #Jac #Sol   LinErr   LinRes
   1        0.93   1.0000000        0.93   24   24   24  3.9e-11  3.3e-16

Parameter E_appl = 0.08.
```

```
Iter      SolEst     Damping    Stepsize #Res #Jac #Sol   LinErr   LinRes
   1        0.98   1.0000000        0.98   25   25   25  1.8e-11  3.5e-16

Parameter E_appl = 0.1.
Iter      SolEst     Damping    Stepsize #Res #Jac #Sol   LinErr   LinRes
   1           1   1.0000000           1   26   26   26  6.1e-11  4.9e-16

Parameter E_appl = 0.12.
Iter      SolEst     Damping    Stepsize #Res #Jac #Sol   LinErr   LinRes
   1           1   1.0000000           1   27   27   27  4.6e-11  6.3e-16

Parameter E_appl = 0.14.
Iter      SolEst     Damping    Stepsize #Res #Jac #Sol   LinErr   LinRes
   1           1   1.0000000           1   28   28   28  8.9e-11  1.1e-15

Parameter E_appl = 0.16.
Iter      SolEst     Damping    Stepsize #Res #Jac #Sol   LinErr   LinRes
   1           1   1.0000000           1   29   29   29  1.2e-10  1.7e-15

Parameter E_appl = 0.18.
Iter      SolEst     Damping    Stepsize #Res #Jac #Sol   LinErr   LinRes
   1           1   1.0000000           1   30   30   30    5e-11  4.5e-15

Parameter E_appl = 0.2.
Iter      SolEst     Damping    Stepsize #Res #Jac #Sol   LinErr   LinRes
   1           1   1.0000000           1   31   31   31    3e-10  5.1e-15

Parameter E_appl = 0.22.
Iter      SolEst     Damping    Stepsize #Res #Jac #Sol   LinErr   LinRes
   1           1   1.0000000           1   32   32   32  6.7e-10  6.7e-15

Parameter E_appl = 0.24.
Iter      SolEst     Damping    Stepsize #Res #Jac #Sol   LinErr   LinRes
   1           1   1.0000000           1   33   33   33  9.9e-10  1.4e-14

Parameter E_appl = 0.26.
Iter      SolEst     Damping    Stepsize #Res #Jac #Sol   LinErr   LinRes
   1           1   1.0000000           1   34   34   34  1.8e-09  1.7e-14

Parameter E_appl = 0.28.
Iter      SolEst     Damping    Stepsize #Res #Jac #Sol   LinErr   LinRes
   1           1   1.0000000           1   35   35   35  9.9e-10    2e-14

Parameter E_appl = 0.3.
Iter      SolEst     Damping    Stepsize #Res #Jac #Sol   LinErr   LinRes
   1           1   1.0000000           1   36   36   36  2.5e-09  5.2e-14

Parameter E_appl = 0.32.
Iter      SolEst     Damping    Stepsize #Res #Jac #Sol   LinErr   LinRes
   1           1   1.0000000           1   37   37   37  4.7e-10  4.2e-14

Parameter E_appl = 0.34.
Iter      SolEst     Damping    Stepsize #Res #Jac #Sol   LinErr   LinRes
   1           1   1.0000000           1   38   38   38  2.4e-09  1.3e-13
```

```
Parameter E_appl = 0.36.
Iter      SolEst     Damping    Stepsize #Res #Jac #Sol   LinErr   LinRes
   1           1   1.0000000           1   39   39   39  8.6e-09  1.5e-13

Parameter E_appl = 0.38.
Iter      SolEst     Damping    Stepsize #Res #Jac #Sol   LinErr   LinRes
   1           1   1.0000000           1   40   40   40  1.3e-08  1.3e-13

Parameter E_appl = 0.4.
Iter      SolEst     Damping    Stepsize #Res #Jac #Sol   LinErr   LinRes
   1           1   1.0000000           1   41   41   41  7.7e-09  2.8e-13

Parameter E_appl = 0.42.
Iter      SolEst     Damping    Stepsize #Res #Jac #Sol   LinErr   LinRes
   1           1   1.0000000           1   42   42   42  3.7e-08  3.1e-13

Parameter E_appl = 0.44.
Iter      SolEst     Damping    Stepsize #Res #Jac #Sol   LinErr   LinRes
   1           1   1.0000000           1   43   43   43  5.5e-08  4.6e-13

Parameter E_appl = 0.46.
Iter      SolEst     Damping    Stepsize #Res #Jac #Sol   LinErr   LinRes
   1           1   1.0000000           1   44   44   44  1.6e-08  1.1e-12

Parameter E_appl = 0.48.
Iter      SolEst     Damping    Stepsize #Res #Jac #Sol   LinErr   LinRes
   1           1   1.0000000           1   45   45   45  4.3e-08  1.9e-12

Parameter E_appl = 0.5.
Iter      SolEst     Damping    Stepsize #Res #Jac #Sol   LinErr   LinRes
   1           1   1.0000000           1   46   46   46  1.7e-07  1.7e-12

Parameter E_appl = 0.52.
Iter      SolEst     Damping    Stepsize #Res #Jac #Sol   LinErr   LinRes
   1           1   1.0000000           1   47   47   47  1.2e-06  3.2e-12

Parameter E_appl = 0.54.
Iter      SolEst     Damping    Stepsize #Res #Jac #Sol   LinErr   LinRes
   1           1   1.0000000           1   48   48   48  1.6e-06  4.8e-12

Parameter E_appl = 0.56.
Iter      SolEst     Damping    Stepsize #Res #Jac #Sol   LinErr   LinRes
   1           1   1.0000000           1   49   49   49  1.6e-06  7.6e-12

Parameter E_appl = 0.58.
Iter      SolEst     Damping    Stepsize #Res #Jac #Sol   LinErr   LinRes
   1           1   1.0000000           1   50   50   50  1.8e-06  1.5e-11

Parameter E_appl = 0.6.
Iter      SolEst     Damping    Stepsize #Res #Jac #Sol   LinErr   LinRes
   1           1   1.0000000           1   51   51   51  9.4e-07  1.7e-11

Parameter E_appl = 0.62.
Iter      SolEst     Damping    Stepsize #Res #Jac #Sol   LinErr   LinRes
   1           1   1.0000000           1   52   52   52  4.5e-06  2.9e-11
```

```
Parameter E_appl = 0.64.
Iter      SolEst      Damping     Stepsize #Res #Jac #Sol    LinErr    LinRes
   1           1    1.0000000            1   53   53   53   8.4e-07   1.8e-11

Parameter E_appl = 0.66.
Iter      SolEst      Damping     Stepsize #Res #Jac #Sol    LinErr    LinRes
   1           1    1.0000000            1   54   54   54   1.5e-05   5.9e-11

Parameter E_appl = 0.68.
Iter      SolEst      Damping     Stepsize #Res #Jac #Sol    LinErr    LinRes
   1           1    1.0000000            1   55   55   55     3e-05   6.5e-11

Parameter E_appl = 0.7.
Iter      SolEst      Damping     Stepsize #Res #Jac #Sol    LinErr    LinRes
   1           1    1.0000000            1   56   56   56     7e-05     1e-10

Parameter E_appl = 0.72.
Iter      SolEst      Damping     Stepsize #Res #Jac #Sol    LinErr    LinRes
   1           1    1.0000000            1   57   57   57   2.6e-05   1.2e-10

Parameter E_appl = 0.74.
Iter      SolEst      Damping     Stepsize #Res #Jac #Sol    LinErr    LinRes
   1           1    1.0000000            1   58   58   58   8.7e-05   2.5e-10

Parameter E_appl = 0.76.
Iter      SolEst      Damping     Stepsize #Res #Jac #Sol    LinErr    LinRes
   1           1    1.0000000            1   59   59   59   7.7e-05   3.9e-10

Parameter E_appl = 0.78.
Iter      SolEst      Damping     Stepsize #Res #Jac #Sol    LinErr    LinRes
   1           1    1.0000000            1   60   60   60    0.0003   5.6e-10

Parameter E_appl = 0.8.
Iter      SolEst      Damping     Stepsize #Res #Jac #Sol    LinErr    LinRes
   1           1    1.0000000            1   61   61   61   0.00024   8.1e-10
Solution time: 92 s. (1 minute, 32 seconds)
Physical memory: 1.63 GB
Virtual memory: 1.95 GB
Ended at Aug 7, 2026 9:32:48 AM.
----- Stationary Solver 1 in Study 1/Solution 1 (sol1) ------------------------>
```

Parametric 1 (p1)

GENERAL

Description	Value
Defined by study step	Parametric Sweep
Run continuation for	No parameter

PARAMETERS

Parameter name	Parameter value list	Parameter unit
E_appl	range(E_start, E_step, E_vertex)	V

Fully Coupled 1 (fc1)

GENERAL

Description	Value
Linear solver	Direct 1

METHOD AND TERMINATION

Description	Value
Initial damping factor	0.01
Minimum damping factor	1.0E-6
Maximum number of iterations	50

Direct 1 (d1)

GENERAL

Description	Value
Solver	PARDISO
Preordering algorithm	Nested dissection multithreaded
Out-of-core	Off

ERROR

Description	Value
Factor in error estimate	400

4 Results

4.1 DATA SETS

4.1.1 Study 1/Solution 1

SOLUTION

Description	Value
Solution	Solution 1
Component	Save Point Geometry 1

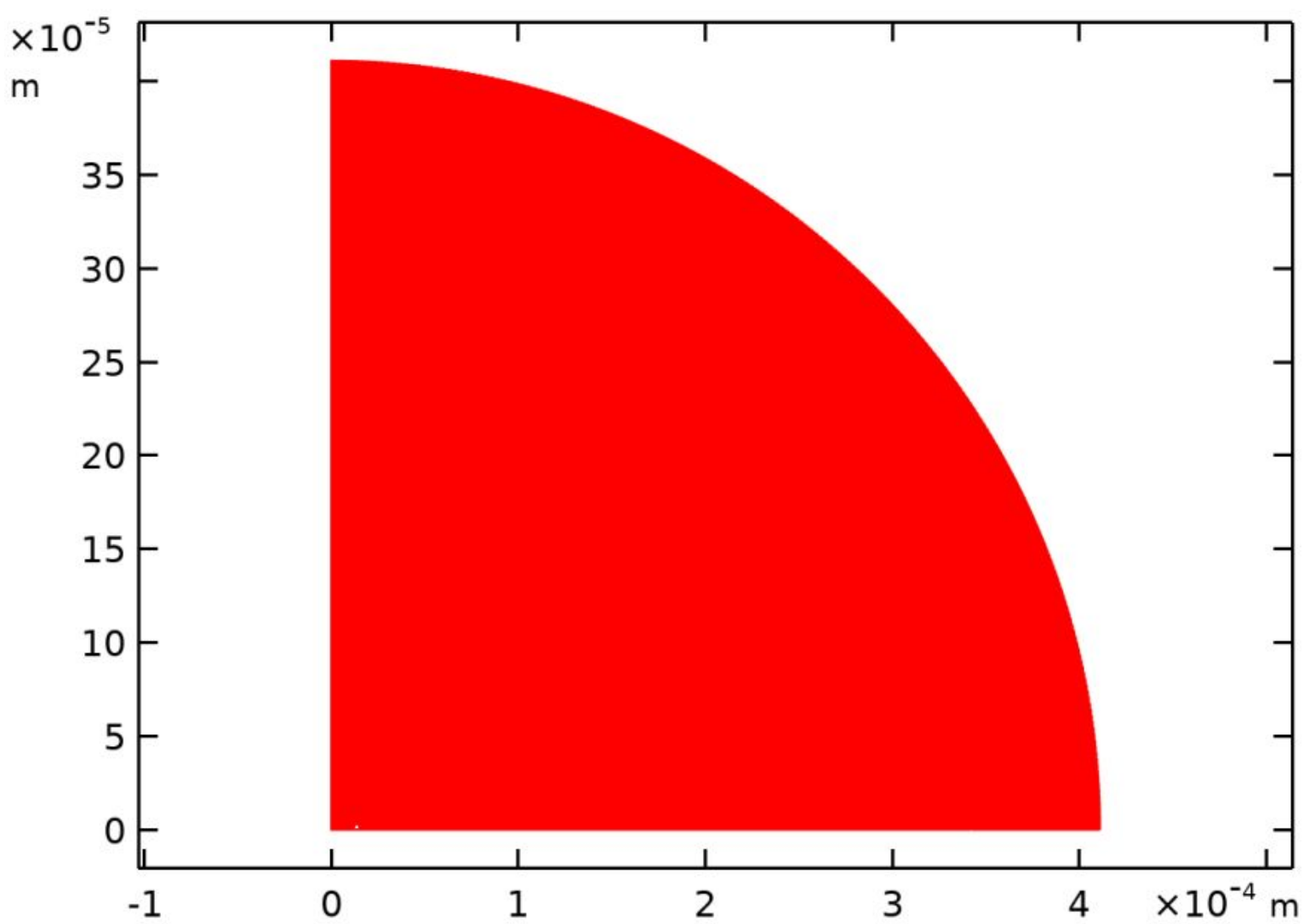

Data set: Study 1/Solution 1

4.1.2 Revolution 2D 1

DATA

Description	Value
Data set	Study 1/Solution 1

AXIS DATA

Description	Value
Axis entry method	Two points
Points	{{0, 0}, {0, 1}}

REVOLUTION LAYERS

Description	Value
Start angle	-90
Revolution angle	225

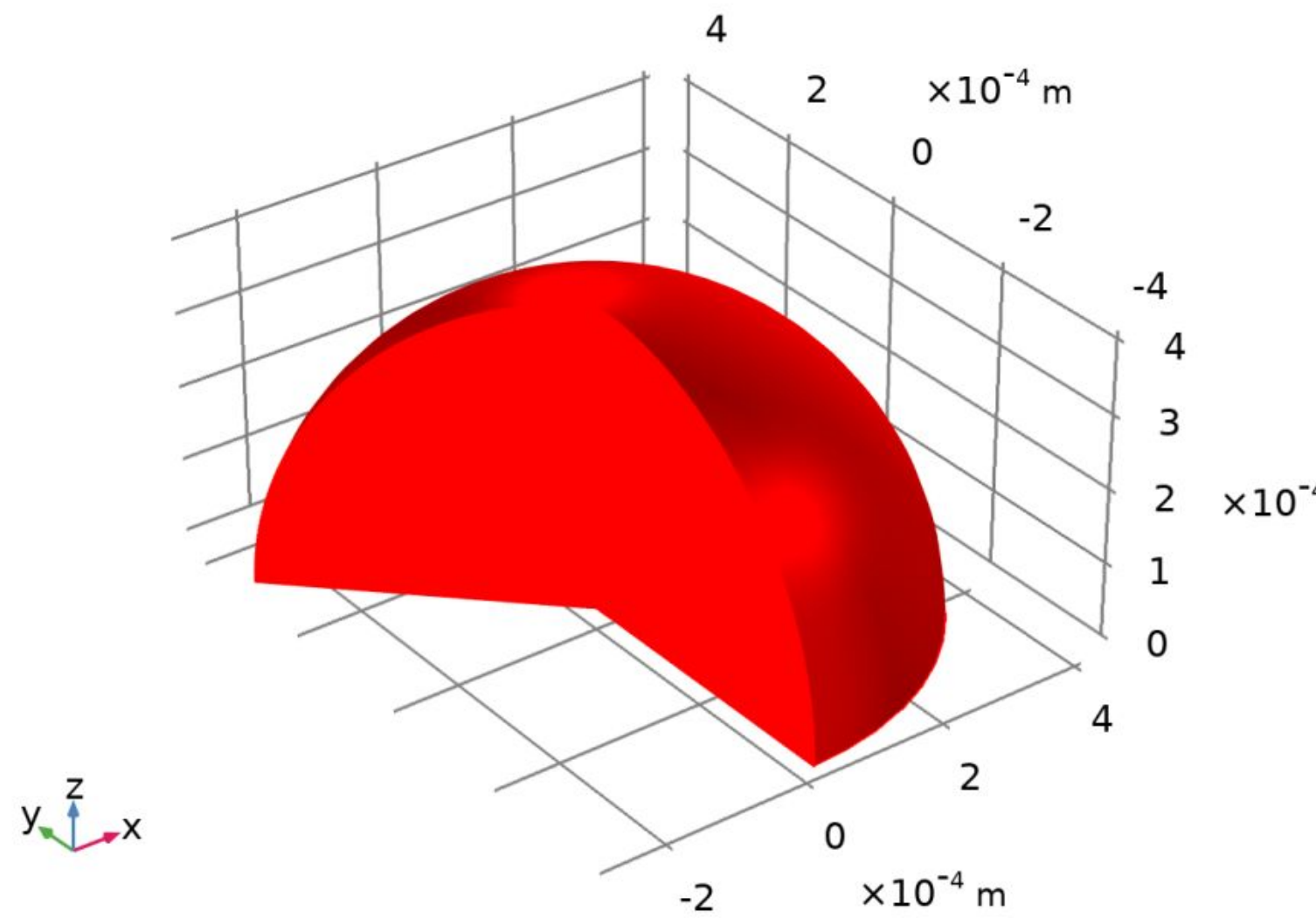

Data set: Revolution 2D 1

4.2 TABLES

4.2.1 Evaluation 3D

Interactive 3D values

x	y	z	Value
-4.1970E-5	4.1970E-5	5.0471E-5	0.99316

4.2.2 Evaluation 2D

Interactive 2D values

x	y	Value
5.7347E-5	3.3544E-5	1.9153

4.3 PLOT GROUPS

4.3.1 Concentration (elan)

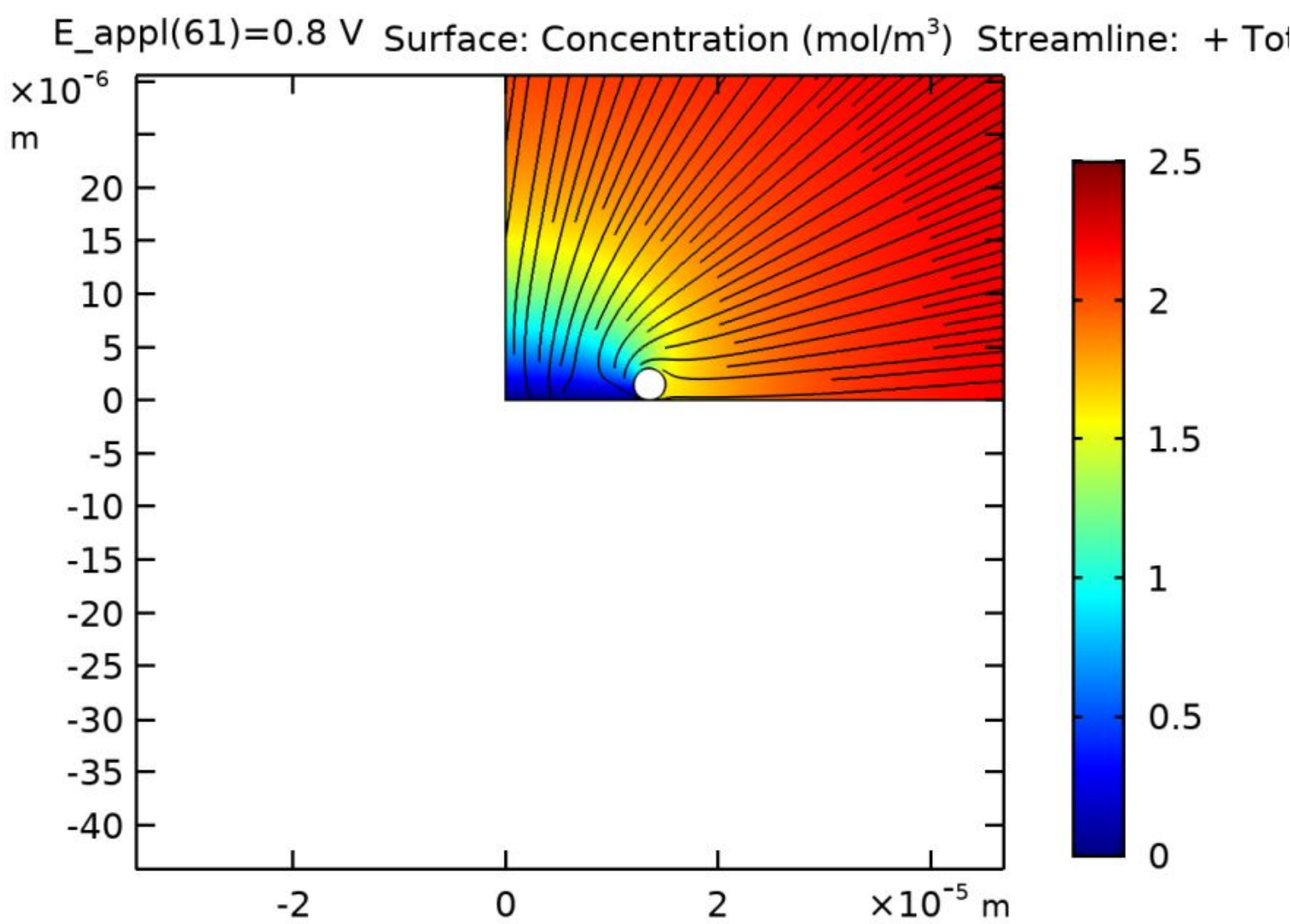

Surface: Concentration (mol/m^3) Streamline: + Total flux

4.3.2 Concentration, 3D (elan)

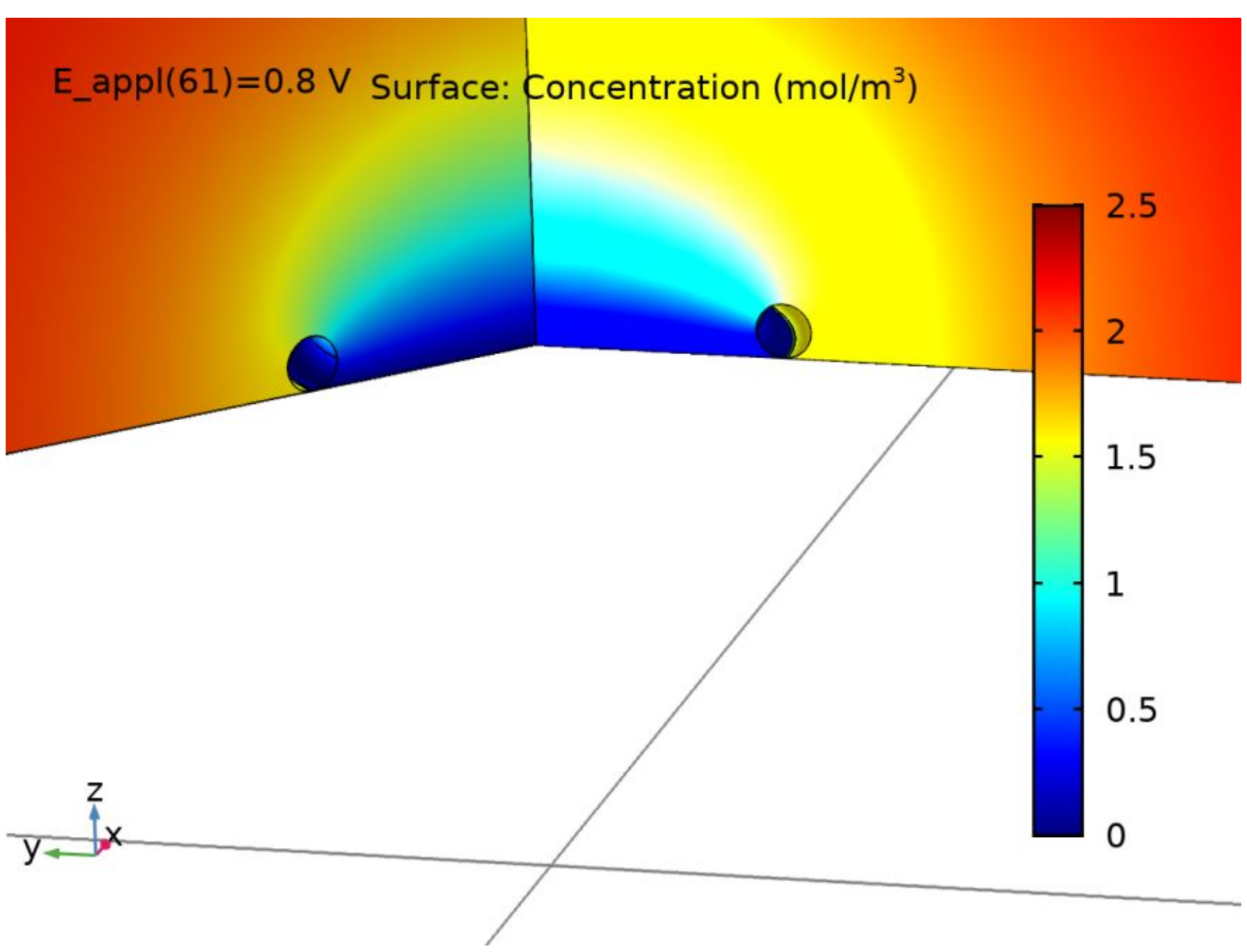

Surface: Concentration (mol/m^3)

4.3.3 1D Plot Group 3

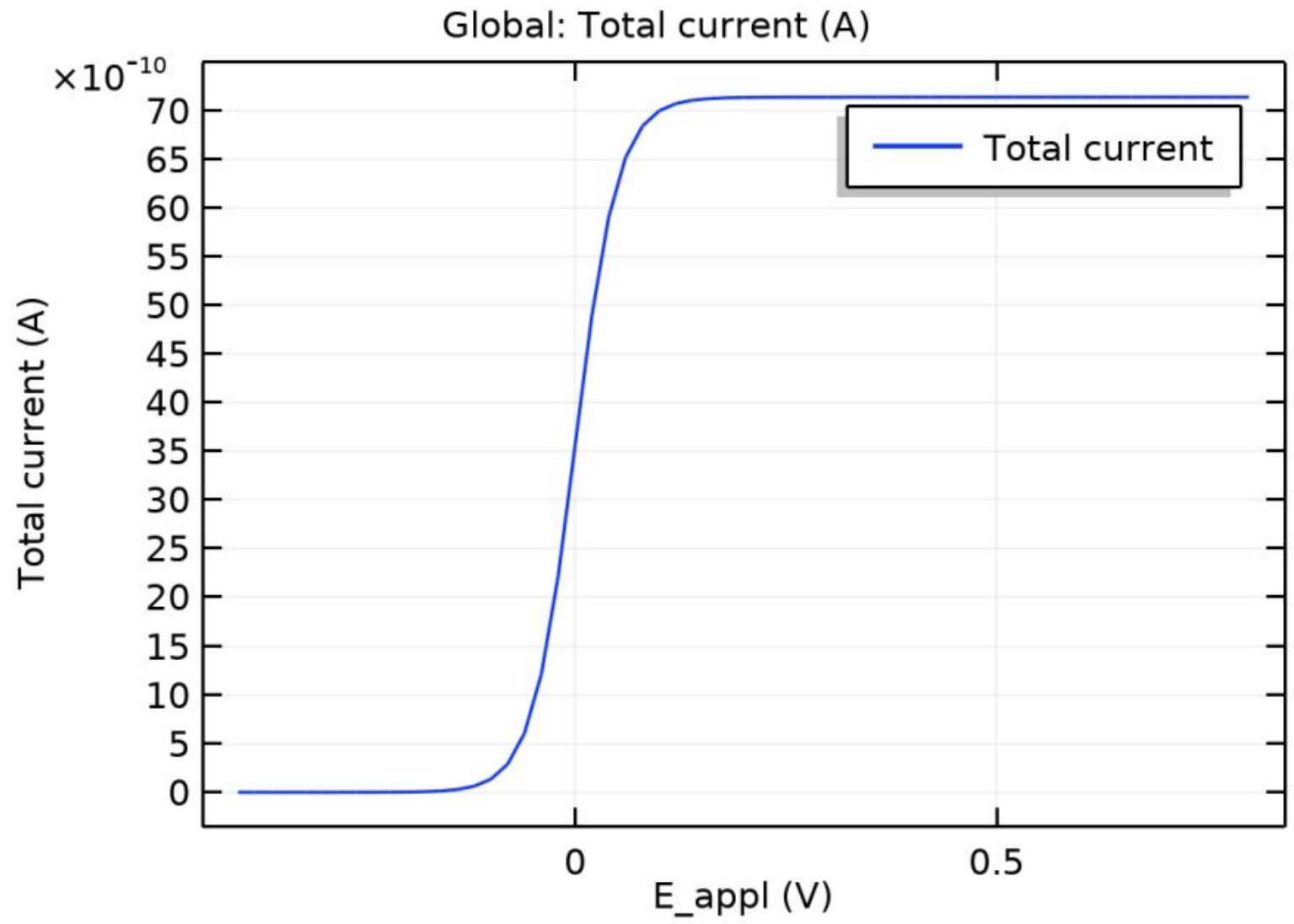

Global: Total current (A)
